# Effectiveness of a self-managed digital exercise programme to prevent falls in older community-dwelling adults: study protocol for the Safe Step randomised controlled trial

**DOI:** 10.1136/bmjopen-2019-036194

**Published:** 2020-05-17

**Authors:** Beatrice Pettersson, Lillemor Lundin-Olsson, Dawn A Skelton, Per Liv, Magnus Zingmark, Erik Rosendahl, Marlene Sandlund

**Affiliations:** 1 Department of Community Medicine and Rehabilitation, Physiotherapy, Umeå University, Umeå, Sweden; 2 School of Health and Life Sciences, Glasgow Caledonian University, Glasgow, UK; 3 Department of Public Health and Clinical Medicine, Section of Sustainable Health, Umeå University, Umeå, Sweden; 4 Health and Social Care Administration, Municipality of Östersund, Östersund, Sweden; 5 Department of Epidemiology and Global Health, Umeå University, Umeå, Sweden

**Keywords:** preventive medicine, statistics & research methods, public health, geriatric medicine

## Abstract

**Introduction:**

Exercise interventions have a strong evidence base for falls prevention. However, exercise can be challenging to implement and often has limited reach and poor adherence. Digital technology provides opportunities for both increased access to the intervention and support over time. Further knowledge needs to be gained regarding the effectiveness of completely self-managed digital exercise interventions. The main objective of this study is to compare the effectiveness of a self-managed digital exercise programme, Safe Step, in combination with monthly educational videos with educational videos alone, on falls over 1 year in older community-dwelling adults.

**Methods and analysis:**

A two-arm parallel randomised controlled trial will be conducted with at least 1400 community-living older adults (70+ years) who experience impaired balance. Participants will be recruited throughout Sweden with enrolment through the project website. They will be randomly allocated to either the Safe Step exercise programme with additional monthly educational videos about healthy ageing and fall prevention, or the monthly education videos alone. Participants receiving the exercise intervention will be asked to exercise at home for at least 30 min, 3 times/week with support of the Safe Step application. The primary outcome will be rate of falls (fall per person year). Participants will keep a fall calendar and report falls at the end of each month through a digital questionnaire. Further assessments of secondary outcomes will be made through self-reported questionnaires and a self-test of 30 s chair stand test at baseline and 3, 6, 9 and 12 months after study start. Data will be analysed according to the intention-to-treat principle.

**Ethics and dissemination:**

Ethical approval was obtained by The Regional Ethical Review Board in Umeå (Dnr 2018/433-31). Findings will be disseminated through the project web-site, peer-reviewed journals, national and international conferences and through senior citizen organisations’ newsletters.

**Trial registration number:**

NCT03963570.

**Strengths and limitations of this study**

- The method of using a completely self-managed digital exercise programme for delivering fall prevention exercise is novel and will contribute to the evidence of using smartphones and tablets for delivering fall prevention exercise.
- The Safe Step-exercise programme includes behaviour change support to facilitate maintenance of the new exercise routine.
- The use of wide inclusion criteria could enhance external validity.
- A limitation of the study is that large attrition rates are expected due to the study design of no face-to-face interaction, and there is a risk of unequal distribution between groups.
- To assess intervention reach, the sociodemographic parameters of participants will be compared with the target population.

## Background

About one-third of community-living older adults fall every year,^1 2^ with an increasing frequency with a higher age and frailty level.^3^ In Sweden, falls represent 87% of injury-related hospital admissions in older adults,^4^ with a 30% risk of another fall which requires medical attention in the next 5 years.^5^ Globally, the related healthcare costs are substantial,^6^ and are likely to exponentially increase with the growing proportion of older people.^7^

There is strong evidence that falls among community-living older adults can be prevented both short and long terms using exercise as a single intervention.^8 9^ The most effective types of exercise are balance and functional exercises combined with resistance exercises.^9^ Even though there is a large body of evidence that supports the effectiveness of exercise interventions, up to 52% of older adults engaging in individually targeted fall prevention exercise do not adhere to the exercise over 12 months.^10^

To aid older adults in changing their exercise behaviour, there is a need for tailored exercise interventions that are not only adapted to the individual’s physical function but also promote personal benefits, such as increased or prolonged independence.^11 12^ Furthermore, interventions need to increase motivation for exercise by taking into consideration the older adults’ individual lifestyle, perceived risks of exercise, as well as physical capabilities.^13^ To support self-efficacy for exercise, older adults can be engaged in creating and tailoring their own routines, which has been suggested as a route to improved adherence.^11^ Additionally, support of older adults’ active roles are preferable to having complete dependence on professionals,^7^ and can also improve well-being among older adults.^14^

Home-based exercise provides a practical choice for participants and could solve issues with both accessibility and costs as well as support the older person to self-manage their exercise. According to a large survey, home-based exercises were preferred over other fall prevention strategies, especially in individuals of an older age, those who have recently fallen, or those who are socially disadvantaged.^15^ To exercise at home with occasional visits from instructors has been shown to be effective in preventing falls^16^ as well as to integrate the exercise into everyday activities at home,^17^ which many older adults also prefer.^18^

The use of technology and digital health is emerging as ways of delivering self-managed exercise and fall prevention interventions for older adults.^19–21^ Such digital interventions enable the participants to exercise at preferred times and locations.^22 23^ Technology can further enable the use of tools for behaviour change such as self-monitoring, direct feedback and action planning, which can support self-efficacy for self-management.^24^ Digital technology can, therefore, provide support over time, which is important due to high attrition rates from fall prevention exercise programmes.^10^

There is limited evidence of the effectiveness of smartphones and tablets for delivering fall prevention exercise.^19^ Of those evaluated, the interventions has been combined with the interaction of health professionals.^20 21^ If proven effective, completely self-managed digital interventions with no interaction with a health professional would provide a more rapid and extensive implementation of fall prevention programmes, vastly increasing reach to a wider population. Additionally, it may be economically beneficial due to reduced use of resources from healthcare professionals. In this emerging field, more research is needed regarding the effectiveness of digital interventions with no interaction between healthcare professionals and participants.

We have previously developed a completely self-managed exercise application for smartphones and tablets with a focus on improving physical function and preventing falls, the Safe Step application.^25^ The application provides a large repository of evidence-based exercises in short video formats with associated safety advice. Behaviour change is supported through strengthening of self-efficacy and behaviour change techniques incorporated in the application. The participants are supported to create their individual programme, plan their weekly exercise and monitor their exercise routine in the application. To further support behaviour change, the participants receive motivational feedback and reminders of their planned exercise from a virtual physiotherapist. The exercise programme is influenced by the Otago home exercise programme that has been proven effective in reducing falls among community-living older adults,^26 27^ but also expanded with both easier and more challenging exercises mainly inspired by the Falls Management Exercise programme.^28^ The Safe Step application (v1) was explored in a feasibility study, a participant preference trial comparing the programme to a paper booklet with exercises. Results from a qualitative study showed that the participant using the digital exercise programme found it both easy to use and easy to tailor to their own circumstances.^22^ The majority of the participants exercised at least during 75% of the intervention weeks and one-third of the participants completed more than 75% of recommended exercise time.^29^

To investigate the effectiveness of a self-managed digital exercise programme, this randomised (1:1) controlled trial (RCT) aims to compare the Safe Step application in combination with monthly educational videos with a control group that only receives the educational videos.

The primary aim is to evaluate the effect on fall rate in older community-dwelling adults during a 12-month intervention period. Secondary aims are to evaluate number of fallers, self-reported balance and strength, fear of falling, quality of life, cost-effectiveness, and attrition and exercise adherence. In addition, we will describe sociodemographic parameters of those who are recruited in comparison to the target population in order to assess intervention reach.

## Method

### Participant and public involvement

The Safe Step application has been designed and cocreated with, and for older adults.^25^ In further cocreation sessions, behaviour change components were developed together with older adults to enhance long-term adherence. The feasibility and acceptability of outcome measures have been informed by a feasibility trial. The Prevention of Falls Network Europe (ProFaNE) consensus of a common data set for fall injury prevention trials^30^ and a feasibility study have informed the choice of outcome measures in this RCT. In addition, a small reference group has tested the final questionnaires and provided feedback. A spokesperson for senior citizen organisations in Sweden was consulted regarding recruitment for the RCT study.

### Study design

The 1-year Safe Step RCT study uses a two-arm parallel design with digital interventions. The intervention group receives the Safe Step application, delivered on smartphones or tablets. Both groups will receive an email every month with short educational videos about healthy ageing and falls prevention.

This protocol is reported in line with the Standard Protocol Items: Recommendations for Interventional Trials guidelines.^31 32^ The RCT will be reported according to the Consolidated Standards of Reporting Trials (CONSORT) statement,^33^ and CONSORT EHEALTH criteria.^34^ The health economic evaluation will be reported according to the Consolidated Health Economic Evaluation Reporting Standards statement.^35^

### Participants

At least 1400 and up to 2000 older adults living in the community will be recruited throughout Sweden. The inclusion and exclusion criteria are shown in table 1.

**Table 1:** Inclusion and exclusion criteria

Inclusion criteria	Exclusion criteria
Older adults 70 years or older Fallen or experienced a decline in perceived postural balance during the last year Have access to a smartphone or tablet and uses it regularly Have an own email address and uses it Able to understand verbal and written instructions in Swedish Can rise from a standard height chair without a person helping Can walk independently without a walking aid indoors	Progressive disease where there is likely to be a decline in strength or balance over the next year Perceived memory dysfunction that affect everyday life activities Taking part in more than 3 hours each week of strenuous physical exercise which makes them out of breath (eg, dance, gymnastics, gym exercises, running or skiing)

### Recruitment

**Figure 1:**
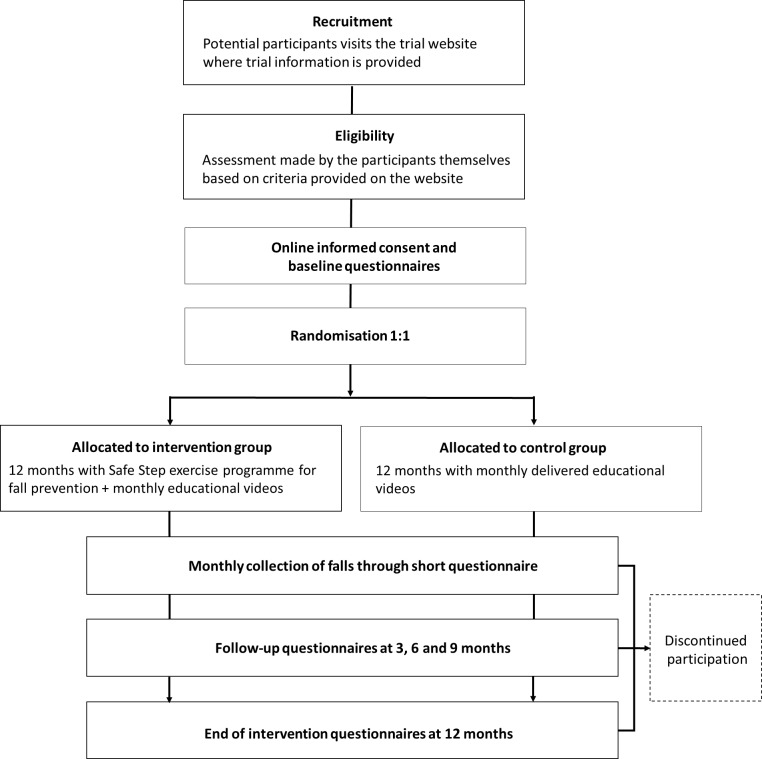
Flow chart of study design.

To reach the target sample size, at least 1 year is estimated for enrolment of participants starting in September 2019. The recruitment strategies will be directed towards the largest senior citizen organisations with the potential to reach approximately 800 000 members, and information will be distributed via the physiotherapist union, section for older adult’s health. Recruitment strategies will include both digital and non-digital strategies. The digital recruitment strategies include advertisements on webpages and Facebook; posts on open and closed Facebook-pages; emails or digital newsletters directly to members of the senior citizen organisations. Non-digital strategies include advertisements in newspapers and in Swedish magazines commonly read by older adults. Participants will be directed to the open-access project website (www.sakrasteg.se) where the necessary study information is provided to decide on participation. A self-assessment of eligibility will be performed by the participant based on the inclusion and exclusion criteria provided (table 1). On the project website, participants are informed that by providing their email address they will give their informed consent for participation and consent for publication of their pseudonymised data. An email will be sent to them to confirm their registration and verify their email address, including one reminder. When confirmation has been received, a link will be sent to the first baseline questionnaire (figure 1).

### Randomisation and blinding

After completed baseline questionnaire, the participants will be randomised in a 1:1 ratio using permuted block randomisation with randomly varied block sizes. A statistician not involved in the study will generate the allocation list using the randomizeR R-package before enrolment.^36^ A computer programme developed in FLOW, Microsoft Office 365-software, will randomise participants according to the allocation list.

After randomisation, the FLOW programme will automatically send emails with follow-up questionnaires as well as the monthly fall reports and educational videos. In order to include participants, monitor data collection and answer emails regarding technical issues, two members of the research group will be able to see the participant’s email addresses and group allocation in the FLOW system and database. However, no personal contact will occur between researchers and participants regarding the results of their outcome assessments.

Analyses of the outcomes regarding falls and physical function will be made by a researcher blinded to group allocation. Participants will not be blinded due to the nature of the intervention.

### Interventions

After group allocation, the participants will receive an email introducing them to the interventions as described next. Throughout the study, there will be a project email account for questions, and frequently asked questions may gradually be added on the website.

#### Safe Step: digital exercise programme

The Safe Step application was developed by our research group in collaboration with older adults^25 37^ and the software (v2) was programmed by Information and Communications Technology Services and System Development (ITS), Umeå University. Participants allocated to the exercise intervention will receive instructions by email on how to download the application from App Store or Google Play, as well as their personal log-in details.

On the first time use of the application, the participants view an instruction video on how to use the application to create and progress their own individual exercise programme. The participants compose their individual exercise programme choosing 10 exercises from predetermined groups of exercises with a main focus to improve balance (3 groups), increase lower limb strength (4 groups) and improve gait/step (3 groups). The exercises are presented in video format and in increasing levels of difficulty, or with alternatives within each group of exercises. The participants are instructed to choose exercises that are challenging but not too hard. Hence, during balance exercises a feeling of being unstable but not to the extent of losing their balance and falling, and for strength exercises progress when able to easily perform the recommended repetitions. A new exercise should be selected whenever they feel that the current exercise becomes too easy or too difficult. Participants are requested to exercise for 30 min at least 3 days/week, but are also informed that a longer duration of exercising per week will give a better fall preventive effect. After each session, participants will register their activities in the application: completed exercises, exercise duration, experienced well-being of the day of exercise and experience of levels of intensity for balance and strength exercises.

**Figure 2:**
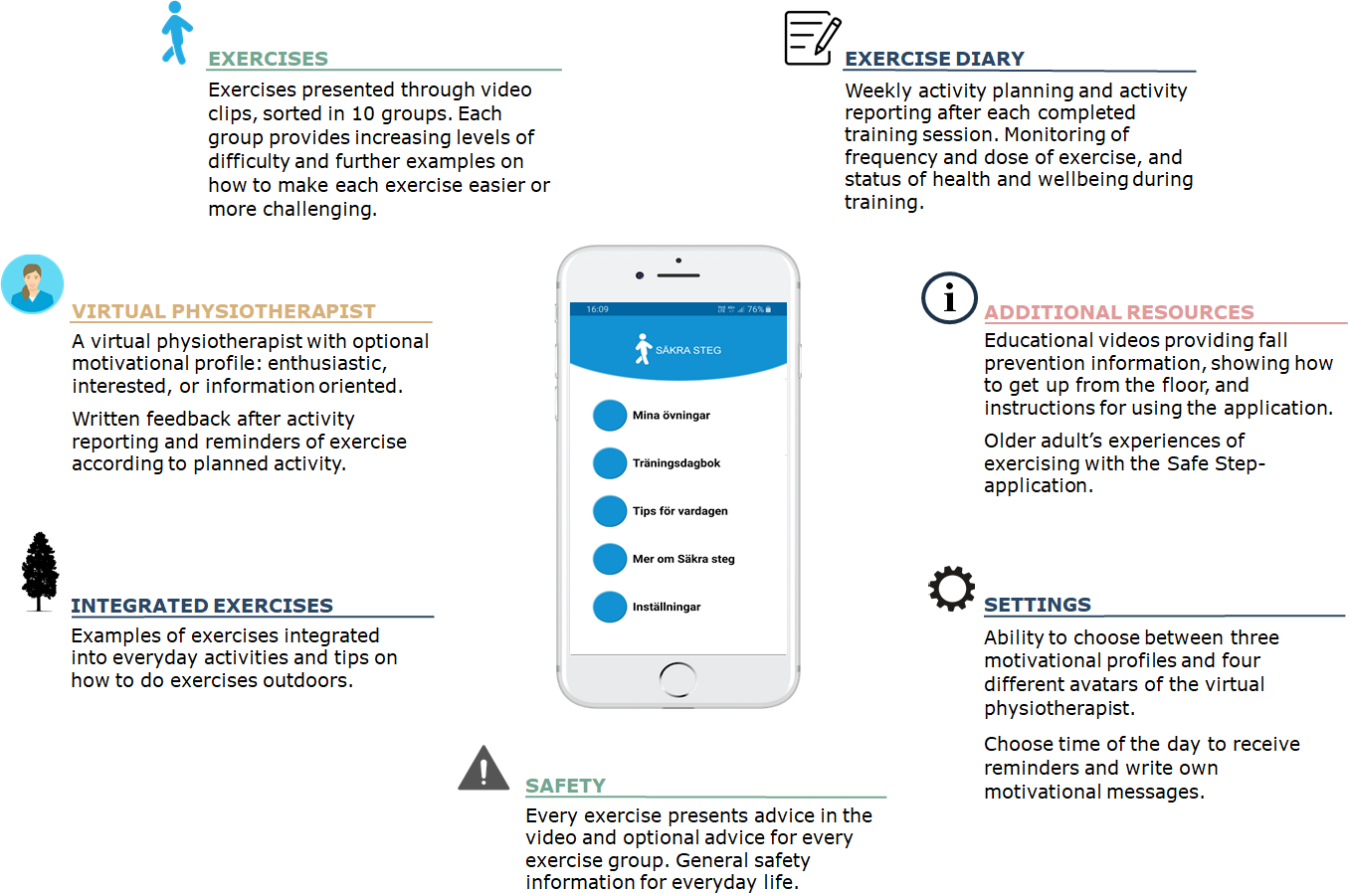
Features of the Safe Step application.

In the application, the participant can make a schedule for their weekly exercise, register activity and monitor their exercise routines (figure 2). They will also be able to set reminders tailored to their personal preferences. Direct feedback and motivational messages will be received from a virtual physiotherapist when exercise is registered. At the beginning of every month of the 1-year intervention period, the participants will receive an email with a new educational video about healthy ageing and fall prevention. The third video contains information on physical activity and exercise, but no suggestions of particular exercises are given.

#### Control group

Participants allocated to the control group will receive the same educational videos as those sent to the intervention group. After the 12-month follow-up assessment, the control group will be offered the Safe Step application.

### Data collection

At baseline and 3, 6, 9 and 12 months, respectively, from the participant’s inclusion in the study, an email containing a link to an online questionnaire will be sent to the participants. All collected data will be self-reports, including a 30 s chair stand self-test (30CST). In the absence of completed questionnaires, a reminding email will be sent out after 1 week. If further absence, another email will be sent after an additional week. This reminder will also include a question if they want to withdraw from the study. Data registered in the exercise diary of the application are collected continuously. In the same monthly email as the educational videos, all participants will receive a request to report any falls during the past month by answering a short web-based questionnaire. In the absence of a completed questionnaire, one reminder will be sent after 1 week. All data retrieved from questionnaires, as well as application data, will be coded and reported on a group level.

### Outcomes

#### Primary outcome

The primary and secondary outcomes, and time points of distribution are presented in table 2. The primary outcome measure will be the rate of falls (fall per person/year) during the 12 months of the intervention. To register falls, a link to a self-report online questionnaire will be sent monthly by email. To be able to report correctly the coming month, participants are asked to make notes of falls in a calendar, preferably the day the fall has occurred. They will be provided with a printable calendar in the email sent out after allocation, but they can also use their own. If a fall is reported, additional questions will be asked about the cause of the fall, potential injuries sustained, need for medical attention and hospitalisation. The number of persons falling during 12 months will be analysed based on the same data as a secondary outcome.

**Table 2:** List of primary and secondary outcome measures

Outcome measures	BA	C	M	3 months	6 months	9 months	12 months
Fall rate† (fall per person/year)			X				
Number of fallers‡ (n)			X				
Number of falls during exercise with Safe Step§ (n)			X				
Fall-related injuries§ (yes/no, need of medical attention, type of injury)			X				
Hospitalisation due to falls§ (yes/no and number of days)			X				
Reach§ (self-reported sociodemographic parameters such as age, sex, years of education, use of smartphone and tablet, number of cohabitants and self-reported health and diagnosis)	X						
Self-administered 30 s chair stand test¶	X			X	X	X	X
The Borg Scale of Perceived Exertion during 30 s chair stand test** (numeric scale, score 6–20)	X			X	X	X	X
Self-rated balance**, ‘How do you perceive your balance?’ (5-level ordinal scale)	X			X	X	X	X
Self-rated leg strength**, ‘How do you perceive your leg muscle strength?’ (5-level ordinal scale)	X			X	X	X	X
Fear of falling¶ (Falls Efficacy Scale-International)	X			X	X	X	X
Health-related quality of life¶ (EQ-5D-5L)	X			X	X	X	X
Experienced positive effects of the exercise intervention§ (nominal scale, optional text field)	X*			X*	X*	X*	X*
Experienced negative effects of the exercise intervention§ (nominal scale, optional text field)	X*			X*	X*	X*	X*
Exercise adherence throughout the intervention reported through an integrated exercise diary in the Safe Step application§,¶		X*					
Exercise adherence, self-reported min/week§				X*	X*	X*	X*
Use and perceived usefulness of educational videos** (4-level ordinal scale)				X	X	X	X
Self-rated improvements in balance††, ‘If you compare with when you started this study a year ago, how would you assess balance today?’ (5-level ordinal scale)							X
Self-rated improvements in leg-strength††, ‘If you compare with when you started this study a year ago, how would you assess the muscle strength in your legs today?’ (5-level ordinal scale)							X
Rate of attrition‡ (n)							X
New exercise routines§ (yes/no, text field to describe the exercise, time since initiation (predefined alternatives), min/week (predefined alternatives))							X
Physical activity¶ (min/week, predefined alternatives)	X						X
Self-reported costs related to the interventions§ (amount and what)							X
Cost effectiveness of the interventions with respect to falls¶							X

*Only administered to the exercise intervention group,

†Negative binomial regression.

‡Logistic regression.

§Descriptive analysis.

¶Linear mixed model.

**Cumulative link mixed model.

††Ordinal logistic regression.

BA, baseline assessment; C, continuously; EQ-5D-5L, European Quality of Life, 5 dimensions; M, monthly administered.

Falls will be defined as an event in which the person comes to rest inadvertently on the ground or floor, regardless of what caused the fall or if an injury was sustained. Our definition is slightly modified and clarified in comparison with a previous consensus statement.^30^ The ProFaNE definition included falls where the person come to rest on an ‘other lower level’, for example, tripping and taking a hold of a dresser.^30^ We exclude ‘lower level’ as our experience from previous studies is that participants seldom perceived such an event as a fall. In accordance with a systematic review,^38^ we clarify that a fall should be reported regardless of cause and we also clarify that unintentionally coming to rest on the floor or ground is a fall regardless of injury. Our definition is printed in the online questionnaire.

#### Secondary outcomes

Sociodemographic parameters, such as age, sex, health, use of smartphones and tablets, and living conditions will be collected at baseline to understand which older adults who are reached through the intervention and to be able to assess predictors of completion.

Self-reported data regarding physical assessments will be collected at baseline, 3 months, 6 months, 9 months and 12 months, respectively. Single-item questions with a 5-graded Likert scale are used to assess self-rated improvements in strength and balance (table 2). A 30 s chair stand test is performed by the participants following instructions in the online questionnaire. The original administration of the test has shown excellent test–retest reliability and criterion validity for assessing functional lower extremity leg strength in older adults,^39^ but studies regarding the psychometric properties of a self-test is lacking. Instructions for the participants to be able to perform the 30CST by themselves at home were developed by the research group. After feedback from an advisory group of older adults, the instructions were slightly revised. When performing the test, the participants are asked to place a chair against the wall and take a seat in the middle of the chair with both feet in full contact with the floor and arms folded across the chest. They are instructed to fully stand up and sit down as many times as possible for 30 s and register the number of full stands. Participants are asked to use the Borg Scale of Perceived Exertion^40^ in combination with the 30CST test to quantify their effort. The perceived exertion is rated on a scale between 6 (none) and 20 (maximal exertion).

The Swedish version of Falls Efficacy Scale-International (FES-I(S))^41^ will be used to measure concerns about falling. FES-I is widely used among older adults and is suitable for use in different populations and in clinical trials.^42^ The FES-I and FES-I (S) have shown excellent internal and test–retest reliability.^41 43^

Health-related quality of life will be included in the secondary outcomes to enable us to conduct economic analysis and will be assessed by the European Quality of Life, 5 dimensions (EQ-5D-5L)^44^ self-report questionnaire. Cost effectiveness will be conducted from a societal perspective at 12 months.

Both groups will also be asked to rate their use and perceived usefulness of the monthly delivered educational videos on 4-level ordinal scales.

Additional questions will be asked to the exercise intervention group regarding adherence, experienced positive effects besides strength and balance, and adverse events. Adherence to exercise will be evaluated by self-report questions at follow-up assessments through predefined alternatives, and will be summarised as exercised min/week. Additionally, adherence to exercise including the proportion meeting at least the predefined minutes of 3×30 min/week, will be collected through an exercise diary integrated within the Safe Step-application.

At self-report assessments at 3, 6, 9 and 12 months after study start, a single-item question with predefined answers will be used to assess experienced positive effects of the exercise intervention besides effects on balance and strength.

Adverse events in form of falls occurring while exercising with the Safe Step application will be monitored monthly. If a participant registers a fall in the monthly questionnaire, there will be a follow-up question of whether the fall occurred while exercising with the Safe Step programme. If so, a member of the research team will contact the participant to ask him or her about the nature of the fall, and if necessary, suggest measures to prevent further falls during exercise. Additional adverse events of taking part in the intervention (eg, muscle strain) will be collected at assessments at 3, 6, 9 and 12 months after study start, and measured through a single-item question: ‘Have you experienced any negative effects of the exercise intervention?’ The participants will be presented with predefined alternatives and an optional text field.

At the end of the 12-month intervention, the participants in both groups will be asked if they have started to do any other exercise activities or programmes during the intervention period. In addition, self-rated improvements in balance and leg strength over the whole course of the intervention will be measured through single-item questions. Both groups will also be asked to report if they have had any extra cost related to the intervention during the intervention period (eg, for internet access or equipment) to inform the cost-effectiveness analysis. Drop-outs and withdrawals from the study will be registered throughout the 12-month intervention. Withdrawals will be defined as those who have formally discontinued the study by filling in the withdraw questionnaire. Participants that consistently discontinue to answer the questionnaires will be defined as drop-outs.

### Data storing and management

Participants will be assigned a pseudonymised identifying code when registering for the study. Email addresses will be collected at registration, but no names or social security numbers. Data from the online questionnaires will be directly entered into LimeSurvey software, integrated with a data collection platform, developed and hosted by ITS at Umeå University. Through LimeSurvey, several data quality features have been implemented, for example, obligatory fields and range checks.

The identity of the participants will be separated from research data entries, that is, questionnaire response and mobile-based exercise diary entries, by storing the identity in a separate database. Access to sensitive information requires a separate set of credentials. ITS at Umeå University provides all web applications, services and storage for the study. This is provided in accordance with research regulations and the General Data Protection Regulation (GDPR). Data entries will be monitored by one of the researchers throughout the study.

### Sample size

The sample size calculation for the primary outcome (fall rate over the 12-month intervention period) is based on the review of Sherrington *et al*^45^ by calculating for a fall rate of 1 fall/person year. In addition, a measure of over dispersion of 1.2 is used.^21^ Thus, we require 420 participants in each group to have an 80% power to detect a 20% reduction in fall rate at a 5% level of significance. We estimate that at least 1400 participants are needed based on a calculated attrition rate of 50% and a contribution of data from drop-outs. Due to uncertain drop-out rate, we will include up to 2000 participants.

### Planned statistical analyses

Analyses will be performed according to the intention-to-treat principle. Thus, all available data for each randomised participant will be analysed according to original allocation and regardless of level of participation. The primary outcome, that is, the number of falls per person year, will be analysed using negative binomial regression to account for an expected overdispersion with respect to Poisson distribution.^46^ The fall rate ratio of the two groups will be estimated with a 95% CI and presented with a p value while adjusting for the prognostic baseline covariates of age and sex for the purpose of increasing precision in estimates and statistical power. In addition, unadjusted results will be presented in accordance with recommendations from the CONSORT 2010 statement.^33^

For secondary outcomes on ordinal variables measured quarterly, analysis will be performed using cumulative link mixed models with logit link function and group, time point and group*timepoint interaction as fixed factors and participant as a random factor. In continuously scored secondary variables measured quarterly, general mixed linear models will be used to assess the group difference. Logistic regression models will be used to compare groups on dichotomous outcome measures.

Effects on falls, attrition and self-reported effects on balance and strength will be studied in subgroup analyses in relation to sex, different age groups, reported health status, physical activity and use of smartphones and tablets. Further subgroup analysis will examine exercise adherence in the exercise group as well as the dose–response relationships (min/week). Descriptive statistics will be provided to reveal the sociodemographic distribution in the sample in comparison to the population and geographical distribution in urban and rural areas.

Multiple imputation will be performed on variables containing data considered to be missing at random if proportions of missing data does not exceed 40%, as suggested in literature.^47^ In addition, analysis on original unimputed data will be presented as supplement. All statistical tests will be two sided with a significance level of 0.05.

### Economic evaluation

Results will be presented as incremental cost-effectiveness ratio after 12 months, that is, the ratio of the difference in costs and the difference in health effects between the intervention group versus the control group. Data on health effects will be based on the primary outcome falls averted and the secondary outcome health-related quality of life assessed by the EQ-5D-5L. The data on health-related quality of life will be used to calculate quality-adjusted life years. Health and social care costs will be estimated by self-reported data on monthly falls and their consequences in combination with previously published data on the specific cost for the most frequent injuries. Resource use will include intervention costs, healthcare costs and community service costs.

### Ethics and dissemination

Ethical approval was obtained by The Regional Ethical Review Board in Umeå (Dnr 2018/433-31). The results of the study will be made publicly available through the project website, reported at national and international conferences, as well as in peer-reviewed journals, and presented to senior citizen organisations through member journals and digital newsletters. Data from the Safe Step-RCT will be available on reasonable request when the results have been published in peer-reviewed journals.

## Discussion

To facilitate appropriate exercise to reduce age-related functional decline and prevent falls, attractive and evidence-based interventions are needed that can reach older people, including those who live rurally, or who merely want to self-manage their risk of falling. This study will evaluate the effectiveness of a completely self-managed digital exercise programme for fall prevention with additional educational videos compared to educational videos only.

The Safe Step application is available for smartphones and tablets and provides evidence-based exercises for the home environment. Besides conventional exercises, Safe Step inspires the integration of exercises in daily activities, both indoors and outdoors. The approach of integrating exercise into already existing daily routines has shown great potential to reduce the rate of falls among older adults.^17^

To support the older adults to sustain their exercise behaviour over time, Safe Step includes a set of behaviour change strategies. The older users will create their individual exercise programme, plan their exercise and monitor their exercise routines in order to support exercise self-management. Self-management of health and exercise has been recommended to improve adherence to fall prevention programmes by strengthening the older adults’ self-efficacy.^11^ However, delivering exercise by mobile technology increases the complexity of providing individual support. Promisingly, in our previous feasibility study, participants expressed confidence in self-managing their exercise programmes, both in digital format and as a standard paper booklet. Worth noting is that use of the Safe Step-exercise programme seems to have supported the older adults to a greater extent than a paper booklet.^22^

Adherence to individual exercise-based fall prevention is often poor over time.^10^ A contributing factor could be a lack of social components and peer support, which has been shown to positively affect attendance and adherence to exercise classes among older adults.^48^ However, preferences are diverse among older adults, and some older adults have stated to prefer home-based exercises.^15^ Due to the nature of this home-based intervention with online recruitment and an intervention period of 1 year, an attrition rate of 50% is anticipated. Still, this study will assess the effectiveness of a self-managed digital exercise programme to prevent falls in real-life settings where the older adults themselves will take the initiative to download and use an application. Hence, the attrition rates will be a valuable contribution in understanding more about older adult’s exercise adherence when exercising with smartphones and tablets in a home-based setting.

Home-based exercise has been shown to be preferred among older adults of advancing age, those with recent falls, and those with lower socioeconomic status.^15^ On one hand, fall prevention interventions delivered by digital technology might create a constraint for older adults who cannot afford digital technology or do not have digital competency. On the other hand, digital technology will present opportunities for older adults that might not have the opportunity or interest in attending community-based programmes or regularly visit healthcare centres. Additionally, in recent years, the use of digital technology such as smartphones and tablets has increased sharply among older adults. In 2019, 67% of the aged Swedish population used the internet on their smartphone, and 40% on their tablet,^49^ and the rapid increase in use is expected to continue. In light of these considerations, it is important to explore reach as suggested in the present study to describe which older adults that are interested in exercising with a digital application with no interaction with a healthcare professional.

Besides effect on falls, this research has the potential to increase knowledge regarding which older adults are interested in a digital self-management exercise intervention, exercise adherence and effects on self-rated physical function. The design of this study with few inclusion and exclusion criteria and a national recruitment will strengthen external validity in developed countries similar to Sweden with an ageing population and large rural areas. If proven effective and cost effective, the Safe Step digital exercise programme has the potential to be highly accessible and reduce risk of functional decline and falls.

## Supplementary Material

Reviewer comments

Author's manuscript
