# Supplementary material for: Effectiveness of a self-managed digital exercise programme to prevent falls in older community-dwelling adults: study protocol for the Safe Step randomised controlled trial
Source: BMJ Open. 2020 May 17;10(5):e036194. doi: 10.1136/bmjopen-2019-036194 (PMC7239551; doi:10.1136/bmjopen-2019-036194)
Supplement: Author's manuscript [file bmjopen-2019-036194.draft_revisions.pdf]

BMJ Open is committed to open peer review. As part of this commitment we make the peer review history of every article we publish publicly available.

When an article is published we post the peer reviewers' comments and the authors' responses online. We also post the versions of the paper that were used during peer review. These are the versions that the peer review comments apply to.

The versions of the paper that follow are the versions that were submitted during the peer review process. They are not the versions of record or the final published versions. They should not be cited or distributed as the published version of this manuscript.

BMJ Open is an open access journal and the full, final, typeset and author-corrected version of record of the manuscript is available on our site with no access controls, subscription charges or pay-per-view fees (http://bmjopen.bmj.com).

If you have any questions on BMJ Open's open peer review process please email info.bmjopen@bmj.com

BMJ Open

# Effectiveness of a self-managed digital exercise programme to prevent falls in older community-dwelling adults –study protocol for the Safe Step randomized controlled trial

| | |
|---|---|
| Journal: | *BMJ Open* |
| Manuscript ID | bmjopen-2019-036194 |
| Article Type: | Protocol |
| Date Submitted by the Author: | 04-Dec-2019 |
| Complete List of Authors: | Pettersson, Beatrice; Umeå University, Department of Community Medicine and Rehabilitation, Physiotherapy<br>Lundin-Olsson, Lillemor; Umeå University, Department of Community Medicine and Rehabilitation, Physiotherapy<br>Skelton, Dawn; Glasgow Caledonian University, School of Health & Life Sciences<br>Liv, Per; Umeå University, Department of Public Health and Clinical Medicine, Section of Sustainable Health<br>Zingmark, Magnus ; Municipality of Östersund, Health and Social Care Administration; Umeå University, Department of Epidemiology and Global Health<br>Rosendahl,  Erik; Umeå University, Department of Community Medicine and Rehabilitation, Physiotherapy<br>Sandlund, Marlene; Umeå University, Department of Community Medicine and Rehabilitation, Physiotherapy |
| Keywords: | PREVENTIVE MEDICINE, STATISTICS & RESEARCH METHODS, PUBLIC HEALTH, GERIATRIC MEDICINE |
| | |

SCHOLARONE™
Manuscripts

BMJ

*I, the Submitting Author has the right to grant and does grant on behalf of all authors of the Work (as defined in the below author licence), an exclusive licence and/or a non-exclusive licence for contributions from authors who are: i) UK Crown employees; ii) where BMJ has agreed a CC-BY licence shall apply, and/or iii) in accordance with the terms applicable for US Federal Government officers or employees acting as part of their official duties; on a worldwide, perpetual, irrevocable, royalty-free basis to BMJ Publishing Group Ltd ("BMJ") its licensees and where the relevant Journal is co-owned by BMJ to the co-owners of the Journal, to publish the Work in this journal and any other BMJ products and to exploit all rights, as set out in our licence.*

*The Submitting Author accepts and understands that any supply made under these terms is made by BMJ to the Submitting Author unless you are acting as an employee on behalf of your employer or a postgraduate student of an affiliated institution which is paying any applicable article publishing charge ("APC") for Open Access articles. Where the Submitting Author wishes to make the Work available on an Open Access basis (and intends to pay the relevant APC), the terms of reuse of such Open Access shall be governed by a Creative Commons licence – details of these licences and which Creative Commons licence will apply to this Work are set out in our licence referred to above.*

*Other than as permitted in any relevant BMJ Author's Self Archiving Policies, I confirm this Work has not been accepted for publication elsewhere, is not being considered for publication elsewhere and does not duplicate material already published. I confirm all authors consent to publication of this Work and authorise the granting of this licence.*

For peer review only

# Title

Effectiveness of a self-managed digital exercise programme to prevent falls in older community-dwelling adults –study protocol for the Safe Step randomized controlled trial

# Authors

**Beatrice Pettersson**, Department of Community Medicine and Rehabilitation, Physiotherapy, Umeå University, Umeå, Sweden. beatrice.pettersson@umu.se **(corresponding author)**

**Lillemor Lundin-Olsson**, Department of Community Medicine and Rehabilitation, Physiotherapy, Umeå University, Umeå, Sweden. lillemor.lundin-olsson@umu.se

**Dawn A Skelton**, School of Health and Life Sciences, Glasgow Caledonian University, Glasgow, UK. dawn.skelton@gcu.ac.uk

**Per Liv**, Department of Public Health and Clinical Medicine, Section of Sustainable Health, Umeå University, Umeå, Sweden. per.liv@umu.se

**Magnus Zingmark**, [a]Municipality of Östersund, Health and Social Care Administration, Östersund, Sweden, [b]Department of Epidemiology and Global Health, Umeå University, Sweden. magnus.zingmark@ostersund.se

**Erik Rosendahl**, Department of Community Medicine and Rehabilitation, Physiotherapy, Umeå University, Umeå, Sweden. erik.rosendahl@umu.se

**Marlene Sandlund**, Department of Community Medicine and Rehabilitation, Physiotherapy, Umeå University, Umeå, Sweden. marlene.sandlund@umu.se

**Word count: 4628**



For peer review only

For peer review only - http://bmjopen.bmj.com/site/about/guidelines.xhtml

# ABSTRACT

## Introduction

Exercise interventions have a strong evidence base for falls prevention. However, exercise can be challenging to implement and often has limited reach and poor adherence. Digital technology provides opportunities for both increased access to the intervention and support over time. Further knowledge needs to be gained regarding the effectiveness of completely self-managed digital exercise interventions. The main objective of this study is to compare the effectiveness of a self-managed digital exercise programme, Safe Step, in combination with monthly educational videos with educational videos alone, on falls over one year in older community-dwelling adults.

## Methods and analysis

A two-arm parallel randomized controlled trial will be conducted with at least 1400 community-living older adults (70+ years) who experience impaired balance. Participants will be recruited throughout Sweden with enrolment through the project website. They will be randomly allocated to either the Safe Step exercise programme with additional monthly educational videos about healthy ageing and fall prevention, or the monthly education videos alone. Participants receiving the exercise intervention will be asked to exercise at home for at least 30 minutes, 3 times/week with support of the Safe Step-application. The primary outcome will be rate of falls (fall per person-year). Participants will keep a fall calendar and report falls at the end of each month through a digital questionnaire. Further assessments of secondary outcomes will be made through self-reported questionnaires and a self-test of 30-sec chair stand test at baseline and 3, 6, 9 and 12 months after study start. Data will be analysed according to the intention-to-treat principle.

## Ethics and dissemination

Ethical approval was obtained by The Regional Ethical Review Board in Umeå (Dnr 2018/433-31). Findings will be disseminated through peer-reviewed journals, national and international conferences, and through senior citizens organizations.

## Trial registration number

NCT03963570


Bullet points, stenghts and limitations of the study:

- The method of using a completely self-managed digital exercise program for delivering fall prevention exercise is novel and will contribute to the evidence of using smartphones and tablets for delivering fall prevention exercise.
- The Safe Step-exercise program includes behaviour change support to facilitate maintenance of the new exercise routine.
- The use of wide inclusion criteria could enhance external validity.
- A limitation of the study is that large attrition rates are expected due to the study design of no face-to-face interaction, and there is a risk of unequal distribution.
- To assess intervention reach, the socio-demographic parameters of participants will be compared to the target population.

For peer review only



For peer review only - http://bmjopen.bmj.com/site/about/guidelines.xhtml

# BACKGROUND

About one-third of community-living older adults fall every year(1, 2), with an increasing frequency with a higher age and frailty level.(3) In Sweden, falls represent 87% of injury-related hospital admissions in older adults(4), with a 30% risk of another fall which requires medical attention in the next five years.(5) Globally, the related health care costs are substantial(6), and is likely to exponentially increase with the growing proportion of older people.(7)

There is strong evidence that falls among community-living older adults can be prevented both short- and long term using exercise as a single intervention.(8, 9) The most effective types of exercise are balance and functional exercises combined with resistance exercises.(9) Even though there is a large body of evidence that supports the effectiveness of exercise interventions, up to 52% of older adults engaging in individually targeted fall prevention exercise do not adhere to the exercise over 12 months.(10)

To aid older adults in changing their exercise behaviour, there is a need for tailored exercise interventions that are not only adapted to the individual's physical function but also promote personal benefits, such as increased or prolonged independence.(11, 12) Furthermore, interventions need to increase motivation for exercise by taking into consideration the older adults' individual lifestyle, perceived risks of exercise, as well as physical capabilities.(13) To support self-efficacy for exercise, older adults can be engaged in creating and tailoring their own routines, which has been suggested as a route to improved adherence.(11) Additionally, support of older adults' active roles are preferable to having complete dependence on professionals,(7) and can also improve well-being among older adults.(14)

Home-based exercise provides a practical choice for participants and could solve issues with both accessibility and costs as well as support the older person to self-manage their exercise. According to a large survey, home-based exercises were preferred over other fall prevention strategies, especially in individuals of an older age, those who have recently fallen, or those who are socially disadvantaged.(15) To exercise at home with occasional visits from instructors has been shown to be effective in preventing falls(16) as well as to integrate the exercise into everyday activities at home(17), which many older adults also prefer.(18)

The use of technology and digital health are emerging as ways of delivering self-managed exercise and fall prevention interventions for older adults.(19-21) Such digital interventions enable the participants to exercise at preferred times and locations.(22, 23) Technology can further enable the use of tools for behaviour change such as self-monitoring, direct feed-back, and action planning, which can support self-efficacy for self-management.(24) Digital technology can, therefore, provide support over time, which is important due to high attrition rates from fall prevention exercise programmes.(10)

There is limited evidence of the effectiveness of smartphones and tablets for delivering fall prevention exercise.(19) Of those evaluated, the interventions has been combined with the interaction of health professionals, e.g.(20, 21). If proven effective, completely self-managed digital interventions with no interaction with a health professional would provide a more rapid and extensive implementation of fall prevention programmes, vastly increasing reach to a wider population. Additionally, it may be economically beneficial due to reduced use of resources from health care professionals. In this emerging field, more research is needed regarding the effectiveness of digital interventions with no interaction between health care professionals and participants.



For peer review only - http://bmjopen.bmj.com/site/about/guidelines.xhtml

We have previously developed a completely self-managed exercise application for smartphones and tablets with a focus on improving physical function and preventing falls, the Safe Step-application.(25) The application provides a large repository of evidence-based exercises in short video formats with associated safety advice. Behaviour change is supported through strengthening of self-efficacy and behaviour change techniques incorporated in the application. The participants are supported to create their individual programme, plan their weekly exercise and monitor their exercise routine in the application. To further support behaviour change, the participants receive motivational feedback and reminders of their planned exercise from a virtual physiotherapist. The exercise programme is influenced by the Otago home exercise programme that has been proven effective in reducing falls among community-living older adults,(26, 27) but also expanded with both easier and more challenging exercises mainly inspired by the Falls Management Exercise (FaME) programme.(28) The Safe Step-application and participant's experiences has been explored in a patient preference trial, comparing the programme to a paper booklet with exercises.(22)

To investigate the effectiveness of a self-managed digital exercise programme, this randomised (1:1) controlled trial (RCT) aims to compare the Safe Step-application in combination with monthly educational videos with a control group that only receives the educational videos.

The primary aim is to evaluate the effect on fall rate in older community-dwelling adults during a 12 month intervention period. Secondary aims are to evaluate number of fallers, self-reported balance and strength, fear of falling, quality of life, cost-effectiveness, and attrition and exercise adherence. In addition, we will describe socio-demographic parameters of those who are recruited in comparison to the target population in order to assess intervention reach.

# METHOD

## Participant and Public Involvement

The Safe Step-application has been designed and co-created with, and for older adults.(25) In further co-creation sessions, behaviour change components were developed together with older adults to enhance long-term adherence. The feasibility and acceptability of outcome measures have been informed by a feasibility trial. The ProFaNE consensus of a common data set for fall injury prevention trials(29) and a feasibility study have informed the choice of outcome measures in this RCT. In addition, a small reference group has tested the final questionnaires and provided feedback. A spokesperson for senior citizens organisations in Sweden was consulted regarding recruitment for the RCT-study.

## Study design

The 1-year Safe Step RCT-study uses a two-arm parallel design with digital interventions. The intervention group receives the Safe Step-application, delivered on smartphones or tablets. Both groups will recieve an e-mail every month with short educational videos about healthy ageing and falls prevention.

This protocol is reported in line with the Standard Protocol Items: Recommendations for Interventional Trials (SPIRIT) guidelines.(30, 31) The RCT will be reported according to the Consolidated Standards of Reporting Trials (CONSORT) statement,(32) and CONSORT EHEALTH criteria.(33) The health economic evaluation will be reported according to the Consolidated Health Economic Evaluation Reporting Standards (CHEERS) statement.(34) The trial was registered prior to study start at ClinicalTrials.gov (NCT03963570).



For peer review only - http://bmjopen.bmj.com/site/about/guidelines.xhtml

## Participants

At least 1400 and up to 2000 older adults living in the community will be recruited throughout Sweden. The inclusion and exclusion criteria are shown in table 1.

**Table 1.** Inclusion and exclusion criteria

| Inclusion criteria | Exclusion criteria |
|---|---|
| • Older adults 70 years or older<br>• Fallen or experienced a decline in perceived postural balance during the last year<br>• Have access to a smartphone or tablet and uses it regularly<br>• Have an own e-mail address and uses it<br>• Able to understand verbal and written instructions in Swedish<br>• Can rise from a standard height chair without a person helping<br>• Can walk independently without a walking aid indoors | • Progressive disease where there is likely to be a decline in strength or balance over the next year<br>• Perceived memory dysfunction that affect everyday life activities<br>• Taking part in more than 3 hours each week of strenuous physical exercise which makes them out of breath (e.g. dance, gymnastics, gym exercises, running, or skiing) |

## Recruitment

To reach the target sample size, one year is estimated for enrolment of participants (Sept 2019-Sept 2020). Recruitment will be performed by advertisements in newspapers and Swedish magazines commonly read by older adults, through the largest Senior Citizens Organisations with the potential to reach approximately 775 000 members, social media, and through the Physiotherapist union, section for older adult's health. Participants will be directed to the open-access project website (www.sakrasteg.se) where study information is provided. A self-assessment of eligibility will be performed by the participant based on the inclusion and exclusion criteria provided (table 1). On the project website, participants are informed that by providing their e-mail address they will give their informed consent for participation and consent for publication of their pseudonymised data. An e-mail will be sent to them to confirm their registration and verify their e-mail address, including one reminder. When confirmation has been received a link will be sent to the first baseline questionnaire (figure 1).

## Randomization and Blinding

After completed baseline questionnaire, the participants will be randomized in a 1:1 ratio using Permuted Block Randomization with randomly varied block sizes. A statistician not involved in the study will generate the allocation list using the randomizeR R-package before enrolment.(35) A computer programme developed in FLOW, Microsoft Office 365-software, will randomize participants according to the allocation list.

After randomization, the FLOW programme will automatically send e-mails with follow-up questionnaires as well as the monthly fall reports and educational videos. In order to include



For peer review only

For peer review only - http://bmjopen.bmj.com/site/about/guidelines.xhtml

participants, monitor data collection, and answer e-mails regarding technical issues, two members of the research group will be able to see the participant's e-mail addresses and group allocation in the FLOW-system and database. However, no personal contact will occur between researchers and participants regarding the results of their outcome assessments.

Analyses of the outcomes regarding falls and physical function will be made by a researcher blinded to group allocation. Participants will not be blinded due to the nature of the intervention.

## Interventions

After group allocation, the participants will receive an e-mail introducing them to the intervention as described below. Throughout the study there will be a project e-mail account for questions, and frequently asked questions (FAQ) will gradually be added on the website.

Safe Step - digital exercise programme

The Safe Step-application was developed by our research group in collaboration with older adults(25, 36) and the software (v2) was programmed by ICT Services and System Development, Umeå University. Participants allocated to the exercise intervention will receive instructions by e-mail on how to download the application from App Store or Google Play, as well as their personal log-in details.

On the first time use of the application, the participants view an instruction video on how to use the application to create and progress their own individual exercise programme. The participants compose their individual exercise programme choosing 10 exercises from predetermined groups of exercises with a main focus to improve balance (3 groups), increase lower-limb strength (4 groups), and improve gait/step (3 groups). The exercises are presented in video format and in increasing levels of difficulty, or with alternatives within each group of exercises. The participants are instructed to choose exercises that are challenging but not too hard. Hence, during balance exercises a feeling of being unstable but not to the extent of losing their balance and falling, and for strength exercises progress when able to easily perform the recommended repetitions. A new exercise should be selected whenever they feel that the current exercise becomes too easy or too difficult. Participants are requested to exercise for 30 minutes at least three days a week, but are also informed that a longer duration of exercising per week will give a better fall preventive effect. After each session participants will register their activities in the application: completed exercises, exercise duration, experienced well-being of the day of exercise, and experience of levels of intensity for balance and strength exercises.

In the application, the participant can make a schedule for their weekly exercise, register activity, and monitor their exercise routines (figure 2). They will also be able to set reminders tailored to their personal preferences. Direct feedback and motivational messages will be received from a virtual physiotherapist when exercise is registered. At the beginning of every month of the 1 year intervention period, the participants will receive an e-mail with a new educational video about healthy ageing and fall-prevention. The third video contains information on physical activity and exercise, but no suggestions of particular exercises are given.



For peer review only - http://bmjopen.bmj.com/site/about/guidelines.xhtml

Control group

Participants allocated to the control group will receive the same educational videos as those sent to the intervention group. After the 12-month follow-up assessment, the control group will be offered the Safe Step-application.

## Data collection

At baseline and 3, 6, 9 and 12 months respectively from the participant's inclusion in the study, an e-mail containing a link to an online questionnaire will be sent to the participants. All collected data will be self-reports, including a 30-sec chair stand self-test (30CST). In absence of completed questionnaires, a reminding e-mail will be sent out after one week. If further absence, another e-mail will be sent out after one more week with an additional question if they want to withdraw from the study. Data registered in the exercise diary of the application is collected continuously. In the same monthly e-mail as the educational videos, all participants will receive a request to report any falls during the past month by answering a short web-based questionnaire. In the absence of a completed questionnaire, one reminder will be sent after one week. All data retrieved from questionnaires, as well as application data, will be coded and reported on a group level.

## Outcomes

Primary outcome

The primary and secondary outcomes, and time points of distribution are presented in table 2. The primary outcome measure will be the rate of falls (fall per person/year) during the 12 months of the intervention. To register falls, a link to a self-report online questionnaire will be sent monthly by e-mail. To be able to report correctly the coming month, participants are asked to make notes of falls in a calendar, preferably the day the fall has occurred. They will be provided with a printable calendar in the e-mail sent out after allocation, but they can also use their own. If a fall is reported, additional questions will be asked about the cause of the fall, potential injuries sustained, need for medical attention, and hospitalization. The number of persons falling during 12 months will be analysed based on the same data as a secondary outcome.

Falls will be defined as an event in which the person comes to rest inadvertently on the ground or floor, regardless of what caused the fall or if an injury was sustained. Our definition is slightly modified and clarified in comparison with a previous consensus statement.(29) The ProFaNE definition included falls where the person come to rest on an "other lower-level", for example tripping and taking a hold of a dresser.(29) We exclude "lower-level" as our experience from previous studies is that participants seldom perceived such an event as a fall. In accordance with a systematic review,(37) we clarify that a fall should be reported regardless of cause and we also clarify that unintentionally coming to rest on the floor or ground is a fall regardless of injury. Our definition is printed in the online questionnaire.

Secondary outcomes

Socio-demographic parameters, such as age, gender, health, use of smartphones and tablets, and living conditions will be collected at baseline to understand which older adults who are reached through the intervention.

Self-reported data regarding physical assessments will be collected at baseline, 3 months, 6 months, 9 months and 12 months respectively. Single item questions with a 5- graded Likert scale are used to assess self-rated improvements in strength and balance (table 2). A 30-sec chair stand test is performed by the participants following instructions in the online questionnaire. The original



For peer review only - http://bmjopen.bmj.com/site/about/guidelines.xhtml

administration of the test has shown excellent test-retest reliability and criterion validity for assessing functional lower extremity leg strength in older adults,(38) but studies regarding the psychometric properties of a self-test is lacking. Instructions for the participants to be able to perform the 30CST by themselves at home were developed by the research group. After feedback from an advisory group of older adults, the instructions were slightly revised. When performing the test the participants are asked to place a chair against the wall and take a seat in the middle of the chair with both feet in full contact with the floor and arms folded across the chest. They are instructed to fully stand up and sit down as many times as possible for 30 seconds and register the number of full stands. Participants are asked to use the Borg Scale of Perceived Exertion(39) in combination with the 30CST test to quantify their effort. The perceived exertion is rated on a scale between 6 (none) to 20 (maximal exertion).

The Swedish version of Falls Efficacy Scale-International (FES-I(S))(40) will be used to measure concerns about falling. FES-I is widely used among older adults and is suitable for use in different populations and in clinical trials.(41) The FES-I and FES-I (S) has shown excellent internal and test-retest reliability.(40, 42)

Health-related quality of life will be included in the secondary outcomes to enable us to conduct economic analysis and will be assessed by the European Quality of Life, 5 dimensions (EQ-5D-5L)(43) self-report questionnaire. Cost-effectiveness will be conducted from a societal perspective at 12 months.

Both groups will also be asked to rate their use and perceived usefulness of the monthly delivered educational videos on 4-level ordinal scales.

Additional questions will be asked to the exercise intervention group regarding adherence, experienced positive effects besides strength and balance, and adverse events. Adherence to exercise will be evaluated by self-report questions at follow up assessments through pre-defined alternatives, and will be summarized as exercised min/week. Additionally, adherence to exercise including the proportion meeting at least the predefined minutes of 3x30 min/week, will be collected through an exercise diary integrated within the Safe Step-application.

At self-report assessments at 3, 6, 9 and 12 months after study start, a single item question with predefined answers will be used to assess experienced positive effects of the exercise intervention besides effects on balance and strength.

Adverese events in form of falls occuring while exercising with the Safe Step-application will be monitored monthly. If a participant registers a fall in the monthly questionnaire, there will be a follow-up question of whether the fall occurred while exercising with the Safe Step-programme. Additional adverse events of taking part in the intervention (eg. muscle strain) will be collected at assessments at 3, 6, 9 and 12 months after study start, and measured through a single-item question: “Have you experienced any negative effects of the exercise intervention?”. The participants will be presented with predefined alternatives and an optional text field.

At the end of the 12-month intervention, the participants in both groups will be asked if they have started to do any other exercise activities or programmes during the intervention period. In addition, self-rated improvements in balance and leg-strength over the whole course of the intervention will be measured through single item questions. Both groups will also be asked to report if they have had any extra cost related to the intervention during the intervention period (e.g. for internet access or equipment) to inform the cost-effectiveness analysis. Drop-outs and withdrawals from the study will be registered throughout the 12-month intervention. Withdrawals will be defined as those who have



For peer review only - http://bmjopen.bmj.com/site/about/guidelines.xhtml

formally discontinued the study by filling in the withdraw questionnaire. Participants that consistently discontinue to answer the questionnaires will be defined as drop-outs.

## Data storing and management

Participants will be assigned a pseudonymised identifying code (ID) when registering for the study. E-mail addresses will be collected at registration, but no names or social security numbers. Data from the online questionnaires will be directly entered into LimeSurvey software, integrated with a data collection platform, developed and hosted by ITS at Umeå University. Through LimeSurvey, several data quality features have been implemented, for example, obligatory fields and range checks.

The identity of the participants will be separated from research data entries, i.e. questionnaire response and mobile-based exercise diary entries, by storing the identity in a separate database. Access to sensitive information requires a separate set of credentials. ITS at Umeå University provides all web-applications, services and storage for the study. This is provided in accordance with research regulations and GDPR. Data entries will be monitored by one of the researchers throughout the study.

For peer review only

**Table 2.** List of primary and secondary outcome measures

| Outcome measures | BA | C | M | 3-month | 6-month | 9-month | 12-month |
|---|---|---|---|---|---|---|---|
| Fall rate[1] (fall per person/year) | | | X | | | | |
| Number of fallers[2] (n) | | | X | | | | |
| Number of falls during exercise with Safe Step[3] (n) | | | X | | | | |
| Fall-related injuries[3] (yes/no, need of medical attention, type of injury) | | | X | | | | |
| Hospitalization due to falls[3] (yes/no and number of days) | | | X | | | | |
| Reach[3] (Self-reported socio-demographic parameters such as age, sex, years of education, use of smartphone and tablet, number of cohabitants, and self-reported health and diagnosis) | X | | | | | | |
| Self-administered 30-second chair stand test[4] | X | | | X | X | X | X |
| The Borg Scale of Perceived Exertion during 30-sec chair stand test[5] (numeric scale, score 6-20) | X | | | X | X | X | X |
| Self-rated balance[5], "How do you perceive your balance?" (5-level ordinal scale) | X | | | X | X | X | X |
| Self-rated leg strength[5], "How do you perceive your leg muscle strength?" (5-level ordinal scale) | X | | | X | X | X | X |
| Fear of falling[4] (Falls Efficacy Scale-International, FES-I) | X | | | X | X | X | X |
| Health-related quality of life[4] (EQ-5D-5L) | X | | | X | X | X | X |
| Experienced positive effects of the exercise intervention[3] (nominal scale, optional text field) | X* | | | X* | X* | X* | X* |
| Experienced negative effects of the exercise intervention[3] (nominal scale, optional text field) | X* | | | X* | X* | X* | X* |
| Exercise adherence throughout the intervention reported through an integrated exercise diary in the Safe step-application[3,4] | | X* | | | | | |
| Exercise adherence, self-reported min/week[3] | | | | X* | X* | X* | X* |
| Use and perceived usefulness of educational videos[5] (4-level ordinal scale) | | | | X | X | X | X |
| Self-rated improvements in balance[6], "If you compare with when you started this study a year ago, how would you assess balance today?" (5-level ordinal scale) | | | | | | | X |
| Self-rated improvements in leg-strength[6], "If you compare with when you started this study a year ago, how would you assess the muscle strength in your legs today?" (5-level ordinal scale) | | | | | | | X |



For peer review only - http://bmjopen.bmj.com/site/about/guidelines.xhtml

| | | | | | | | |
|---|---|---|---|---|---|---|---|
| Rate of attrition[2] (n) | | | | | | | X |
| New exercise routines[3] (yes/no, text field to describe the exercise, time since initiation (predefined alternatives), minutes/week (predefined alternatives)) | | | | | | | X |
| Physical activity[4] (minutes/week, predefined alternatives) | X | | | | | | X |
| Self-reported costs related to the interventions[3] (amount and what) | | | | | | | X |
| Cost-effectiveness of the interventions with respect to falls[4] | | | | | | | X |

BA=Baseline assessment, C=Continously, M=Monthly administered, *Only administered to the exercise intervention group, [1]Negative binomial regression, [2]Logistic regression, [3]Descriptive analysis, [4]Linear mixed model, [5]Cumulative link mixed model, [6]Ordinal logistic regression

For peer review only

## Sample size

The sample size calculation for the primary outcome (fall rate over the 12 month intervention period) is based on the review of Sherrington et al.(44) by calculating for a fall rate of 1 fall/person-year. In addition, a measure of over dispersion of 1,2 is used.(21) Thus, we require 420 participants in each group to have an 80% power to detect a 20% reduction in fall rate at a 5% level of significance. We estimate that at least 1400 participants is needed based on a calculated attrition rate of 50% and a contribution of data from drop-outs. Due to uncertain drop-out rate we will include up to 2000 participants.

## Planned statistical analyses

Analyses will be performed according to the intention-to-treat principle. Thus, all available data for each randomized participant will be analyzed according to original allocation and regardless of level of participation. The primary outcome, i.e. the number of falls per person-year, will be analysed using negative binomial regression to account for an expected over-dispersion with respect to Poisson distribution.(45) The fall rate ratio of the two groups will be estimated with a 95% confidence interval and presented with a p-value while adjusting for the prognostic baseline covariates of age and gender for the purpose of increasing precision in estimates and statistical power. In addition, unadjusted results will be presented in accordance with recommendations from the CONSORT 2010 statement.(32)

For secondary outcomes on ordinal variables measured quarterly, analysis will be performed using cumulative link mixed models with logit link function and group, time point and group*timepoint interaction as fixed factors and participant as a random factor. In continuously scored secondary variables measured quarterly, general mixed linear models will be used to assess the group difference. Logistic regression models will be used to compare groups on dichotomous outcome measures.

Effects on falls, attrition and self-reported effects on balance and strength will be studied in subgroup analyses in relation to sex, different age-groups, reported health status, physical activity, and use of smartphones and tablets. Further subgroup analysis will examine exercise adherence in the exercise group as well as the dose-response relationships (min/week). Descriptive statistics will be provided to reveal the socio-demographic distribution in the sample in comparison to the population and geographical distribution in urban and rural areas.

Multiple imputation will be performed on variables containing data considered to be missing at random if proportions of missing data does not exceed 40%, as suggested in literature.(46) In addition, analysis on original unimputed data will be presented as supplement. All statistical tests will be two-sided with a significance level of 0.05.

## Economic evaluation

Results will be presented as incremental cost-effectiveness ratio (ICER) after 12 months, i.e., the ratio of the difference in costs and the difference in health effects between the intervention group versus the control group. Data on health effects will be based on the primary outcome falls averted and the secondary outcome health-related quality of life assessed by the EQ-5D-5L. The data on health-related quality of life will be used to calculate quality-adjusted life years (QALYs). Health and social care costs will be estimated by self-reported data on monthly falls and their consequences in combination with previously published data on the specific cost for the most frequent injuries. Resource use will include intervention costs, healthcare costs, and community service costs.



For peer review only - http://bmjopen.bmj.com/site/about/guidelines.xhtml

### Ethics and dissemination

Ethical approval was obtained by The Regional Ethical Review Board in Umeå (Dnr 2018/433-31). The results of the study will be reported at national and international conferences, be submitted to peer-reviewed journals, and presented to senior citizens organizations. Data from the Safe Step-RCT will be available upon reasonable request when the results have been published in peer-reviewed journals.

## DISCUSSION

To facilitate appropriate exercise to reduce age-related functional decline and prevent falls, attractive and evidence-based interventions are needed that can reach older people, including those who live rurally, or who merely want to self-manage their risk of falling. This study will evaluate the effectiveness of a completely self-managed digital exercise programme for fall prevention with additional educational videos compared to educational videos only.

The Safe Step-application is available for smartphones and tablets and provides evidence-based exercises for the home-environment. Besides conventional exercises, Safe Step inspires the integration of exercises in daily activities, both indoors and outdoors. The approach of integrating exercise into already existing daily routines has shown great potential to reduce the rate of falls among older adults.(17)

To support the older adults to sustain their exercise behaviour over time, Safe step includes a set of behaviour change strategies. The older users will create their individual exercise programme, plan their exercise and monitor their exercise routines in order to support exercise self-management. Self-management of health and exercise has been recommended to improve adherence to fall prevention programmes by strengthening the older adults' self-efficacy.(11) However, delivering exercise by mobile technology increases the complexity of providing individual support. Promisingly, in our previous feasibility study, participants expressed confidence in self-managing their exercise programmes, both in digital format and as a standard paper booklet. Worth noting is that use of the Safe Step-exercise programme seems to have supported the older adults to a greater extent than a paper booklet.(22)

Adherence to exercise based fall-prevention is often poor over time,(10) and due to the nature of this intervention with online recruitment and an intervention period of one year, an attrition of 50% is anticipated. Still, this study will assess the effectiveness of a self-managed digital exercise programme to prevent falls in real-life settings. Hence, the attrition rates will be a valuable contribution in understanding more about older adults exercise adherence when exercising with smartphones and tablets in a home-based setting.

Home-based exercise has been shown to be preferred among older adults of advancing age, those with recent falls, and those with lower socioeconomic status.(15) On one hand, fall prevention interventions delivered by digital technology might create a constraint for older adults who can not afford digital technology or do not have digital competency. On the other hand, digital technology will present opportunities for older adults that might not have the opportunity or interest in attending community based programmes or regularly visit health care centres. Additionally, in recent years, the use of digital technology such as smartphones and tablets has increased sharply among older adults. In 2019, 67% of the aged Swedish population used the internet on their smartphone, and 40% on their tablet,(47) and the rapid increase in use is expected to continue. In light of these considerations, it is important to explore reach as suggested in the present study to describe which



For peer review only - http://bmjopen.bmj.com/site/about/guidelines.xhtml

older adults that are interested in exercising with a digital application with no interaction with a health care professional.

Besides effect on falls, this research has the potential to increase knowledge regarding which older adults are interested in a digital self-management exercise intervention, exercise adherence, and effects on self-rated physical function. The design of this study with few inclusion and exclusion criteria and a national recruitment will strengthen external validity in developed countries similar to Sweden with an ageing population and large rural areas.  If proven effective and cost-effective, the Safe Step digital exercise programme has the potential to be highly accessible and reduce risk of functional decline and falls.

Authors' contributions

Original study concept was conceived by MS and LLO. MS, LLO, DS, BP and ER contributed to the design of the study. BP drafted the manuscript with contribution from all authors. MZ provided input on health economics and PL on statistics. All authors read and approved the final version of the manuscript.

Funding statement

This work was financially supported by the Swedish Research Council (grant number 2015-03481); the Strategic Research Programme in Care Sciences, Umeå University and The Karolinska Institute, Sweden; the Swedish Research Council for Health, Working Life and Welfare (FORTE); and King Gustav V's and Queen Victoria's Freemasons' Foundation.

Competing interests

The authors declare that they have no competing interest besides that DAS is a Director of Later Life Training, a not for profit training company in the UK that delivers group falls prevention exercise training (Otago and FaME) to health and fitness professionals. The App was developed collaboratively and it will not be sold for profit.

For peer review only



For peer review only - http://bmjopen.bmj.com/site/about/guidelines.xhtml

## REFERENCES


1. Tinetti ME, Speechley M, Ginter SF. Risk factors for falls among elderly persons living in the community. *N Engl J Med.* 1988;319(26):1701-7.

2. Campbell AJ, Borrie MJ, Spears GF. Risk factors for falls in a community-based prospective study of people 70 years and older. *J Gerontol.* 1989;44(4):M112-7.

3. Campbell AJ, Robertson MC. Rethinking individual and community fall prevention strategies: a meta-regression comparing single and multifactorial interventions. *Age Ageing.* 2007;36(6):656-62.

4. National Board of Health and Welfare. Statistical database: Yttre orsaker till skador och förgiftningar Stockholm: Socialstyrelsen [Accessed: 20190926]. Available from: http://www.socialstyrelsen.se/Statistik/statistikdatabas/.

5. Pohl P, Nordin E, Lundquist A, Bergstrom U, Lundin-Olsson L. Community-dwelling older people with an injurious fall are likely to sustain new injurious falls within 5 years--a prospective long-term follow-up study. *BMC Geriatr.* 2014;14:120.

6. Davis JC, Robertson MC, Ashe MC, Liu-Ambrose T, Khan KM, Marra CA. International comparison of cost of falls in older adults living in the community: a systematic review. *Osteoporosis Int.* 2010;21(8):1295-306.

7. WHO, Department of Ageing and Life Course. WHO Global Report on Falls Prevention in Older Age. Geneva: World Health Organization; 2007.

8. Finnegan S, Seers K, Bruce J. Long-term follow-up of exercise interventions aimed at preventing falls in older people living in the community: a systematic review and meta-analysis. *Physiotherapy.* 2018.

9. Sherrington C, Fairhall NJ, Wallbank GK, Tiedemann A, Michaleff ZA, Howard K, et al. Exercise for preventing falls in older people living in the community. *Cochrane Database Syst Rev.* 2019;1:CD012424.

10. Nyman SR, Victor CR. Older people's participation in and engagement with falls prevention interventions in community




For peer review only - http://bmjopen.bmj.com/site/about/guidelines.xhtml

settings: an augment to the Cochrane systematic review. *Age Ageing.* 2012;41(1):16-23.
11. Yardley L, Beyer N, Hauer K, McKee K, Ballinger C, Todd C. Recommendations for promoting the engagement of older people in activities to prevent falls. *Qual Saf Health Care.* 2007;16(3):230-4.
12. Finnegan S, Bruce J, Seers K. What enables older people to continue with their falls prevention exercises? A qualitative systematic review. *BMJ Open.* 2019;9(4):e026074.
13. Sandlund M, Pohl P, Ahlgren C, Skelton DA, Melander-Wikman A, Bergvall-Kareborn B, et al. Gender Perspective on Older People's Exercise Preferences and Motivators in the Context of Falls Prevention: A Qualitative Study. *Biomed Res Int.* 2018;2018:6865156.
14. Cramm JM, Hartgerink JM, Steyerberg EW, Bakker TJ, Mackenbach JP, Nieboer AP. Understanding older patients' self-management abilities: functional loss, self-management, and well-being. *Qual Life Res.* 2013;22(1):85-92.
15. Yardley L, Kirby S, Ben-Shlomo Y, Gilbert R, Whitehead S, Todd C. How likely are older people to take up different falls prevention activities? *Prev Med.* 2008;47(5):554-8.
16. Robertson MC, Campbell AJ, Gardner MM, Devlin N. Preventing injuries in older people by preventing falls: a meta-analysis of individual-level data. *J Am Geriatr Soc.* 2002;50(5):905-11.
17. Weber M, Belala N, Clemson L, Boulton E, Hawley-Hague H, Becker C, et al. Feasibility and Effectiveness of Intervention Programmes Integrating Functional Exercise into Daily Life of Older Adults: A Systematic Review. *Gerontology.* 2018;64(2):172-87.
18. Robinson L, Newton JL, Jones D, Dawson P. Self-management and adherence with exercise-based falls prevention programmes: a qualitative study to explore the views and experiences of older people and physiotherapists. *Disabil Rehabil.* 2014;36(5):379-86.
19. Hamm J, Money AG, Atwal A, Paraskevopoulos I. Fall prevention intervention technologies: A conceptual framework and survey of the state of the art. *J Biomed Inform.* 2016;59:319-45.
20. van Het Reve E, Silveira P, Daniel F, Casati F, de Bruin ED. Tablet-based strength-balance training to motivate and improve adherence



For peer review only - http://bmjopen.bmj.com/site/about/guidelines.xhtml

to exercise in independently living older people: part 2 of a phase II preclinical exploratory trial. *J Med Internet Res.* 2014;16(6):e159.
21. Delbaere K, Valenzuela T, Woodbury A, Davies T, Yeong J, Steffens D, et al. Evaluating the effectiveness of a home-based exercise programme delivered through a tablet computer for preventing falls in older community-dwelling people over 2 years: study protocol for the Standing Tall randomised controlled trial. *BMJ Open.* 2015;5(10):e009173.
22. Pettersson B, Wiklund M, Janols R, Lindgren H, Lundin-Olsson L, Skelton DA, et al. 'Managing pieces of a personal puzzle' - Older people's experiences of self-management falls prevention exercise guided by a digital program or a booklet. *BMC Geriatr.* 2019;19(1):43.
23. Hawley-Hague H, Boulton E, Hall A, Pfeiffer K, Todd C. Older adults' perceptions of technologies aimed at falls prevention, detection or monitoring: a systematic review. *Int J Med Inform.* 2014;83(6):416-26.
24. Lorig K, Laurent DD, Plant K, Krishnan E, Ritter PL. The components of action planning and their associations with behavior and health outcomes. *Chronic Illn.* 2014;10(1):50-9.
25. Sandlund M, Lindgren H, Pohl P, Melander-Wikman A, Bergvall-Kåreborn B, L L-O. Towards a mobile exercise application to prevent falls: a participatory design process. *Int J Child Health Hum Dev.* 2016;9(3).
26. Gardner MM, Buchner DM, Robertson MC, Campbell AJ. Practical implementation of an exercise-based falls prevention programme. *Age Ageing.* 2001;30(1):77-83.
27. Skelton D, Dinan S, Campbell M, Rutherford O. Tailored group exercise (Falls Management Exercise -- FaME) reduces falls in community-dwelling older frequent fallers (an RCT). *Age Ageing.* 2005;34(6):636-9.
28. Skelton DA, Dinan SM. Exercise for falls management: Rationale for an exercise programme aimed at reducing postural instability. *Physiother Theory Pract.* 1999;15(2):105-20.
29. Lamb SE, Jorstad-Stein EC, Hauer K, Becker C, Prevention of Falls Network E, Outcomes Consensus G. Development of a common



For peer review only - http://bmjopen.bmj.com/site/about/guidelines.xhtml

outcome data set for fall injury prevention trials: the Prevention of Falls Network Europe consensus. *J Am Geriatr Soc* 2005;53(9):1618-22.
30. Chan AW, Tetzlaff JM, Altman DG, Laupacis A, Gotzsche PC, Krleza-Jeric K, et al. SPIRIT 2013 Statement: Defining Standard Protocol Items for Clinical Trials. *Ann Intern Med.* 2013;158(3):200-+.
31. Chan AW, Tetzlaff JM, Gotzsche PC, Altman DG, Mann H, Berlin JA, et al. SPIRIT 2013 explanation and elaboration: guidance for protocols of clinical trials. *BMJ-Brit Med J.* 2013;346.
32. Schulz KF, Altman DG, Moher D, Group C. CONSORT 2010 statement: updated guidelines for reporting parallel group randomised trials. *BMJ.* 2010;340:c332.
33. Eysenbach G, Group C-E. CONSORT-EHEALTH: improving and standardizing evaluation reports of Web-based and mobile health interventions. *J Med Internet Res.* 2011;13(4):e126.
34. Husereau D, Drummond M, Petrou S, Carswell C, Moher D, Greenberg D, et al. Consolidated Health Economic Evaluation Reporting Standards (CHEERS) statement. *BMJ.* 2013;346:f1049.
35. Uschner D, Hilgers RD, Schindler D, Heussen N. randomizeR: An R Package for the Assessment and Implementation of Randomization in Clinical Trials. *J Stat Softw.* 2018;85(8):1-22.
36. Lindgren H, Lundin-Olsson L, Pohl P, Sandlund M. End users transforming experiences into formal information and process models for personalised health interventions. *Stud Health Technol Inform.* 2014;205:378-82.
37. Hauer K, Lamb SE, Jorstad EC, Todd C, Becker C, Group P. Systematic review of definitions and methods of measuring falls in randomised controlled fall prevention trials. *Age Ageing.* 2006;35(1):5-10.
38. Jones CJ, Rikli RE, Beam WC. A 30-s chair-stand test as a measure of lower body strength in community-residing older adults. *Res Q Exerc Sport.* 1999;70(2):113-9.
39. Borg GA. Psychophysical bases of perceived exertion. *Med Sci Sports Exerc.* 1982;14(5):377-81.

For peer review only



For peer review only - http://bmjopen.bmj.com/site/about/guidelines.xhtml

40. Nordell E, Andreasson M, Gall K, Thorngren K. Evaluating the Swedish version of the Falls Efficacy Scale-International (FES-I). *Adv Physiother.* 2009;11(2):81-7.
41. Kempen GI, Todd CJ, Van Haastregt JC, Zijlstra GA, Beyer N, Freiberger E, et al. Cross-cultural validation of the Falls Efficacy Scale International (FES-I) in older people: results from Germany, the Netherlands and the UK were satisfactory. *Disabil Rehabil.* 2007;29(2):155-62.
42. Yardley L, Beyer N, Hauer K, Kempen G, Piot-Ziegler C, Todd C. Development and initial validation of the Falls Efficacy Scale-International (FES-I). *Age Ageing.* 2005;34(6):614-9.
43. Herdman M, Gudex C, Lloyd A, Janssen M, Kind P, Parkin D, et al. Development and preliminary testing of the new five-level version of EQ-5D (EQ-5D-5L). *Qual Life Res.* 2011;20(10):1727-36.
44. Sherrington C, Michaleff ZA, Fairhall N, Paul SS, Tiedemann A, Whitney J, et al. Exercise to prevent falls in older adults: an updated systematic review and meta-analysis. *Br J Sports Med.* 2017;51(24):1750-8.
45. Robertson MC, Campbell AJ, Herbison P. Statistical analysis of efficacy in falls prevention trials. *J Gerontol a-Biol.* 2005;60(4):530-4.
46. Jakobsen JC, Gluud C, Wetterslev J, Winkel P. When and how should multiple imputation be used for handling missing data in randomised clinical trials - a practical guide with flowcharts. *BMC Med Res Methodol.* 2017;17.
47. Internetstiftelsen. Svenskarna och internet 2019. Available from: https://svenskarnaochinternet.se/app/uploads/2019/10/svenskarna-och-internet-2019-a4.pdf; [Accessed: Nov 2019].

For peer review only



For peer review only - http://bmjopen.bmj.com/site/about/guidelines.xhtml

**Figure 1.** Flow chart of study design.

**Figure 2.** Features of the Safe Step-application

For peer review only



For peer review only - http://bmjopen.bmj.com/site/about/guidelines.xhtml

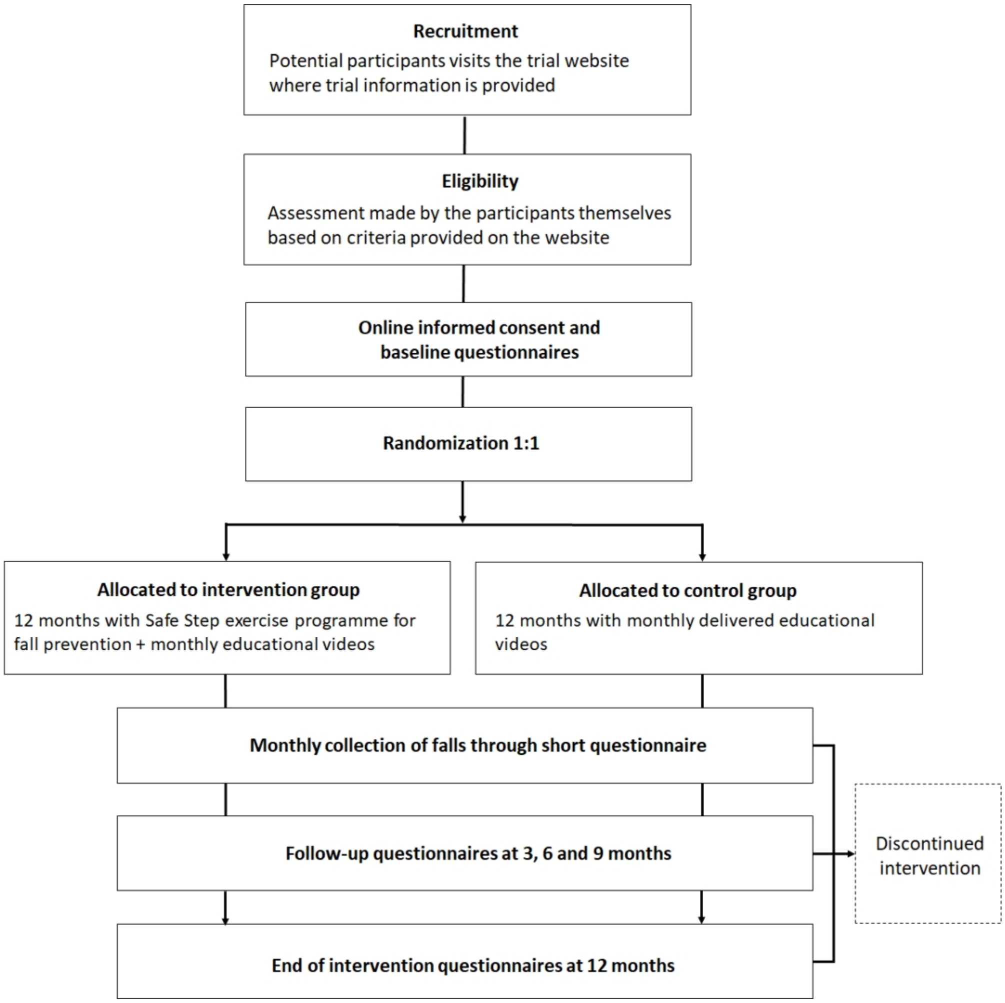


Figure 1. Flow chart of study design.

103x102mm (300 x 300 DPI)

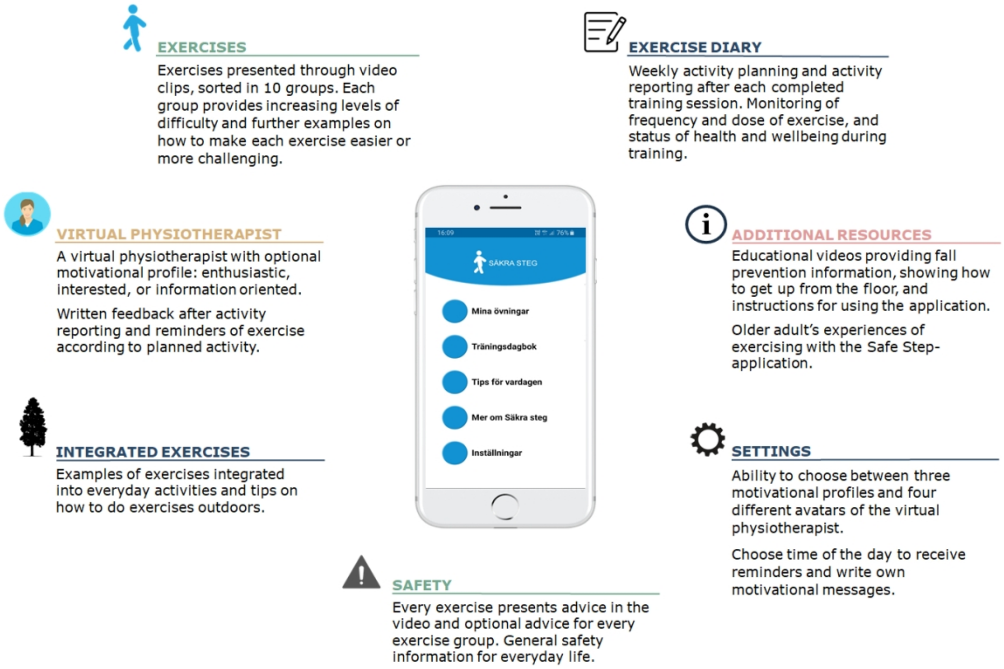


Figure 2. Features of the Safe Step-application.

115x82mm (300 x 300 DPI)

SPIRIT

STANDARD PROTOCOL ITEMS: RECOMMENDATIONS FOR INTERVENTIONAL TRIALS

SPIRIT 2013 Checklist: Recommended items to address in a clinical trial protocol and related documents*

| Section/item | Item No | Description | Addressed on page number |
|---|---|---|---|
| **Administrative information** | | | |
| Title | 1 | Descriptive title identifying the study design, population, interventions, and, if applicable, trial acronym | 1 |
| Trial registration | 2a | Trial identifier and registry name. If not yet registered, name of intended registry | Abstract page 2, and p. 4, row 148 |
| | 2b | All items from the World Health Organization Trial Registration Data Set | Yes |
| Protocol version | 3 | Date and version identifier | Final submitted version |
| Funding | 4 | Sources and types of financial, material, and other support | p. 14, row 427 |
| Roles and responsibilities | 5a | Names, affiliations, and roles of protocol contributors | p. 14, row 421 |
| | 5b | Name and contact information for the trial sponsor | p.1, Marlene Sandlund PI |
| | 5c | Role of study sponsor and funders, if any, in study design; collection, management, analysis, and interpretation of data; writing of the report; and the decision to submit the report for publication, including whether they will have ultimate authority over any of these activities | p. 14, row 421 |

For peer review only



For peer review only - http://bmjopen.bmj.com/site/about/guidelines.xhtml

| | | | |
|---|---|---|---|
| | 5d | Composition, roles, and responsibilities of the coordinating centre, steering committee, endpoint adjudication committee, data management team, and other individuals or groups overseeing the trial, if applicable (see Item 21a for data monitoring committee) | ______________ |
| **Introduction** | | | |
| Background and rationale | 6a | Description of research question and justification for undertaking the trial, including summary of relevant studies (published and unpublished) examining benefits and harms for each intervention | p. 3 |
| | 6b | Explanation for choice of comparators | p. 3 |
| Objectives | 7 | Specific objectives or hypotheses | p. 4, row 122 |
| Trial design | 8 | Description of trial design including type of trial (eg, parallel group, crossover, factorial, single group), allocation ratio, and framework (eg, superiority, equivalence, noninferiority, exploratory) | p. 4, row 119; p. 4, row 139 |
| **Methods: Participants, interventions, and outcomes** | | | |
| Study setting | 9 | Description of study settings (eg, community clinic, academic hospital) and list of countries where data will be collected. Reference to where list of study sites can be obtained | p. 5, row 150 |
| Eligibility criteria | 10 | Inclusion and exclusion criteria for participants. If applicable, eligibility criteria for study centres and individuals who will perform the interventions (eg, surgeons, psychotherapists) | Table 1 |
| Interventions | 11a | Interventions for each group with sufficient detail to allow replication, including how and when they will be administered | p. 6, row 186 |
| | 11b | Criteria for discontinuing or modifying allocated interventions for a given trial participant (eg, drug dose change in response to harms, participant request, or improving/worsening disease) | p. 7, row 224; p. 8, row 305 |
| | 11c | Strategies to improve adherence to intervention protocols, and any procedures for monitoring adherence (eg, drug tablet return, laboratory tests) | p. 6, row 210; p. 7, row 224; p. 8, row 284 |



For peer review only - http://bmjopen.bmj.com/site/about/guidelines.xhtml

| | | | |
|---|---|---|---|
| | 11d | Relevant concomitant care and interventions that are permitted or prohibited during the trial | _____________ |
| Outcomes | 12 | Primary, secondary, and other outcomes, including the specific measurement variable (eg, systolic blood pressure), analysis metric (eg, change from baseline, final value, time to event), method of aggregation (eg, median, proportion), and time point for each outcome. Explanation of the clinical relevance of chosen efficacy and harm outcomes is strongly recommended | p. , row 235 |
| Participant timeline | 13 | Time schedule of enrolment, interventions (including any run-ins and washouts), assessments, and visits for participants. A schematic diagram is highly recommended (see Figure) | Figure 1 |
| Sample size | 14 | Estimated number of participants needed to achieve study objectives and how it was determined, including clinical and statistical assumptions supporting any sample size calculations | p. 12, row 326 |
| Recruitment | 15 | Strategies for achieving adequate participant enrolment to reach target sample size | p. 5, row 156 |

**Methods: Assignment of interventions (for controlled trials)**

Allocation:

| | | | |
|---|---|---|---|
| Sequence generation | 16a | Method of generating the allocation sequence (eg, computer-generated random numbers), and list of any factors for stratification. To reduce predictability of a random sequence, details of any planned restriction (eg, blocking) should be provided in a separate document that is unavailable to those who enrol participants or assign interventions | p. 12, row 326 |
| Allocation concealment mechanism | 16b | Mechanism of implementing the allocation sequence (eg, central telephone; sequentially numbered, opaque, sealed envelopes), describing any steps to conceal the sequence until interventions are assigned | p. 12, row 326 |
| Implementation | 16c | Who will generate the allocation sequence, who will enrol participants, and who will assign participants to interventions | p. 12, row 326 |
| Blinding (masking) | 17a | Who will be blinded after assignment to interventions (eg, trial participants, care providers, outcome assessors, data analysts), and how | p. 12, row 176 |
| | 17b | If blinded, circumstances under which unblinding is permissible, and procedure for revealing a participant's allocated intervention during the trial | _____________ |

**Methods: Data collection, management, and analysis**

| | | | |
|---|---|---|---|
| Data collection methods | 18a | Plans for assessment and collection of outcome, baseline, and other trial data, including any related processes to promote data quality (eg, duplicate measurements, training of assessors) and a description of study instruments (eg, questionnaires, laboratory tests) along with their reliability and validity, if known. Reference to where data collection forms can be found, if not in the protocol | p.7, row 224; p.7. 235; p. 9, row 320; table 2 |
| | 18b | Plans to promote participant retention and complete follow-up, including list of any outcome data to be collected for participants who discontinue or deviate from intervention protocols | p. 5, row 165; p. 7 row 227 |
| Data management | 19 | Plans for data entry, coding, security, and storage, including any related processes to promote data quality (eg, double data entry; range checks for data values). Reference to where details of data management procedures can be found, if not in the protocol | p. 9, row 309 |
| Statistical methods | 20a | Statistical methods for analysing primary and secondary outcomes. Reference to where other details of the statistical analysis plan can be found, if not in the protocol | p. 12, row 334; p. 12, row 360; table 2 |
| | 20b | Methods for any additional analyses (eg, subgroup and adjusted analyses) | p. 12, row 350 |
| | 20c | Definition of analysis population relating to protocol non-adherence (eg, as randomised analysis), and any statistical methods to handle missing data (eg, multiple imputation) | p. 12, row 335; p 12, row 356 |

**Methods: Monitoring**

| | | | |
|---|---|---|---|
| Data monitoring | 21a | Composition of data monitoring committee (DMC); summary of its role and reporting structure; statement of whether it is independent from the sponsor and competing interests; and reference to where further details about its charter can be found, if not in the protocol. Alternatively, an explanation of why a DMC is not needed | _____________ |
| | 21b | Description of any interim analyses and stopping guidelines, including who will have access to these interim results and make the final decision to terminate the trial | p. 5, row 177; p. 8, row 293 |
| Harms | 22 | Plans for collecting, assessing, reporting, and managing solicited and spontaneously reported adverse events and other unintended effects of trial interventions or trial conduct | p. 8, row 293 |



For peer review only - http://bmjopen.bmj.com/site/about/guidelines.xhtml

| | | | |
|---|---|---|---|
| Auditing | 23 | Frequency and procedures for auditing trial conduct, if any, and whether the process will be independent from investigators and the sponsor | _______________ |
| **Ethics and dissemination** | | | |
| Research ethics approval | 24 | Plans for seeking research ethics committee/institutional review board (REC/IRB) approval | Abstract; p. 13, row 370 |
| Protocol amendments | 25 | Plans for communicating important protocol modifications (eg, changes to eligibility criteria, outcomes, analyses) to relevant parties (eg, investigators, REC/IRBs, trial participants, trial registries, journals, regulators) | _______________ |
| Consent or assent | 26a | Who will obtain informed consent or assent from potential trial participants or authorised surrogates, and how (see Item 32) | p. 5, row 162 |
| | 26b | Additional consent provisions for collection and use of participant data and biological specimens in ancillary studies, if applicable | _______________ |
| Confidentiality | 27 | How personal information about potential and enrolled participants will be collected, shared, and maintained in order to protect confidentiality before, during, and after the trial | p. 9, row 309 |
| Declaration of interests | 28 | Financial and other competing interests for principal investigators for the overall trial and each study site | p. 14, row 433 |
| Access to data | 29 | Statement of who will have access to the final trial dataset, and disclosure of contractual agreements that limit such access for investigators | p. 13, row 369 |
| Ancillary and post-trial care | 30 | Provisions, if any, for ancillary and post-trial care, and for compensation to those who suffer harm from trial participation | _______________ |
| Dissemination policy | 31a | Plans for investigators and sponsor to communicate trial results to participants, healthcare professionals, the public, and other relevant groups (eg, via publication, reporting in results databases, or other data sharing arrangements), including any publication restrictions | p. 13, row 369 |
| | 31b | Authorship eligibility guidelines and any intended use of professional writers | _______________ |
| | 31c | Plans, if any, for granting public access to the full protocol, participant-level dataset, and statistical code | p. 13, row 369 |

For peer review

**Appendices**

| | | | |
|---|---|---|---|
| Informed consent materials | 32 | Model consent form and other related documentation given to participants and authorised surrogates | ______________ |
| Biological specimens | 33 | Plans for collection, laboratory evaluation, and storage of biological specimens for genetic or molecular analysis in the current trial and for future use in ancillary studies, if applicable | ______________ |

*It is strongly recommended that this checklist be read in conjunction with the SPIRIT 2013 Explanation & Elaboration for important clarification on the items. Amendments to the protocol should be tracked and dated. The SPIRIT checklist is copyrighted by the SPIRIT Group under the Creative Commons "Attribution-NonCommercial-NoDerivs 3.0 Unported" license.

For peer review only

BMJ Open

# Effectiveness of a self-managed digital exercise programme to prevent falls in older community-dwelling adults –study protocol for the Safe Step randomized controlled trial

| | |
|---|---|
| Journal: | *BMJ Open* |
| Manuscript ID | bmjopen-2019-036194.R1 |
| Article Type: | Protocol |
| Date Submitted by the Author: | 11-Mar-2020 |
| Complete List of Authors: | Pettersson, Beatrice; Umeå University, Department of Community Medicine and Rehabilitation, Physiotherapy<br>Lundin-Olsson, Lillemor; Umeå University, Department of Community Medicine and Rehabilitation, Physiotherapy<br>Skelton, Dawn; Glasgow Caledonian University, School of Health & Life Sciences<br>Liv, Per; Umeå University, Department of Public Health and Clinical Medicine, Section of Sustainable Health<br>Zingmark, Magnus ; Municipality of Östersund, Health and Social Care Administration; Umeå University, Department of Epidemiology and Global Health<br>Rosendahl, Erik; Umeå University, Department of Community Medicine and Rehabilitation, Physiotherapy<br>Sandlund, Marlene; Umeå University, Department of Community Medicine and Rehabilitation, Physiotherapy |
| <b>Primary Subject Heading</b>: | Public health |
| Secondary Subject Heading: | Geriatric medicine |
| Keywords: | PREVENTIVE MEDICINE, STATISTICS & RESEARCH METHODS, PUBLIC HEALTH, GERIATRIC MEDICINE |
| | |

SCHOLARONE™
Manuscripts

*I, the Submitting Author has the right to grant and does grant on behalf of all authors of the Work (as defined in the below author licence), an exclusive licence and/or a non-exclusive licence for contributions from authors who are: i) UK Crown employees; ii) where BMJ has agreed a CC-BY licence shall apply, and/or iii) in accordance with the terms applicable for US Federal Government officers or employees acting as part of their official duties; on a worldwide, perpetual, irrevocable, royalty-free basis to BMJ Publishing Group Ltd ("BMJ") its licensees and where the relevant Journal is co-owned by BMJ to the co-owners of the Journal, to publish the Work in this journal and any other BMJ products and to exploit all rights, as set out in our licence.*

*The Submitting Author accepts and understands that any supply made under these terms is made by BMJ to the Submitting Author unless you are acting as an employee on behalf of your employer or a postgraduate student of an affiliated institution which is paying any applicable article publishing charge ("APC") for Open Access articles. Where the Submitting Author wishes to make the Work available on an Open Access basis (and intends to pay the relevant APC), the terms of reuse of such Open Access shall be governed by a Creative Commons licence – details of these licences and which Creative Commons licence will apply to this Work are set out in our licence referred to above.*

*Other than as permitted in any relevant BMJ Author's Self Archiving Policies, I confirm this Work has not been accepted for publication elsewhere, is not being considered for publication elsewhere and does not duplicate material already published. I confirm all authors consent to publication of this Work and authorise the granting of this licence.*

For peer review only

# Title

Effectiveness of a self-managed digital exercise programme to prevent falls in older community-dwelling adults –study protocol for the Safe Step randomized controlled trial

# Authors

**Beatrice Pettersson**, Department of Community Medicine and Rehabilitation, Physiotherapy, Umeå University, Umeå, Sweden. beatrice.pettersson@umu.se **(corresponding author)**

**Lillemor Lundin-Olsson**, Department of Community Medicine and Rehabilitation, Physiotherapy, Umeå University, Umeå, Sweden. lillemor.lundin-olsson@umu.se

**Dawn A Skelton**, School of Health and Life Sciences, Glasgow Caledonian University, Glasgow, UK. dawn.skelton@gcu.ac.uk

**Per Liv**, Department of Public Health and Clinical Medicine, Section of Sustainable Health, Umeå University, Umeå, Sweden. per.liv@umu.se

**Magnus Zingmark**, [a]Municipality of Östersund, Health and Social Care Administration, Östersund, Sweden, [b]Department of Epidemiology and Global Health, Umeå University, Sweden. magnus.zingmark@ostersund.se

**Erik Rosendahl**, Department of Community Medicine and Rehabilitation, Physiotherapy, Umeå University, Umeå, Sweden. erik.rosendahl@umu.se

**Marlene Sandlund**, Department of Community Medicine and Rehabilitation, Physiotherapy, Umeå University, Umeå, Sweden. marlene.sandlund@umu.se

**Word count: 4603**



For peer review only - http://bmjopen.bmj.com/site/about/guidelines.xhtml

# ABSTRACT

## Introduction

Exercise interventions have a strong evidence base for falls prevention. However, exercise can be challenging to implement and often has limited reach and poor adherence. Digital technology provides opportunities for both increased access to the intervention and support over time. Further knowledge needs to be gained regarding the effectiveness of completely self-managed digital exercise interventions. The main objective of this study is to compare the effectiveness of a self-managed digital exercise programme, Safe Step, in combination with monthly educational videos with educational videos alone, on falls over one year in older community-dwelling adults.

## Methods and analysis

A two-arm parallel randomized controlled trial will be conducted with at least 1400 community-living older adults (70+ years) who experience impaired balance. Participants will be recruited throughout Sweden with enrolment through the project website. They will be randomly allocated to either the Safe Step exercise programme with additional monthly educational videos about healthy ageing and fall prevention, or the monthly education videos alone. Participants receiving the exercise intervention will be asked to exercise at home for at least 30 minutes, 3 times/week with support of the Safe Step-application. The primary outcome will be rate of falls (fall per person-year). Participants will keep a fall calendar and report falls at the end of each month through a digital questionnaire. Further assessments of secondary outcomes will be made through self-reported questionnaires and a self-test of 30-sec chair stand test at baseline and 3, 6, 9 and 12 months after study start. Data will be analysed according to the intention-to-treat principle.

## Ethics and dissemination

Ethical approval was obtained by The Regional Ethical Review Board in Umeå (Dnr 2018/433-31). Findings will be disseminated through the project web-site, peer-reviewed journals, national and international conferences, and through senior citizen organisations' newsletters.

## Trial registration number

NCT03963570


Bullet points, stenghts and limitations of the study:

- The method of using a completely self-managed digital exercise program for delivering fall prevention exercise is novel and will contribute to the evidence of using smartphones and tablets for delivering fall prevention exercise.
- The Safe Step-exercise program includes behaviour change support to facilitate maintenance of the new exercise routine.
- The use of wide inclusion criteria could enhance external validity.
- A limitation of the study is that large attrition rates are expected due to the study design of no face-to-face interaction, and there is a risk of unequal distribution between groups.
- To assess intervention reach, the socio-demographic parameters of participants will be compared to the target population.



For peer review only

For peer review only - http://bmjopen.bmj.com/site/about/guidelines.xhtml

# BACKGROUND

About one-third of community-living older adults fall every year(1, 2), with an increasing frequency with a higher age and frailty level.(3) In Sweden, falls represent 87% of injury-related hospital admissions in older adults(4), with a 30% risk of another fall which requires medical attention in the next five years.(5) Globally, the related health care costs are substantial(6), and is likely to exponentially increase with the growing proportion of older people.(7)

There is strong evidence that falls among community-living older adults can be prevented both short- and long term using exercise as a single intervention.(8, 9) The most effective types of exercise are balance and functional exercises combined with resistance exercises.(9) Even though there is a large body of evidence that supports the effectiveness of exercise interventions, up to 52% of older adults engaging in individually targeted fall prevention exercise do not adhere to the exercise over 12 months.(10)

To aid older adults in changing their exercise behaviour, there is a need for tailored exercise interventions that are not only adapted to the individual's physical function but also promote personal benefits, such as increased or prolonged independence.(11, 12) Furthermore, interventions need to increase motivation for exercise by taking into consideration the older adults' individual lifestyle, perceived risks of exercise, as well as physical capabilities.(13) To support self-efficacy for exercise, older adults can be engaged in creating and tailoring their own routines, which has been suggested as a route to improved adherence.(11) Additionally, support of older adults' active roles are preferable to having complete dependence on professionals,(7) and can also improve well-being among older adults.(14)

Home-based exercise provides a practical choice for participants and could solve issues with both accessibility and costs as well as support the older person to self-manage their exercise. According to a large survey, home-based exercises were preferred over other fall prevention strategies, especially in individuals of an older age, those who have recently fallen, or those who are socially disadvantaged.(15) To exercise at home with occasional visits from instructors has been shown to be effective in preventing falls(16) as well as to integrate the exercise into everyday activities at home(17), which many older adults also prefer.(18)

The use of technology and digital health are emerging as ways of delivering self-managed exercise and fall prevention interventions for older adults.(19-21) Such digital interventions enable the participants to exercise at preferred times and locations.(22, 23) Technology can further enable the use of tools for behaviour change such as self-monitoring, direct feed-back, and action planning, which can support self-efficacy for self-management.(24) Digital technology can, therefore, provide support over time, which is important due to high attrition rates from fall prevention exercise programmes.(10)

There is limited evidence of the effectiveness of smartphones and tablets for delivering fall prevention exercise.(19) Of those evaluated, the interventions has been combined with the interaction of health professionals, e.g.(20, 21). If proven effective, completely self-managed digital interventions with no interaction with a health professional would provide a more rapid and extensive implementation of fall prevention programmes, vastly increasing reach to a wider population. Additionally, it may be economically beneficial due to reduced use of resources from health care professionals. In this emerging field, more research is needed regarding the effectiveness of digital interventions with no interaction between health care professionals and participants.



For peer review only - http://bmjopen.bmj.com/site/about/guidelines.xhtml

We have previously developed a completely self-managed exercise application for smartphones and tablets with a focus on improving physical function and preventing falls, the Safe Step-application.(25) The application provides a large repository of evidence-based exercises in short video formats with associated safety advice. Behaviour change is supported through strengthening of self-efficacy and behaviour change techniques incorporated in the application. The participants are supported to create their individual programme, plan their weekly exercise and monitor their exercise routine in the application. To further support behaviour change, the participants receive motivational feedback and reminders of their planned exercise from a virtual physiotherapist. The exercise programme is influenced by the Otago home exercise programme that has been proven effective in reducing falls among community-living older adults,(26, 27) but also expanded with both easier and more challenging exercises mainly inspired by the Falls Management Exercise (FaME) programme.(28) The Safe Step-application (v1) was explored in a feasibility study, a participant preference trial comparing the programme to a paper booklet with exercises. Results from a qualitative study showed that the participant using the digital exercise programme found it both easy to use and easy to tailor to their own circumstances.(22) The majority of the participants exercised at least during 75% of the intervention weeks and one third of the participants completed more than 75% of recommended exercise time. (Mansson L, Lundin-Olsson L, Skelton D, Janols R, Lindgren H, Rosendahl E, Sandlund M 2020, revised and re-submitted).

To investigate the effectiveness of a self-managed digital exercise programme, this randomised (1:1) controlled trial (RCT) aims to compare the Safe Step-application in combination with monthly educational videos with a control group that only receives the educational videos.

The primary aim is to evaluate the effect on fall rate in older community-dwelling adults during a 12-month intervention period. Secondary aims are to evaluate number of fallers, self-reported balance and strength, fear of falling, quality of life, cost-effectiveness, and attrition and exercise adherence. In addition, we will describe socio-demographic parameters of those who are recruited in comparison to the target population in order to assess intervention reach.

# METHOD

## Participant and Public Involvement

The Safe Step-application has been designed and co-created with, and for older adults.(25) In further co-creation sessions, behaviour change components were developed together with older adults to enhance long-term adherence. The feasibility and acceptability of outcome measures have been informed by a feasibility trial. The ProFaNE consensus of a common data set for fall injury prevention trials(29) and a feasibility study have informed the choice of outcome measures in this RCT. In addition, a small reference group has tested the final questionnaires and provided feedback. A spokesperson for senior citizen organisations in Sweden was consulted regarding recruitment for the RCT-study.

## Study design

The 1-year Safe Step RCT-study uses a two-arm parallel design with digital interventions. The intervention group receives the Safe Step-application, delivered on smartphones or tablets. Both groups will receive an e-mail every month with short educational videos about healthy ageing and falls prevention.

This protocol is reported in line with the Standard Protocol Items: Recommendations for Interventional Trials (SPIRIT) guidelines.(30, 31) The RCT will be reported according to the Consolidated Standards of Reporting Trials (CONSORT) statement,(32) and CONSORT EHEALTH



For peer review only - http://bmjopen.bmj.com/site/about/guidelines.xhtml

criteria.(33) The health economic evaluation will be reported according to the Consolidated Health Economic Evaluation Reporting Standards (CHEERS) statement.(34) The trial was registered prior to study start at ClinicalTrials.gov (NCT03963570).

## Participants

At least 1400 and up to 2000 older adults living in the community will be recruited throughout Sweden. The inclusion and exclusion criteria are shown in table 1.

**Table 1.** Inclusion and exclusion criteria

| Inclusion criteria | Exclusion criteria |
|---|---|
| • Older adults 70 years or older<br>• Fallen or experienced a decline in perceived postural balance during the last year<br>• Have access to a smartphone or tablet and uses it regularly<br>• Have an own e-mail address and uses it<br>• Able to understand verbal and written instructions in Swedish<br>• Can rise from a standard height chair without a person helping<br>• Can walk independently without a walking aid indoors | • Progressive disease where there is likely to be a decline in strength or balance over the next year<br>• Perceived memory dysfunction that affect everyday life activities<br>• Taking part in more than 3 hours each week of strenuous physical exercise which makes them out of breath (e.g. dance, gymnastics, gym exercises, running, or skiing) |

## Recruitment

To reach the target sample size, at least one year is estimated for enrolment of participants starting in Sept 2019. The recruitment strategies will be directed towards the largest senior citizen organisations with the potential to reach approximately 800 000 members, and information will be distributed via the Physiotherapist union, section for older adult's health. Recruitment strategies will include both digital and non-digital strategies. The digital recruitment strategies include advertisements on webpages and Facebook; posts on open and closed Facebook-pages; e-mails or digital newsletters directly to members of the senior citizen organizations. Non-digital strategies include advertisements in newspapers and in Swedish magazines commonly read by older adults. Participants will be directed to the open-access project website (www.sakrasteg.se) where the necessary study information is provided to decide on participation. A self-assessment of eligibility will be performed by the participant based on the inclusion and exclusion criteria provided (table 1). On the project website, participants are informed that by providing their e-mail address they will give their informed consent for participation and consent for publication of their pseudonymised data. An e-mail will be sent to them to confirm their registration and verify their e-mail address, including one reminder. When confirmation has been received a link will be sent to the first baseline questionnaire (figure 1).



For peer review only - http://bmjopen.bmj.com/site/about/guidelines.xhtml

## Randomization and Blinding

After completed baseline questionnaire, the participants will be randomized in a 1:1 ratio using Permuted Block Randomization with randomly varied block sizes. A statistician not involved in the study will generate the allocation list using the randomizeR R-package before enrolment.(35) A computer programme developed in FLOW, Microsoft Office 365-software, will randomize participants according to the allocation list.

After randomization, the FLOW programme will automatically send e-mails with follow-up questionnaires as well as the monthly fall reports and educational videos. In order to include participants, monitor data collection, and answer e-mails regarding technical issues, two members of the research group will be able to see the participant's e-mail addresses and group allocation in the FLOW-system and database. However, no personal contact will occur between researchers and participants regarding the results of their outcome assessments.

Analyses of the outcomes regarding falls and physical function will be made by a researcher blinded to group allocation. Participants will not be blinded due to the nature of the intervention.

## Interventions

After group allocation, the participants will receive an e-mail introducing them to the interventions as described below. Throughout the study there will be a project e-mail account for questions, and frequently asked questions (FAQ) may gradually be added on the website.

Safe Step - digital exercise programme

The Safe Step-application was developed by our research group in collaboration with older adults(25, 36) and the software (v2) was programmed by ICT Services and System Development, Umeå University. Participants allocated to the exercise intervention will receive instructions by e-mail on how to download the application from App Store or Google Play, as well as their personal log-in details.

On the first time use of the application, the participants view an instruction video on how to use the application to create and progress their own individual exercise programme. The participants compose their individual exercise programme choosing 10 exercises from predetermined groups of exercises with a main focus to improve balance (3 groups), increase lower-limb strength (4 groups), and improve gait/step (3 groups). The exercises are presented in video format and in increasing levels of difficulty, or with alternatives within each group of exercises. The participants are instructed to choose exercises that are challenging but not too hard. Hence, during balance exercises a feeling of being unstable but not to the extent of losing their balance and falling, and for strength exercises progress when able to easily perform the recommended repetitions. A new exercise should be selected whenever they feel that the current exercise becomes too easy or too difficult. Participants are requested to exercise for 30 minutes at least three days per week, but are also informed that a longer duration of exercising per week will give a better fall preventive effect. After each session participants will register their activities in the application: completed exercises, exercise duration, experienced well-being of the day of exercise, and experience of levels of intensity for balance and strength exercises.

In the application, the participant can make a schedule for their weekly exercise, register activity, and monitor their exercise routines (figure 2). They will also be able to set reminders tailored to their personal preferences. Direct feedback and motivational messages will be received from a virtual



For peer review only - http://bmjopen.bmj.com/site/about/guidelines.xhtml

physiotherapist when exercise is registered. At the beginning of every month of the 1 year intervention period, the participants will receive an e-mail with a new educational video about healthy ageing and fall-prevention. The third video contains information on physical activity and exercise, but no suggestions of particular exercises are given.

Control group

Participants allocated to the control group will receive the same educational videos as those sent to the intervention group. After the 12-month follow-up assessment, the control group will be offered the Safe Step-application.

## Data collection

At baseline and 3, 6, 9 and 12 months respectively from the participant's inclusion in the study, an e-mail containing a link to an online questionnaire will be sent to the participants. All collected data will be self-reports, including a 30-sec chair stand self-test (30CST). In absence of completed questionnaires, a reminding e-mail will be sent out after one week. If further absence, another e-mail will be sent after an additional week. This reminder will also include a question if they want to withdraw from the study. Data registered in the exercise diary of the application is collected continuously. In the same monthly e-mail as the educational videos, all participants will receive a request to report any falls during the past month by answering a short web-based questionnaire. In the absence of a completed questionnaire, one reminder will be sent after one week. All data retrieved from questionnaires, as well as application data, will be coded and reported on a group level.

## Outcomes

Primary outcome

The primary and secondary outcomes, and time points of distribution are presented in table 2. The primary outcome measure will be the rate of falls (fall per person/year) during the 12 months of the intervention. To register falls, a link to a self-report online questionnaire will be sent monthly by e-mail. To be able to report correctly the coming month, participants are asked to make notes of falls in a calendar, preferably the day the fall has occurred. They will be provided with a printable calendar in the e-mail sent out after allocation, but they can also use their own. If a fall is reported, additional questions will be asked about the cause of the fall, potential injuries sustained, need for medical attention, and hospitalization. The number of persons falling during 12 months will be analysed based on the same data as a secondary outcome.

Falls will be defined as an event in which the person comes to rest inadvertently on the ground or floor, regardless of what caused the fall or if an injury was sustained. Our definition is slightly modified and clarified in comparison with a previous consensus statement.(29) The ProFaNE definition included falls where the person come to rest on an "other lower-level", for example tripping and taking a hold of a dresser.(29) We exclude "lower-level" as our experience from previous studies is that participants seldom perceived such an event as a fall. In accordance with a systematic review,(37) we clarify that a fall should be reported regardless of cause and we also clarify that unintentionally coming to rest on the floor or ground is a fall regardless of injury. Our definition is printed in the online questionnaire.

Secondary outcomes



For peer review only - http://bmjopen.bmj.com/site/about/guidelines.xhtml

Socio-demographic parameters, such as age, sex, health, use of smartphones and tablets, and living conditions will be collected at baseline to understand which older adults who are reached through the intervention and to be able to assess predictors of completion.

Self-reported data regarding physical assessments will be collected at baseline, 3 months, 6 months, 9 months and 12 months respectively. Single item questions with a 5- graded Likert scale are used to assess self-rated improvements in strength and balance (table 2). A 30-sec chair stand test is performed by the participants following instructions in the online questionnaire. The original administration of the test has shown excellent test-retest reliability and criterion validity for assessing functional lower extremity leg strength in older adults,(38) but studies regarding the psychometric properties of a self-test is lacking. Instructions for the participants to be able to perform the 30CST by themselves at home were developed by the research group. After feedback from an advisory group of older adults, the instructions were slightly revised. When performing the test, the participants are asked to place a chair against the wall and take a seat in the middle of the chair with both feet in full contact with the floor and arms folded across the chest. They are instructed to fully stand up and sit down as many times as possible for 30 seconds and register the number of full stands. Participants are asked to use the Borg Scale of Perceived Exertion(39) in combination with the 30CST test to quantify their effort. The perceived exertion is rated on a scale between 6 (none) to 20 (maximal exertion).

The Swedish version of Falls Efficacy Scale-International (FES-I(S))(40) will be used to measure concerns about falling. FES-I is widely used among older adults and is suitable for use in different populations and in clinical trials.(41) The FES-I and FES-I (S) has shown excellent internal and test-retest reliability.(40, 42)

Health-related quality of life will be included in the secondary outcomes to enable us to conduct economic analysis and will be assessed by the European Quality of Life, 5 dimensions (EQ-5D-5L)(43) self-report questionnaire. Cost-effectiveness will be conducted from a societal perspective at 12 months.

Both groups will also be asked to rate their use and perceived usefulness of the monthly delivered educational videos on 4-level ordinal scales.

Additional questions will be asked to the exercise intervention group regarding adherence, experienced positive effects besides strength and balance, and adverse events. Adherence to exercise will be evaluated by self-report questions at follow up assessments through pre-defined alternatives, and will be summarized as exercised min/week. Additionally, adherence to exercise including the proportion meeting at least the predefined minutes of 3x30 min/week, will be collected through an exercise diary integrated within the Safe Step-application.

At self-report assessments at 3, 6, 9 and 12 months after study start, a single item question with predefined answers will be used to assess experienced positive effects of the exercise intervention besides effects on balance and strength.

Adverse events in form of falls occurring while exercising with the Safe Step-application will be monitored monthly. If a participant registers a fall in the monthly questionnaire, there will be a follow-up question of whether the fall occurred while exercising with the Safe Step-programme. If so, a member of the research team will contact the participant to ask him or her about the nature of the fall, and if necessary, suggest measures to prevent further falls during exercise. Additional adverse events of taking part in the intervention (eg. muscle strain) will be collected at assessments at 3, 6, 9 and 12 months after study start, and measured through a single-item question: “Have you



For peer review only - http://bmjopen.bmj.com/site/about/guidelines.xhtml

experienced any negative effects of the exercise intervention?". The participants will be presented with predefined alternatives and an optional text field.

At the end of the 12-month intervention, the participants in both groups will be asked if they have started to do any other exercise activities or programmes during the intervention period. In addition, self-rated improvements in balance and leg-strength over the whole course of the intervention will be measured through single item questions. Both groups will also be asked to report if they have had any extra cost related to the intervention during the intervention period (e.g. for internet access or equipment) to inform the cost-effectiveness analysis. Drop-outs and withdrawals from the study will be registered throughout the 12-month intervention. Withdrawals will be defined as those who have formally discontinued the study by filling in the withdraw questionnaire. Participants that consistently discontinue to answer the questionnaires will be defined as drop-outs.

## Data storing and management

Participants will be assigned a pseudonymised identifying code (ID) when registering for the study. E-mail addresses will be collected at registration, but no names or social security numbers. Data from the online questionnaires will be directly entered into LimeSurvey software, integrated with a data collection platform, developed and hosted by ITS at Umeå University. Through LimeSurvey, several data quality features have been implemented, for example, obligatory fields and range checks.

The identity of the participants will be separated from research data entries, i.e. questionnaire response and mobile-based exercise diary entries, by storing the identity in a separate database. Access to sensitive information requires a separate set of credentials. ITS at Umeå University provides all web-applications, services and storage for the study. This is provided in accordance with research regulations and GDPR. Data entries will be monitored by one of the researchers throughout the study.



For peer review only - http://bmjopen.bmj.com/site/about/guidelines.xhtml

**Table 2.** List of primary and secondary outcome measures

| Outcome measures | BA | C | M | 3-month | 6-month | 9-month | 12-month |
|---|---|---|---|---|---|---|---|
| Fall rate[1] (fall per person/year) | | | X | | | | |
| Number of fallers[2] (n) | | | X | | | | |
| Number of falls during exercise with Safe Step[3] (n) | | | X | | | | |
| Fall-related injuries[3] (yes/no, need of medical attention, type of injury) | | | X | | | | |
| Hospitalization due to falls[3] (yes/no and number of days) | | | X | | | | |
| Reach[3] (Self-reported socio-demographic parameters such as age, sex, years of education, use of smartphone and tablet, number of cohabitants, and self-reported health and diagnosis) | X | | | | | | |
| Self-administered 30-second chair stand test[4] | X | | | X | X | X | X |
| The Borg Scale of Perceived Exertion during 30-sec chair stand test[5] (numeric scale, score 6-20) | X | | | X | X | X | X |
| Self-rated balance[5], “How do you perceive your balance?” (5-level ordinal scale) | X | | | X | X | X | X |
| Self-rated leg strength[5], “How do you perceive your leg muscle strength?” (5-level ordinal scale) | X | | | X | X | X | X |
| Fear of falling[4] (Falls Efficacy Scale-International, FES-I) | X | | | X | X | X | X |
| Health-related quality of life[4] (EQ-5D-5L) | X | | | X | X | X | X |
| Experienced positive effects of the exercise intervention[3] (nominal scale, optional text field) | X* | | | X* | X* | X* | X* |
| Experienced negative effects of the exercise intervention[3] (nominal scale, optional text field) | X* | | | X* | X* | X* | X* |
| Exercise adherence throughout the intervention reported through an integrated exercise diary in the Safe step-application[3,4] | | X* | | | | | |
| Exercise adherence, self-reported min/week[3] | | | | X* | X* | X* | X* |
| Use and perceived usefulness of educational videos[5] (4-level ordinal scale) | | | | X | X | X | X |
| Self-rated improvements in balance[6], "If you compare with when you started this study a year ago, how would you assess balance today?" (5-level ordinal scale) | | | | | | | X |
| Self-rated improvements in leg-strength[6], "If you compare with when you started this study a year ago, how would you assess the muscle strength in your legs today?" (5-level ordinal scale) | | | | | | | X |



For peer review only - http://bmjopen.bmj.com/site/about/guidelines.xhtml

| | | | | | | | |
|---|---|---|---|---|---|---|---|
| Rate of attrition[2] (n) | | | | | | | X |
| New exercise routines[3] (yes/no, text field to describe the exercise, time since initiation (predefined alternatives), minutes/week (predefined alternatives)) | | | | | | | X |
| Physical activity[4] (minutes/week, predefined alternatives) | X | | | | | | X |
| Self-reported costs related to the interventions[3] (amount and what) | | | | | | | X |
| Cost-effectiveness of the interventions with respect to falls[4] | | | | | | | X |

BA=Baseline assessment, C= Continuously, M=Monthly administered, *Only administered to the exercise intervention group, [1]Negative binomial regression, [2]Logistic regression, [3]Descriptive analysis, [4]Linear mixed model, [5]Cumulative link mixed model, [6]Ordinal logistic regression

For peer review only



For peer review only - http://bmjopen.bmj.com/site/about/guidelines.xhtml

## Sample size

The sample size calculation for the primary outcome (fall rate over the 12-month intervention period) is based on the review of Sherrington et al.(44) by calculating for a fall rate of 1 fall/person-year. In addition, a measure of over dispersion of 1,2 is used.(21) Thus, we require 420 participants in each group to have an 80% power to detect a 20% reduction in fall rate at a 5% level of significance. We estimate that at least 1400 participants are needed based on a calculated attrition rate of 50% and a contribution of data from drop-outs. Due to uncertain drop-out rate we will include up to 2000 participants.

## Planned statistical analyses

Analyses will be performed according to the intention-to-treat principle. Thus, all available data for each randomized participant will be analysed  according to original allocation and regardless of level of participation. The primary outcome, i.e. the number of falls per person-year, will be analysed using negative binomial regression to account for an expected over-dispersion with respect to Poisson distribution.(45) The fall rate ratio of the two groups will be estimated with a 95% confidence interval and presented with a p-value while adjusting for the prognostic baseline covariates of age and sex for the purpose of increasing precision in estimates and statistical power. In addition, unadjusted results will be presented in accordance with recommendations from the CONSORT 2010 statement.(32)

For secondary outcomes on ordinal variables measured quarterly, analysis will be performed using cumulative link mixed models with logit link function and group, time point and group*timepoint interaction as fixed factors and participant as a random factor. In continuously scored secondary variables measured quarterly, general mixed linear models will be used to assess the group difference. Logistic regression models will be used to compare groups on dichotomous outcome measures.

Effects on falls, attrition and self-reported effects on balance and strength will be studied in subgroup analyses in relation to sex, different age-groups, reported health status, physical activity, and use of smartphones and tablets.  Further subgroup analysis will examine exercise adherence in the exercise group as well as the dose-response relationships (min/week). Descriptive statistics will be provided to reveal the socio-demographic distribution in the sample in comparison to the population and geographical distribution in urban and rural areas.

Multiple imputation will be performed on variables containing data considered to be missing at random if proportions of missing data does not exceed 40%, as suggested in literature.(46) In addition, analysis on original unimputed data will be presented as supplement. All statistical tests will be two-sided with a significance level of 0.05.

## Economic evaluation

Results will be presented as incremental cost-effectiveness ratio (ICER) after 12 months, i.e., the ratio of the difference in costs and the difference in health effects between the intervention group versus the control group. Data on health effects will be based on the primary outcome falls averted and the secondary outcome health-related quality of life assessed by the EQ-5D-5L. The data on health-related quality of life will be used to calculate quality-adjusted life years (QALYs). Health and social care costs will be estimated by self-reported data on monthly falls and their consequences in combination with previously published data on the specific cost for the most frequent injuries. Resource use will include intervention costs, healthcare costs, and community service costs.



For peer review only - http://bmjopen.bmj.com/site/about/guidelines.xhtml

## Ethics and dissemination

Ethical approval was obtained by The Regional Ethical Review Board in Umeå (Dnr 2018/433-31). The results of the study will be made publicly available through the project web-site, reported at national and international conferences, as well as in peer-reviewed journals, and presented to senior citizen organisations through member journals and digital newsletters. Data from the Safe Step-RCT will be available upon reasonable request when the results have been published in peer-reviewed journals.

# DISCUSSION

To facilitate appropriate exercise to reduce age-related functional decline and prevent falls, attractive and evidence-based interventions are needed that can reach older people, including those who live rurally, or who merely want to self-manage their risk of falling. This study will evaluate the effectiveness of a completely self-managed digital exercise programme for fall prevention with additional educational videos compared to educational videos only.

The Safe Step-application is available for smartphones and tablets and provides evidence-based exercises for the home-environment. Besides conventional exercises, Safe Step inspires the integration of exercises in daily activities, both indoors and outdoors. The approach of integrating exercise into already existing daily routines has shown great potential to reduce the rate of falls among older adults.(17)

To support the older adults to sustain their exercise behaviour over time, Safe step includes a set of behaviour change strategies. The older users will create their individual exercise programme, plan their exercise and monitor their exercise routines in order to support exercise self-management. Self-management of health and exercise has been recommended to improve adherence to fall prevention programmes by strengthening the older adults' self-efficacy.(11) However, delivering exercise by mobile technology increases the complexity of providing individual support. Promisingly, in our previous feasibility study, participants expressed confidence in self-managing their exercise programmes, both in digital format and as a standard paper booklet. Worth noting is that use of the Safe Step-exercise programme seems to have supported the older adults to a greater extent than a paper booklet.(22)

Adherence to individual exercise based fall-prevention is often poor over time.(10) A contributing factor could be a lack of social components and peer support, which has been shown to positively affect attendance and adherence to exercise classes among older adults.(47) However, preferences are diverse among older adults, and some older adults have stated to prefer home-based exercises.(15) Due to the nature of this home-based intervention with online recruitment and an intervention period of one year, an attrition of 50% is anticipated. Still, this study will assess the effectiveness of a self-managed digital exercise programme to prevent falls in real-life settings where the older adults themselves will take the initiative to download and use an application. Hence, the attrition rates will be a valuable contribution in understanding more about older adult's exercise adherence when exercising with smartphones and tablets in a home-based setting.

Home-based exercise has been shown to be preferred among older adults of advancing age, those with recent falls, and those with lower socioeconomic status.(15) On one hand, fall prevention interventions delivered by digital technology might create a constraint for older adults who cannot afford digital technology or do not have digital competency. On the other hand, digital technology will present opportunities for older adults that might not have the opportunity or interest in attending community-based programmes or regularly visit health care centres. Additionally, in recent years, the use of digital technology such as smartphones and tablets has increased sharply among older adults. In 2019, 67% of the aged Swedish population used the internet on their smartphone,



For peer review only - http://bmjopen.bmj.com/site/about/guidelines.xhtml

and 40% on their tablet,(48) and the rapid increase in use is expected to continue. In light of these considerations, it is important to explore reach as suggested in the present study to describe which older adults that are interested in exercising with a digital application with no interaction with a health care professional.

Besides effect on falls, this research has the potential to increase knowledge regarding which older adults are interested in a digital self-management exercise intervention, exercise adherence, and effects on self-rated physical function. The design of this study with few inclusion and exclusion criteria and a national recruitment will strengthen external validity in developed countries similar to Sweden with an ageing population and large rural areas. If proven effective and cost-effective, the Safe Step digital exercise programme has the potential to be highly accessible and reduce risk of functional decline and falls.

Authors' contributions

Original study concept was conceived by MS and LLO. MS, LLO, DS, BP and ER contributed to the design of the study. BP drafted the manuscript with contribution from all authors. MZ provided input on health economics and PL on statistics. All authors read and approved the final version of the manuscript.

Funding statement

This work was financially supported by the Swedish Research Council (grant number 2015-03481); the Strategic Research Programme in Care Sciences, Umeå University and The Karolinska Institute, Sweden; the Swedish Research Council for Health, Working Life and Welfare (FORTE); and King Gustav V's and Queen Victoria's Freemasons' Foundation.

Competing interests

The authors declare that they have no competing interest besides that DAS is a Director of Later Life Training, a not for profit training company in the UK that delivers group falls prevention exercise training (Otago and FaME) to health and fitness professionals. The App was developed collaboratively and it will not be sold for profit.

For peer review only

## REFERENCES

1. Tinetti ME, Speechley M, Ginter SF. Risk factors for falls among elderly persons living in the community. *N Engl J Med*. 1988;319(26):1701-7.

2. Campbell AJ, Borrie MJ, Spears GF. Risk factors for falls in a community-based prospective study of people 70 years and older. *J Gerontol.* 1989;44(4):M112-7.

3. Campbell AJ, Robertson MC. Rethinking individual and community fall prevention strategies: a meta-regression comparing single and multifactorial interventions. *Age Ageing.* 2007;36(6):656-62.

4. National Board of Health and Welfare. Statistical database: Yttre orsaker till skador och förgiftningar. Stockholm: Socialstyrelsen [20190926]. Available from: http://www.socialstyrelsen.se/Statistik/statistikdatabas/.

5. Pohl P, Nordin E, Lundquist A, Bergstrom U, Lundin-Olsson L. Community-dwelling older people with an injurious fall are likely to sustain new injurious falls within 5 years--a prospective long-term follow-up study. *BMC Geriatr.* 2014;14:120.

6. Davis JC, Robertson MC, Ashe MC, Liu-Ambrose T, Khan KM, Marra CA. International comparison of cost of falls in older adults living in the community: a systematic review. *Osteoporosis Int.* 2010;21(8):1295-306.



For peer review only - http://bmjopen.bmj.com/site/about/guidelines.xhtml

7. WHO, Department of Ageing and Life Course. WHO Global Report on Falls Prevention in Older Age. Geneva: World Health Organization; 2007.

8. Finnegan S, Seers K, Bruce J. Long-term follow-up of exercise interventions aimed at preventing falls in older people living in the community: a systematic review and meta-analysis. *Physiotherapy.* 2019;105(2):187-199.

9. Sherrington C, Fairhall NJ, Wallbank GK, Tiedemann A, Michaleff ZA, Howard K, et al. Exercise for preventing falls in older people living in the community. *Cochrane Database Syst Rev.* 2019;1:CD012424.

10. Nyman SR, Victor CR. Older people's participation in and engagement with falls prevention interventions in community settings: an augment to the Cochrane systematic review. *Age Ageing.* 2012;41(1):16-23.

11. Yardley L, Beyer N, Hauer K, McKee K, Ballinger C, Todd C. Recommendations for promoting the engagement of older people in activities to prevent falls. *Qual Saf Health Care.* 2007;16(3):230-4.

12. Finnegan S, Bruce J, Seers K. What enables older people to continue with their falls prevention exercises? A qualitative systematic review. *BMJ Open.* 2019;9(4):e026074.

13. Sandlund M, Pohl P, Ahlgren C, Skelton DA, Melander-Wikman A, Bergvall-Kareborn B, et al. Gender Perspective on Older People's Exercise Preferences and

For peer review only



For peer review only - http://bmjopen.bmj.com/site/about/guidelines.xhtml

Motivators in the Context of Falls Prevention: A Qualitative Study. *Biomed Res Int.* 2018;2018:6865156.

14. Cramm JM, Hartgerink JM, Steyerberg EW, Bakker TJ, Mackenbach JP, Nieboer AP. Understanding older patients' self-management abilities: functional loss, self-management, and well-being. *Qual Life Res.* 2013;22(1):85-92.

15. Yardley L, Kirby S, Ben-Shlomo Y, Gilbert R, Whitehead S, Todd C. How likely are older people to take up different falls prevention activities? *Prev Med.* 2008;47(5):554-8.

16. Robertson MC, Campbell AJ, Gardner MM, Devlin N. Preventing injuries in older people by preventing falls: a meta-analysis of individual-level data. *J Am Geriatr Soc.* 2002;50(5):905-11.

17. Weber M, Belala N, Clemson L, Boulton E, Hawley-Hague H, Becker C, et al. Feasibility and Effectiveness of Intervention Programmes Integrating Functional Exercise into Daily Life of Older Adults: A Systematic Review. *Gerontology.* 2018;64(2):172-87.

18. Robinson L, Newton JL, Jones D, Dawson P. Self-management and adherence with exercise-based falls prevention programmes: a qualitative study to explore the views and experiences of older people and physiotherapists. *Disabil Rehabil.* 2014;36(5):379-86.

19. Hamm J, Money AG, Atwal A, Paraskevopoulos I. Fall prevention intervention technologies: A conceptual framework

For peer review only



For peer review only - http://bmjopen.bmj.com/site/about/guidelines.xhtml

and survey of the state of the art. *J Biomed Inform.* 2016;59:319-45.

20. van Het Reve E, Silveira P, Daniel F, Casati F, de Bruin ED. Tablet-based strength-balance training to motivate and improve adherence to exercise in independently living older people: part 2 of a phase II preclinical exploratory trial. *J Med Internet Res.* 2014;16(6):e159.

21. Delbaere K, Valenzuela T, Woodbury A, Davies T, Yeong J, Steffens D, et al. Evaluating the effectiveness of a home-based exercise programme delivered through a tablet computer for preventing falls in older community-dwelling people over 2 years: study protocol for the Standing Tall randomised controlled trial. *BMJ Open.* 2015;5(10):e009173.

22. Pettersson B, Wiklund M, Janols R, Lindgren H, Lundin-Olsson L, Skelton DA, et al. 'Managing pieces of a personal puzzle' - Older people's experiences of self-management falls prevention exercise guided by a digital program or a booklet. *BMC Geriatr.* 2019;19(1):43.

23. Hawley-Hague H, Boulton E, Hall A, Pfeiffer K, Todd C. Older adults' perceptions of technologies aimed at falls prevention, detection or monitoring: a systematic review. *Int J Med Inform.* 2014;83(6):416-26.

24. Lorig K, Laurent DD, Plant K, Krishnan E, Ritter PL. The components of action planning and their associations with behavior and health outcomes. *Chronic Illn.* 2014;10(1):50-9.



For peer review only - http://bmjopen.bmj.com/site/about/guidelines.xhtml

25. Sandlund M, Lindgren H, Pohl P, Melander-Wikman A, Bergvall-Kåreborn B, L L-O. Towards a mobile exercise application to prevent falls: a participatory design process. *Int J Child Health Hum Dev*. 2016;9(3).

26. Gardner MM, Buchner DM, Robertson MC, Campbell AJ. Practical implementation of an exercise-based falls prevention programme. *Age Ageing.* 2001;30(1):77-83.

27. Skelton D, Dinan S, Campbell M, Rutherford O. Tailored group exercise (Falls Management Exercise -- FaME) reduces falls in community-dwelling older frequent fallers (an RCT). *Age Ageing.* 2005;34(6):636-9.

28. Skelton DA, Dinan SM. Exercise for falls management: Rationale for an exercise programme aimed at reducing postural instability. *Physiother Theory Pract.* 1999;15(2):105-20.

29. Lamb SE, Jorstad-Stein EC, Hauer K, Becker C, Prevention of Falls Network E, Outcomes Consensus G. Development of a common outcome data set for fall injury prevention trials: the Prevention of Falls Network Europe consensus. *J Am Geriatr Soc.* 2005;53(9):1618-22.

30. Chan AW, Tetzlaff JM, Altman DG, Laupacis A, Gotzsche PC, Krleza-Jeric K, et al. SPIRIT 2013 Statement: Defining Standard Protocol Items for Clinical Trials. *Ann Intern Med.* 2013;158(3):200-+.

31. Chan AW, Tetzlaff JM, Gotzsche PC, Altman DG, Mann H, Berlin JA, et al. SPIRIT 2013 explanation and



For peer review only - http://bmjopen.bmj.com/site/about/guidelines.xhtml

elaboration: guidance for protocols of clinical trials. *BMJ.* 2013;346:e7586.

32. Schulz KF, Altman DG, Moher D, Group C. CONSORT 2010 statement: updated guidelines for reporting parallel group randomised trials. *BMJ.* 2010;340:c332.

33. Eysenbach G, Group C-E. CONSORT-EHEALTH: improving and standardizing evaluation reports of Web-based and mobile health interventions. *J Med Internet Res.* 2011;13(4):e126.

34. Husereau D, Drummond M, Petrou S, Carswell C, Moher D, Greenberg D, et al. Consolidated Health Economic Evaluation Reporting Standards (CHEERS) statement. *BMJ.* 2013;346:f1049.

35. Uschner D, Hilgers RD, Schindler D, Heussen N. randomizeR: An R Package for the Assessment and Implementation of Randomization in Clinical Trials. *J Stat Softw.* 2018;85(8):1-22.

36. Lindgren H, Lundin-Olsson L, Pohl P, Sandlund M. End users transforming experiences into formal information and process models for personalised health interventions. *Stud Health Technol Inform.* 2014;205:378-82.

37. Hauer K, Lamb SE, Jorstad EC, Todd C, Becker C, Group P. Systematic review of definitions and methods of measuring falls in randomised controlled fall prevention trials. *Age Ageing.* 2006;35(1):5-10.

For peer review only

38. Jones CJ, Rikli RE, Beam WC. A 30-s chair-stand test as a measure of lower body strength in community-residing older adults. *Res Q Exerc Sport.* 1999;70(2):113-9.

39. Borg GA. Psychophysical bases of perceived exertion. *Med Sci Sports Exerc.* 1982;14(5):377-81.

40. Nordell E, Andreasson M, Gall K, Thorngren K. Evaluating the Swedish version of the Falls Efficacy Scale-International (FES-I). *Adv Physiother.* 2009;11(2):81-7.

41. Kempen GI, Todd CJ, Van Haastregt JC, Zijlstra GA, Beyer N, Freiberger E, et al. Cross-cultural validation of the Falls Efficacy Scale International (FES-I) in older people: results from Germany, the Netherlands and the UK were satisfactory. *Disabil Rehabil.* 2007;29(2):155-62.

42. Yardley L, Beyer N, Hauer K, Kempen G, Piot-Ziegler C, Todd C. Development and initial validation of the Falls Efficacy Scale-International (FES-I). *Age Ageing.* 2005;34(6):614-9.

43. Herdman M, Gudex C, Lloyd A, Janssen M, Kind P, Parkin D, et al. Development and preliminary testing of the new five-level version of EQ-5D (EQ-5D-5L). *Qual Life Res.* 2011;20(10):1727-36.

44. Sherrington C, Michaleff ZA, Fairhall N, Paul SS, Tiedemann A, Whitney J, et al. Exercise to prevent falls in older adults: an updated systematic review and meta-analysis. *Br J Sports Med.* 2017;51(24):1750-8.



For peer review only - http://bmjopen.bmj.com/site/about/guidelines.xhtml

45. Robertson MC, Campbell AJ, Herbison P. Statistical analysis of efficacy in falls prevention trials. *J Gerontol a-Biol.* 2005;60(4):530-4.

46. Jakobsen JC, Gluud C, Wetterslev J, Winkel P. When and how should multiple imputation be used for handling missing data in randomised clinical trials - a practical guide with flowcharts. *BMC Med Res Methodol.* 2017;17.

47. Hawley-Hague H, Horne M, Campbell M, Demack S, Skelton DA, Todd C. Multiple levels of influence on older adults' attendance and adherence to community exercise classes. *Gerontologist.* 2014;54(4):599-610.

48. Internetstiftelsen. Svenskarna och internet 2019. Available from: https://svenskarnaochinternet.se/app/uploads/2019/10/svenskarna-och-internet-2019-a4.pdf; [Accessed: Nov 2019].

**Figure 1.** Flow chart of study design.

**Figure 2.** Features of the Safe Step-application



For peer review only - http://bmjopen.bmj.com/site/about/guidelines.xhtml

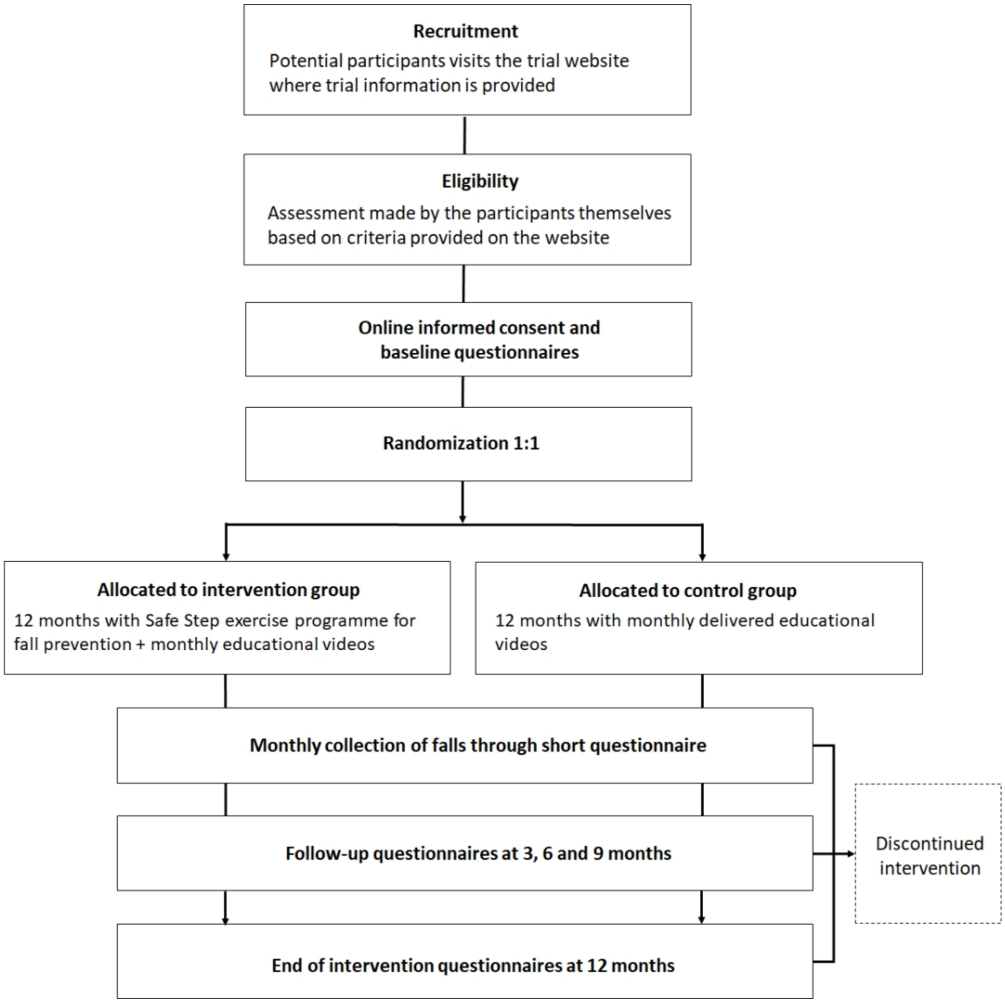


Figure 1. Flow chart of study design.

103x102mm (300 x 300 DPI)

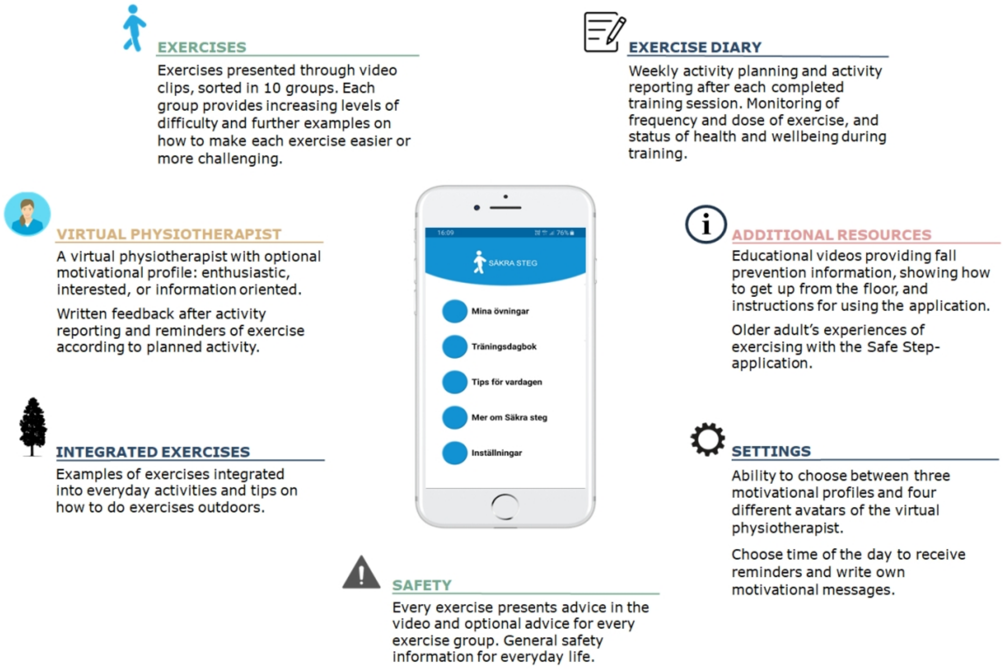


Figure 2. Features of the Safe Step-application.

115x82mm (300 x 300 DPI)

SPIRIT 2013 Checklist: Recommended items to address in a clinical trial protocol and related documents*

| Section/item | Item No | Description | Addressed on page number |
|---|---|---|---|
| **Administrative information** | | | |
| Title | 1 | Descriptive title identifying the study design, population, interventions, and, if applicable, trial acronym | 1 |
| Trial registration | 2a | Trial identifier and registry name. If not yet registered, name of intended registry | Abstract page 2, and p. 5, line 152-153 |
| | 2b | All items from the World Health Organization Trial Registration Data Set | Yes |
| Protocol version | 3 | Date and version identifier | Re-submitted manuscript |
| Funding | 4 | Sources and types of financial, material, and other support | p. 14, line 441-445 |
| Roles and responsibilities | 5a | Names, affiliations, and roles of protocol contributors | p. 14, line 435-439 |
| | 5b | Name and contact information for the trial sponsor | p.1, Marlene Sandlund PI |
| | 5c | Role of study sponsor and funders, if any, in study design; collection, management, analysis, and interpretation of data; writing of the report; and the decision to submit the report for publication, including whether they will have ultimate authority over any of these activities | p. 14, line 441-445 |


For peer review only - http://bmjopen.bmj.com/site/about/guidelines.xhtml

| | | | |
|---|---|---|---|
| | 5d | Composition, roles, and responsibilities of the coordinating centre, steering committee, endpoint adjudication committee, data management team, and other individuals or groups overseeing the trial, if applicable (see Item 21a for data monitoring committee) | ________ |
| **Introduction** | | | |
| Background and rationale | 6a | Description of research question and justification for undertaking the trial, including summary of relevant studies (published and unpublished) examining benefits and harms for each intervention | p. 3 |
| | 6b | Explanation for choice of comparators | p. 3 |
| Objectives | 7 | Specific objectives or hypotheses | p. 4, line 127-131 |
| Trial design | 8 | Description of trial design including type of trial (eg, parallel group, crossover, factorial, single group), allocation ratio, and framework (eg, superiority, equivalence, noninferiority, exploratory) | p. 4, line 124-126; p. 4, line 144-147 |
| **Methods: Participants, interventions, and outcomes** | | | |
| Study setting | 9 | Description of study settings (eg, community clinic, academic hospital) and list of countries where data will be collected. Reference to where list of study sites can be obtained | p. 5, line 155-157 |
| Eligibility criteria | 10 | Inclusion and exclusion criteria for participants. If applicable, eligibility criteria for study centres and individuals who will perform the interventions (eg, surgeons, psychotherapists) | Table 1 |
| Interventions | 11a | Interventions for each group with sufficient detail to allow replication, including how and when they will be administered | p. 7, line 193-229 |
| | 11b | Criteria for discontinuing or modifying allocated interventions for a given trial participant (eg, drug dose change in response to harms, participant request, or improving/worsening disease) | p. 7, line 233-236; p. 8, line 302-304 |
| | 11c | Strategies to improve adherence to intervention protocols, and any procedures for monitoring adherence (eg, drug tablet return, laboratory tests) | p. 6, line 218-221; p. 7, line 233-236; p. 8, line 291-296 |



For peer review only - http://bmjopen.bmj.com/site/about/guidelines.xhtml

| | | | |
|---|---|---|---|
| | 11d | Relevant concomitant care and interventions that are permitted or prohibited during the trial | _____________ |
| Outcomes | 12 | Primary, secondary, and other outcomes, including the specific measurement variable (eg, systolic blood pressure), analysis metric (eg, change from baseline, final value, time to event), method of aggregation (eg, median, proportion), and time point for each outcome. Explanation of the clinical relevance of chosen efficacy and harm outcomes is strongly recommended | p. 7, line 242-317 |
| Participant timeline | 13 | Time schedule of enrolment, interventions (including any run-ins and washouts), assessments, and visits for participants. A schematic diagram is highly recommended (see Figure) | Figure 1 |
| Sample size | 14 | Estimated number of participants needed to achieve study objectives and how it was determined, including clinical and statistical assumptions supporting any sample size calculations | p. 12, line 335-342 |
| Recruitment | 15 | Strategies for achieving adequate participant enrolment to reach target sample size | p. 5, line 161-176 |

**Methods: Assignment of interventions (for controlled trials)**

Allocation:

| | | | |
|---|---|---|---|
| Sequence generation | 16a | Method of generating the allocation sequence (eg, computer-generated random numbers), and list of any factors for stratification. To reduce predictability of a random sequence, details of any planned restriction (eg, blocking) should be provided in a separate document that is unavailable to those who enrol participants or assign interventions | p. 6, line 178-183 |
| Allocation concealment mechanism | 16b | Mechanism of implementing the allocation sequence (eg, central telephone; sequentially numbered, opaque, sealed envelopes), describing any steps to conceal the sequence until interventions are assigned | p. 6, line 178-183 |
| Implementation | 16c | Who will generate the allocation sequence, who will enrol participants, and who will assign participants to interventions | p. 6, line 178-185 |
| Blinding (masking) | 17a | Who will be blinded after assignment to interventions (eg, trial participants, care providers, outcome assessors, data analysts), and how | p. 6, line 185-191 |
| | 17b | If blinded, circumstances under which unblinding is permissible, and procedure for revealing a participant's allocated intervention during the trial | _____________ |

**Methods: Data collection, management, and analysis**

| | | | |
|---|---|---|---|
| Data collection methods | 18a | Plans for assessment and collection of outcome, baseline, and other trial data, including any related processes to promote data quality (eg, duplicate measurements, training of assessors) and a description of study instruments (eg, questionnaires, laboratory tests) along with their reliability and validity, if known. Reference to where data collection forms can be found, if not in the protocol | p.7, line 230-241; p.7. line 242; p. 9, line 322-223; table 2 |
| | 18b | Plans to promote participant retention and complete follow-up, including list of any outcome data to be collected for participants who discontinue or deviate from intervention protocols | p. 5, line 174-175; p. 7 line 230-241 |
| Data management | 19 | Plans for data entry, coding, security, and storage, including any related processes to promote data quality (eg, double data entry; range checks for data values). Reference to where details of data management procedures can be found, if not in the protocol | p. 9, line 318-329 |
| Statistical methods | 20a | Statistical methods for analysing primary and secondary outcomes. Reference to where other details of the statistical analysis plan can be found, if not in the protocol | p. 12, line 343-367; p. 12, line 368-376; table 2 |
| | 20b | Methods for any additional analyses (eg, subgroup and adjusted analyses) | p. 12, line 358-363 |
| | 20c | Definition of analysis population relating to protocol non-adherence (eg, as randomised analysis), and any statistical methods to handle missing data (eg, multiple imputation) | p. 12, line 344 and line 364-367 |

**Methods: Monitoring**

| | | | |
|---|---|---|---|
| Data monitoring | 21a | Composition of data monitoring committee (DMC); summary of its role and reporting structure; statement of whether it is independent from the sponsor and competing interests; and reference to where further details about its charter can be found, if not in the protocol. Alternatively, an explanation of why a DMC is not needed | ______________ |
| | 21b | Description of any interim analyses and stopping guidelines, including who will have access to these interim results and make the final decision to terminate the trial | p. 6, line 185-189; p. 8, line 300-308 |
| Harms | 22 | Plans for collecting, assessing, reporting, and managing solicited and spontaneously reported adverse events and other unintended effects of trial interventions or trial conduct | p. 8, line 300-308 |



For peer review only - http://bmjopen.bmj.com/site/about/guidelines.xhtml

| | | | |
|---|---|---|---|
| Auditing | 23 | Frequency and procedures for auditing trial conduct, if any, and whether the process will be independent from investigators and the sponsor | ______________ |
| **Ethics and dissemination** | | | |
| Research ethics approval | 24 | Plans for seeking research ethics committee/institutional review board (REC/IRB) approval | Abstract page 2 and p. 13, line 378 |
| Protocol amendments | 25 | Plans for communicating important protocol modifications (eg, changes to eligibility criteria, outcomes, analyses) to relevant parties (eg, investigators, REC/IRBs, trial participants, trial registries, journals, regulators) | ______________ |
| Consent or assent | 26a | Who will obtain informed consent or assent from potential trial participants or authorised surrogates, and how (see Item 32) | p. 5, line 161-176 |
| | 26b | Additional consent provisions for collection and use of participant data and biological specimens in ancillary studies, if applicable | ______________ |
| Confidentiality | 27 | How personal information about potential and enrolled participants will be collected, shared, and maintained in order to protect confidentiality before, during, and after the trial | p. 9, line 318-329 |
| Declaration of interests | 28 | Financial and other competing interests for principal investigators for the overall trial and each study site | p. 14, line 447-451 |
| Access to data | 29 | Statement of who will have access to the final trial dataset, and disclosure of contractual agreements that limit such access for investigators | p. 13, line 381-383 |
| Ancillary and post-trial care | 30 | Provisions, if any, for ancillary and post-trial care, and for compensation to those who suffer harm from trial participation | ______________ |
| Dissemination policy | 31a | Plans for investigators and sponsor to communicate trial results to participants, healthcare professionals, the public, and other relevant groups (eg, via publication, reporting in results databases, or other data sharing arrangements), including any publication restrictions | p. 13, line 378-381 |
| | 31b | Authorship eligibility guidelines and any intended use of professional writers | ______________ |
| | 31c | Plans, if any, for granting public access to the full protocol, participant-level dataset, and statistical code | p. 13, line 381-383 |

For peer review

**Appendices**

| | | | |
|---|---|---|---|
| Informed consent materials | 32 | Model consent form and other related documentation given to participants and authorised surrogates | ______________ |
| Biological specimens | 33 | Plans for collection, laboratory evaluation, and storage of biological specimens for genetic or molecular analysis in the current trial and for future use in ancillary studies, if applicable | ______________ |

*It is strongly recommended that this checklist be read in conjunction with the SPIRIT 2013 Explanation & Elaboration for important clarification on the items. Amendments to the protocol should be tracked and dated. The SPIRIT checklist is copyrighted by the SPIRIT Group under the Creative Commons "Attribution-NonCommercial-NoDerivs 3.0 Unported" license.

For peer review only

BMJ Open

# Effectiveness of a self-managed digital exercise programme to prevent falls in older community-dwelling adults –study protocol for the Safe Step randomized controlled trial



| | |
|---|---|
| Journal: | *BMJ Open* |
| Manuscript ID | bmjopen-2019-036194.R2 |
| Article Type: | Protocol |
| Date Submitted by the Author: | 14-Apr-2020 |




| | |
|---|---|
| Complete List of Authors: | Pettersson, Beatrice; Umeå University, Department of Community Medicine and Rehabilitation, Physiotherapy<br>Lundin-Olsson, Lillemor; Umeå University, Department of Community Medicine and Rehabilitation, Physiotherapy<br>Skelton, Dawn; Glasgow Caledonian University, School of Health & Life Sciences<br>Liv, Per; Umeå University, Department of Public Health and Clinical Medicine, Section of Sustainable Health<br>Zingmark, Magnus ; Municipality of Östersund, Health and Social Care Administration; Umeå University, Department of Epidemiology and Global Health<br>Rosendahl, Erik; Umeå University, Department of Community Medicine and Rehabilitation, Physiotherapy<br>Sandlund, Marlene; Umeå University, Department of Community Medicine and Rehabilitation, Physiotherapy |




| | |
|---|---|
| <b>Primary Subject Heading</b>: | Public health |
| Secondary Subject Heading: | Geriatric medicine |
| Keywords: | PREVENTIVE MEDICINE, STATISTICS & RESEARCH METHODS, PUBLIC HEALTH, GERIATRIC MEDICINE |
| | |


SCHOLARONE™
Manuscripts

*I, the Submitting Author has the right to grant and does grant on behalf of all authors of the Work (as defined in the below author licence), an exclusive licence and/or a non-exclusive licence for contributions from authors who are: i) UK Crown employees; ii) where BMJ has agreed a CC-BY licence shall apply, and/or iii) in accordance with the terms applicable for US Federal Government officers or employees acting as part of their official duties; on a worldwide, perpetual, irrevocable, royalty-free basis to BMJ Publishing Group Ltd ("BMJ") its licensees and where the relevant Journal is co-owned by BMJ to the co-owners of the Journal, to publish the Work in this journal and any other BMJ products and to exploit all rights, as set out in our licence.*

*The Submitting Author accepts and understands that any supply made under these terms is made by BMJ to the Submitting Author unless you are acting as an employee on behalf of your employer or a postgraduate student of an affiliated institution which is paying any applicable article publishing charge ("APC") for Open Access articles. Where the Submitting Author wishes to make the Work available on an Open Access basis (and intends to pay the relevant APC), the terms of reuse of such Open Access shall be governed by a Creative Commons licence – details of these licences and which Creative Commons licence will apply to this Work are set out in our licence referred to above.*

*Other than as permitted in any relevant BMJ Author's Self Archiving Policies, I confirm this Work has not been accepted for publication elsewhere, is not being considered for publication elsewhere and does not duplicate material already published. I confirm all authors consent to publication of this Work and authorise the granting of this licence.*

For peer review only

# Title

Effectiveness of a self-managed digital exercise programme to prevent falls in older community-dwelling adults –study protocol for the Safe Step randomized controlled trial

# Authors

**Beatrice Pettersson**, Department of Community Medicine and Rehabilitation, Physiotherapy, Umeå University, Umeå, Sweden. beatrice.pettersson@umu.se **(corresponding author)**

**Lillemor Lundin-Olsson**, Department of Community Medicine and Rehabilitation, Physiotherapy, Umeå University, Umeå, Sweden. lillemor.lundin-olsson@umu.se

**Dawn A Skelton**, School of Health and Life Sciences, Glasgow Caledonian University, Glasgow, UK. dawn.skelton@gcu.ac.uk

**Per Liv**, Department of Public Health and Clinical Medicine, Section of Sustainable Health, Umeå University, Umeå, Sweden. per.liv@umu.se

**Magnus Zingmark**, [a]Municipality of Östersund, Health and Social Care Administration, Östersund, Sweden, [b]Department of Epidemiology and Global Health, Umeå University, Sweden. magnus.zingmark@ostersund.se

**Erik Rosendahl**, Department of Community Medicine and Rehabilitation, Physiotherapy, Umeå University, Umeå, Sweden. erik.rosendahl@umu.se

**Marlene Sandlund**, Department of Community Medicine and Rehabilitation, Physiotherapy, Umeå University, Umeå, Sweden. marlene.sandlund@umu.se

**Word count: 4603**



For peer review only - http://bmjopen.bmj.com/site/about/guidelines.xhtml

# ABSTRACT

## Introduction

Exercise interventions have a strong evidence base for falls prevention. However, exercise can be challenging to implement and often has limited reach and poor adherence. Digital technology provides opportunities for both increased access to the intervention and support over time. Further knowledge needs to be gained regarding the effectiveness of completely self-managed digital exercise interventions. The main objective of this study is to compare the effectiveness of a self-managed digital exercise programme, Safe Step, in combination with monthly educational videos with educational videos alone, on falls over one year in older community-dwelling adults.

## Methods and analysis

A two-arm parallel randomized controlled trial will be conducted with at least 1400 community-living older adults (70+ years) who experience impaired balance. Participants will be recruited throughout Sweden with enrolment through the project website. They will be randomly allocated to either the Safe Step exercise programme with additional monthly educational videos about healthy ageing and fall prevention, or the monthly education videos alone. Participants receiving the exercise intervention will be asked to exercise at home for at least 30 minutes, 3 times/week with support of the Safe Step-application. The primary outcome will be rate of falls (fall per person-year). Participants will keep a fall calendar and report falls at the end of each month through a digital questionnaire. Further assessments of secondary outcomes will be made through self-reported questionnaires and a self-test of 30-sec chair stand test at baseline and 3, 6, 9 and 12 months after study start. Data will be analysed according to the intention-to-treat principle.

## Ethics and dissemination

Ethical approval was obtained by The Regional Ethical Review Board in Umeå (Dnr 2018/433-31). Findings will be disseminated through the project web-site, peer-reviewed journals, national and international conferences, and through senior citizen organisations' newsletters.

## Trial registration number

NCT03963570


Bullet points, stenghts and limitations of the study:

- The method of using a completely self-managed digital exercise programme for delivering fall prevention exercise is novel and will contribute to the evidence of using smartphones and tablets for delivering fall prevention exercise.
- The Safe Step-exercise programme includes behaviour change support to facilitate maintenance of the new exercise routine.
- The use of wide inclusion criteria could enhance external validity.
- A limitation of the study is that large attrition rates are expected due to the study design of no face-to-face interaction, and there is a risk of unequal distribution between groups.
- To assess intervention reach, the socio-demographic parameters of participants will be compared to the target population.



For peer review only - http://bmjopen.bmj.com/site/about/guidelines.xhtml

# BACKGROUND

About one-third of community-living older adults fall every year(1, 2), with an increasing frequency with a higher age and frailty level.(3) In Sweden, falls represent 87% of injury-related hospital admissions in older adults(4), with a 30% risk of another fall which requires medical attention in the next five years.(5) Globally, the related health care costs are substantial(6), and is likely to exponentially increase with the growing proportion of older people.(7)

There is strong evidence that falls among community-living older adults can be prevented both short- and long term using exercise as a single intervention.(8, 9) The most effective types of exercise are balance and functional exercises combined with resistance exercises.(9) Even though there is a large body of evidence that supports the effectiveness of exercise interventions, up to 52% of older adults engaging in individually targeted fall prevention exercise do not adhere to the exercise over 12 months.(10)

To aid older adults in changing their exercise behaviour, there is a need for tailored exercise interventions that are not only adapted to the individual's physical function but also promote personal benefits, such as increased or prolonged independence.(11, 12) Furthermore, interventions need to increase motivation for exercise by taking into consideration the older adults' individual lifestyle, perceived risks of exercise, as well as physical capabilities.(13) To support self-efficacy for exercise, older adults can be engaged in creating and tailoring their own routines, which has been suggested as a route to improved adherence.(11) Additionally, support of older adults' active roles are preferable to having complete dependence on professionals,(7) and can also improve well-being among older adults.(14)

Home-based exercise provides a practical choice for participants and could solve issues with both accessibility and costs as well as support the older person to self-manage their exercise. According to a large survey, home-based exercises were preferred over other fall prevention strategies, especially in individuals of an older age, those who have recently fallen, or those who are socially disadvantaged.(15) To exercise at home with occasional visits from instructors has been shown to be effective in preventing falls(16) as well as to integrate the exercise into everyday activities at home(17), which many older adults also prefer.(18)

The use of technology and digital health are emerging as ways of delivering self-managed exercise and fall prevention interventions for older adults.(19-21) Such digital interventions enable the participants to exercise at preferred times and locations.(22, 23) Technology can further enable the use of tools for behaviour change such as self-monitoring, direct feed-back, and action planning, which can support self-efficacy for self-management.(24) Digital technology can, therefore, provide support over time, which is important due to high attrition rates from fall prevention exercise programmes.(10)

There is limited evidence of the effectiveness of smartphones and tablets for delivering fall prevention exercise.(19) Of those evaluated, the interventions has been combined with the interaction of health professionals, e.g.(20, 21). If proven effective, completely self-managed digital interventions with no interaction with a health professional would provide a more rapid and extensive implementation of fall prevention programmes, vastly increasing reach to a wider population. Additionally, it may be economically beneficial due to reduced use of resources from health care professionals. In this emerging field, more research is needed regarding the effectiveness of digital interventions with no interaction between health care professionals and participants.



For peer review only - http://bmjopen.bmj.com/site/about/guidelines.xhtml

We have previously developed a completely self-managed exercise application for smartphones and tablets with a focus on improving physical function and preventing falls, the Safe Step-application.(25) The application provides a large repository of evidence-based exercises in short video formats with associated safety advice. Behaviour change is supported through strengthening of self-efficacy and behaviour change techniques incorporated in the application. The participants are supported to create their individual programme, plan their weekly exercise and monitor their exercise routine in the application. To further support behaviour change, the participants receive motivational feedback and reminders of their planned exercise from a virtual physiotherapist. The exercise programme is influenced by the Otago home exercise programme that has been proven effective in reducing falls among community-living older adults,(26, 27) but also expanded with both easier and more challenging exercises mainly inspired by the Falls Management Exercise (FaME) programme.(28) The Safe Step-application (v1) was explored in a feasibility study, a participant preference trial comparing the programme to a paper booklet with exercises. Results from a qualitative study showed that the participant using the digital exercise programme found it both easy to use and easy to tailor to their own circumstances.(22) The majority of the participants exercised at least during 75% of the intervention weeks and one third of the participants completed more than 75% of recommended exercise time.(29)

To investigate the effectiveness of a self-managed digital exercise programme, this randomised (1:1) controlled trial (RCT) aims to compare the Safe Step-application in combination with monthly educational videos with a control group that only receives the educational videos.

The primary aim is to evaluate the effect on fall rate in older community-dwelling adults during a 12-month intervention period. Secondary aims are to evaluate number of fallers, self-reported balance and strength, fear of falling, quality of life, cost-effectiveness, and attrition and exercise adherence. In addition, we will describe socio-demographic parameters of those who are recruited in comparison to the target population in order to assess intervention reach.

# METHOD

## Participant and Public Involvement

The Safe Step-application has been designed and co-created with, and for older adults.(25) In further co-creation sessions, behaviour change components were developed together with older adults to enhance long-term adherence. The feasibility and acceptability of outcome measures have been informed by a feasibility trial. The ProFaNE consensus of a common data set for fall injury prevention trials(30) and a feasibility study have informed the choice of outcome measures in this RCT. In addition, a small reference group has tested the final questionnaires and provided feedback. A spokesperson for senior citizen organisations in Sweden was consulted regarding recruitment for the RCT-study.

## Study design

The 1-year Safe Step RCT-study uses a two-arm parallel design with digital interventions. The intervention group receives the Safe Step-application, delivered on smartphones or tablets. Both groups will receive an e-mail every month with short educational videos about healthy ageing and falls prevention.

This protocol is reported in line with the Standard Protocol Items: Recommendations for Interventional Trials (SPIRIT) guidelines.(31, 32) The RCT will be reported according to the Consolidated Standards of Reporting Trials (CONSORT) statement,(33) and CONSORT EHEALTH criteria.(34) The health economic evaluation will be reported according to the Consolidated Health



For peer review only - http://bmjopen.bmj.com/site/about/guidelines.xhtml

Economic Evaluation Reporting Standards (CHEERS) statement.(35) The trial was registered prior to study start at ClinicalTrials.gov (NCT03963570).

## Participants

At least 1400 and up to 2000 older adults living in the community will be recruited throughout Sweden. The inclusion and exclusion criteria are shown in table 1.

**Table 1.** Inclusion and exclusion criteria

| Inclusion criteria | Exclusion criteria |
|---|---|
| • Older adults 70 years or older<br>• Fallen or experienced a decline in perceived postural balance during the last year<br>• Have access to a smartphone or tablet and uses it regularly<br>• Have an own e-mail address and uses it<br>• Able to understand verbal and written instructions in Swedish<br>• Can rise from a standard height chair without a person helping<br>• Can walk independently without a walking aid indoors | • Progressive disease where there is likely to be a decline in strength or balance over the next year<br>• Perceived memory dysfunction that affect everyday life activities<br>• Taking part in more than 3 hours each week of strenuous physical exercise which makes them out of breath (e.g. dance, gymnastics, gym exercises, running, or skiing) |

## Recruitment

To reach the target sample size, at least one year is estimated for enrolment of participants starting in Sept 2019. The recruitment strategies will be directed towards the largest senior citizen organisations with the potential to reach approximately 800 000 members, and information will be distributed via the Physiotherapist union, section for older adult's health. Recruitment strategies will include both digital and non-digital strategies. The digital recruitment strategies include advertisements on webpages and Facebook; posts on open and closed Facebook-pages; e-mails or digital newsletters directly to members of the senior citizen organizations. Non-digital strategies include advertisements in newspapers and in Swedish magazines commonly read by older adults. Participants will be directed to the open-access project website (www.sakrasteg.se) where the necessary study information is provided to decide on participation. A self-assessment of eligibility will be performed by the participant based on the inclusion and exclusion criteria provided (table 1). On the project website, participants are informed that by providing their e-mail address they will give their informed consent for participation and consent for publication of their pseudonymised data. An e-mail will be sent to them to confirm their registration and verify their e-mail address, including one reminder. When confirmation has been received a link will be sent to the first baseline questionnaire (figure 1).

For peer review only



For peer review only - http://bmjopen.bmj.com/site/about/guidelines.xhtml

## Randomization and Blinding

After completed baseline questionnaire, the participants will be randomized in a 1:1 ratio using Permuted Block Randomization with randomly varied block sizes. A statistician not involved in the study will generate the allocation list using the randomizeR R-package before enrolment.(36) A computer programme developed in FLOW, Microsoft Office 365-software, will randomize participants according to the allocation list.

After randomization, the FLOW programme will automatically send e-mails with follow-up questionnaires as well as the monthly fall reports and educational videos. In order to include participants, monitor data collection, and answer e-mails regarding technical issues, two members of the research group will be able to see the participant's e-mail addresses and group allocation in the FLOW-system and database. However, no personal contact will occur between researchers and participants regarding the results of their outcome assessments.

Analyses of the outcomes regarding falls and physical function will be made by a researcher blinded to group allocation. Participants will not be blinded due to the nature of the intervention.

## Interventions

After group allocation, the participants will receive an e-mail introducing them to the interventions as described below. Throughout the study there will be a project e-mail account for questions, and frequently asked questions (FAQ) may gradually be added on the website.

Safe Step - digital exercise programme

The Safe Step-application was developed by our research group in collaboration with older adults(25, 37) and the software (v2) was programmed by ICT Services and System Development, Umeå University. Participants allocated to the exercise intervention will receive instructions by e-mail on how to download the application from App Store or Google Play, as well as their personal log-in details.

On the first time use of the application, the participants view an instruction video on how to use the application to create and progress their own individual exercise programme. The participants compose their individual exercise programme choosing 10 exercises from predetermined groups of exercises with a main focus to improve balance (3 groups), increase lower-limb strength (4 groups), and improve gait/step (3 groups). The exercises are presented in video format and in increasing levels of difficulty, or with alternatives within each group of exercises. The participants are instructed to choose exercises that are challenging but not too hard. Hence, during balance exercises a feeling of being unstable but not to the extent of losing their balance and falling, and for strength exercises progress when able to easily perform the recommended repetitions. A new exercise should be selected whenever they feel that the current exercise becomes too easy or too difficult. Participants are requested to exercise for 30 minutes at least three days per week, but are also informed that a longer duration of exercising per week will give a better fall preventive effect. After each session participants will register their activities in the application: completed exercises, exercise duration, experienced well-being of the day of exercise, and experience of levels of intensity for balance and strength exercises.

In the application, the participant can make a schedule for their weekly exercise, register activity, and monitor their exercise routines (figure 2). They will also be able to set reminders tailored to their personal preferences. Direct feedback and motivational messages will be received from a virtual

For peer review only



For peer review only - http://bmjopen.bmj.com/site/about/guidelines.xhtml

physiotherapist when exercise is registered. At the beginning of every month of the 1 year intervention period, the participants will receive an e-mail with a new educational video about healthy ageing and fall-prevention. The third video contains information on physical activity and exercise, but no suggestions of particular exercises are given.

Control group

Participants allocated to the control group will receive the same educational videos as those sent to the intervention group. After the 12-month follow-up assessment, the control group will be offered the Safe Step-application.

## Data collection

At baseline and 3, 6, 9 and 12 months respectively from the participant's inclusion in the study, an e-mail containing a link to an online questionnaire will be sent to the participants. All collected data will be self-reports, including a 30-sec chair stand self-test (30CST). In absence of completed questionnaires, a reminding e-mail will be sent out after one week. If further absence, another e-mail will be sent after an additional week. This reminder will also include a question if they want to withdraw from the study. Data registered in the exercise diary of the application is collected continuously. In the same monthly e-mail as the educational videos, all participants will receive a request to report any falls during the past month by answering a short web-based questionnaire. In the absence of a completed questionnaire, one reminder will be sent after one week. All data retrieved from questionnaires, as well as application data, will be coded and reported on a group level.

## Outcomes

Primary outcome

The primary and secondary outcomes, and time points of distribution are presented in table 2. The primary outcome measure will be the rate of falls (fall per person/year) during the 12 months of the intervention. To register falls, a link to a self-report online questionnaire will be sent monthly by e-mail. To be able to report correctly the coming month, participants are asked to make notes of falls in a calendar, preferably the day the fall has occurred. They will be provided with a printable calendar in the e-mail sent out after allocation, but they can also use their own. If a fall is reported, additional questions will be asked about the cause of the fall, potential injuries sustained, need for medical attention, and hospitalization. The number of persons falling during 12 months will be analysed based on the same data as a secondary outcome.

Falls will be defined as an event in which the person comes to rest inadvertently on the ground or floor, regardless of what caused the fall or if an injury was sustained. Our definition is slightly modified and clarified in comparison with a previous consensus statement.(30) The ProFaNE definition included falls where the person come to rest on an "other lower-level", for example tripping and taking a hold of a dresser.(30) We exclude "lower-level" as our experience from previous studies is that participants seldom perceived such an event as a fall. In accordance with a systematic review,(38) we clarify that a fall should be reported regardless of cause and we also clarify that unintentionally coming to rest on the floor or ground is a fall regardless of injury. Our definition is printed in the online questionnaire.

Secondary outcomes



For peer review only - http://bmjopen.bmj.com/site/about/guidelines.xhtml

Socio-demographic parameters, such as age, sex, health, use of smartphones and tablets, and living conditions will be collected at baseline to understand which older adults who are reached through the intervention and to be able to assess predictors of completion.

Self-reported data regarding physical assessments will be collected at baseline, 3 months, 6 months, 9 months and 12 months respectively. Single item questions with a 5- graded Likert scale are used to assess self-rated improvements in strength and balance (table 2). A 30-sec chair stand test is performed by the participants following instructions in the online questionnaire. The original administration of the test has shown excellent test-retest reliability and criterion validity for assessing functional lower extremity leg strength in older adults,(39) but studies regarding the psychometric properties of a self-test is lacking. Instructions for the participants to be able to perform the 30CST by themselves at home were developed by the research group. After feedback from an advisory group of older adults, the instructions were slightly revised. When performing the test, the participants are asked to place a chair against the wall and take a seat in the middle of the chair with both feet in full contact with the floor and arms folded across the chest. They are instructed to fully stand up and sit down as many times as possible for 30 seconds and register the number of full stands. Participants are asked to use the Borg Scale of Perceived Exertion(40) in combination with the 30CST test to quantify their effort. The perceived exertion is rated on a scale between 6 (none) to 20 (maximal exertion).

The Swedish version of Falls Efficacy Scale-International (FES-I(S))(41) will be used to measure concerns about falling. FES-I is widely used among older adults and is suitable for use in different populations and in clinical trials.(42) The FES-I and FES-I (S) has shown excellent internal and test-retest reliability.(41, 43)

Health-related quality of life will be included in the secondary outcomes to enable us to conduct economic analysis and will be assessed by the European Quality of Life, 5 dimensions (EQ-5D-5L)(44) self-report questionnaire. Cost-effectiveness will be conducted from a societal perspective at 12 months.

Both groups will also be asked to rate their use and perceived usefulness of the monthly delivered educational videos on 4-level ordinal scales.

Additional questions will be asked to the exercise intervention group regarding adherence, experienced positive effects besides strength and balance, and adverse events. Adherence to exercise will be evaluated by self-report questions at follow up assessments through pre-defined alternatives, and will be summarized as exercised min/week. Additionally, adherence to exercise including the proportion meeting at least the predefined minutes of 3x30 min/week, will be collected through an exercise diary integrated within the Safe Step-application.

At self-report assessments at 3, 6, 9 and 12 months after study start, a single item question with predefined answers will be used to assess experienced positive effects of the exercise intervention besides effects on balance and strength.

Adverse events in form of falls occurring while exercising with the Safe Step-application will be monitored monthly. If a participant registers a fall in the monthly questionnaire, there will be a follow-up question of whether the fall occurred while exercising with the Safe Step-programme. If so, a member of the research team will contact the participant to ask him or her about the nature of the fall, and if necessary, suggest measures to prevent further falls during exercise. Additional adverse events of taking part in the intervention (eg. muscle strain) will be collected at assessments at 3, 6, 9 and 12 months after study start, and measured through a single-item question: “Have you



For peer review only - http://bmjopen.bmj.com/site/about/guidelines.xhtml

experienced any negative effects of the exercise intervention?”. The participants will be presented with predefined alternatives and an optional text field.

At the end of the 12-month intervention, the participants in both groups will be asked if they have started to do any other exercise activities or programmes during the intervention period. In addition, self-rated improvements in balance and leg-strength over the whole course of the intervention will be measured through single item questions. Both groups will also be asked to report if they have had any extra cost related to the intervention during the intervention period (e.g. for internet access or equipment) to inform the cost-effectiveness analysis. Drop-outs and withdrawals from the study will be registered throughout the 12-month intervention. Withdrawals will be defined as those who have formally discontinued the study by filling in the withdraw questionnaire. Participants that consistently discontinue to answer the questionnaires will be defined as drop-outs.

## Data storing and management

Participants will be assigned a pseudonymised identifying code (ID) when registering for the study. E-mail addresses will be collected at registration, but no names or social security numbers. Data from the online questionnaires will be directly entered into LimeSurvey software, integrated with a data collection platform, developed and hosted by ITS at Umeå University. Through LimeSurvey, several data quality features have been implemented, for example, obligatory fields and range checks.

The identity of the participants will be separated from research data entries, i.e. questionnaire response and mobile-based exercise diary entries, by storing the identity in a separate database. Access to sensitive information requires a separate set of credentials. ITS at Umeå University provides all web-applications, services and storage for the study. This is provided in accordance with research regulations and GDPR. Data entries will be monitored by one of the researchers throughout the study.

For peer review only



For peer review only - http://bmjopen.bmj.com/site/about/guidelines.xhtml

**Table 2.** List of primary and secondary outcome measures

| Outcome measures | BA | C | M | 3-month | 6-month | 9-month | 12-month |
|---|---|---|---|---|---|---|---|
| Fall rate[1] (fall per person/year) | | | X | | | | |
| Number of fallers[2] (n) | | | X | | | | |
| Number of falls during exercise with Safe Step[3] (n) | | | X | | | | |
| Fall-related injuries[3] (yes/no, need of medical attention, type of injury) | | | X | | | | |
| Hospitalization due to falls[3] (yes/no and number of days) | | | X | | | | |
| Reach[3] (Self-reported socio-demographic parameters such as age, sex, years of education, use of smartphone and tablet, number of cohabitants, and self-reported health and diagnosis) | X | | | | | | |
| Self-administered 30-second chair stand test[4] | X | | | X | X | X | X |
| The Borg Scale of Perceived Exertion during 30-sec chair stand test[5] (numeric scale, score 6-20) | X | | | X | X | X | X |
| Self-rated balance[5], "How do you perceive your balance?" (5-level ordinal scale) | X | | | X | X | X | X |
| Self-rated leg strength[5], "How do you perceive your leg muscle strength?" (5-level ordinal scale) | X | | | X | X | X | X |
| Fear of falling[4] (Falls Efficacy Scale-International, FES-I) | X | | | X | X | X | X |
| Health-related quality of life[4] (EQ-5D-5L) | X | | | X | X | X | X |
| Experienced positive effects of the exercise intervention[3] (nominal scale, optional text field) | X* | | | X* | X* | X* | X* |
| Experienced negative effects of the exercise intervention[3] (nominal scale, optional text field) | X* | | | X* | X* | X* | X* |
| Exercise adherence throughout the intervention reported through an integrated exercise diary in the Safe step-application[3,4] | | X* | | | | | |
| Exercise adherence, self-reported min/week[3] | | | | X* | X* | X* | X* |
| Use and perceived usefulness of educational videos[5] (4-level ordinal scale) | | | | X | X | X | X |
| Self-rated improvements in balance[6], "If you compare with when you started this study a year ago, how would you assess balance today?" (5-level ordinal scale) | | | | | | | X |
| Self-rated improvements in leg-strength[6], "If you compare with when you started this study a year ago, how would you assess the muscle strength in your legs today?" (5-level ordinal scale) | | | | | | | X |

| | | | | | | | |
|---|---|---|---|---|---|---|---|
| Rate of attrition[2] (n) | | | | | | | X |
| New exercise routines[3] (yes/no, text field to describe the exercise, time since initiation (predefined alternatives), minutes/week (predefined alternatives)) | | | | | | | X |
| Physical activity[4] (minutes/week, predefined alternatives) | X | | | | | | X |
| Self-reported costs related to the interventions[3] (amount and what) | | | | | | | X |
| Cost-effectiveness of the interventions with respect to falls[4] | | | | | | | X |

BA=Baseline assessment, C= Continuously, M=Monthly administered, *Only administered to the exercise intervention group, [1]Negative binomial regression, [2]Logistic regression, [3]Descriptive analysis, [4]Linear mixed model, [5]Cumulative link mixed model, [6]Ordinal logistic regression

For peer review only

## Sample size

The sample size calculation for the primary outcome (fall rate over the 12-month intervention period) is based on the review of Sherrington et al.(45) by calculating for a fall rate of 1 fall/person-year. In addition, a measure of over dispersion of 1,2 is used.(21) Thus, we require 420 participants in each group to have an 80% power to detect a 20% reduction in fall rate at a 5% level of significance. We estimate that at least 1400 participants are needed based on a calculated attrition rate of 50% and a contribution of data from drop-outs. Due to uncertain drop-out rate we will include up to 2000 participants.

## Planned statistical analyses

Analyses will be performed according to the intention-to-treat principle. Thus, all available data for each randomized participant will be analysed according to original allocation and regardless of level of participation. The primary outcome, i.e. the number of falls per person-year, will be analysed using negative binomial regression to account for an expected over-dispersion with respect to Poisson distribution.(46) The fall rate ratio of the two groups will be estimated with a 95% confidence interval and presented with a p-value while adjusting for the prognostic baseline covariates of age and sex for the purpose of increasing precision in estimates and statistical power. In addition, unadjusted results will be presented in accordance with recommendations from the CONSORT 2010 statement.(33)

For secondary outcomes on ordinal variables measured quarterly, analysis will be performed using cumulative link mixed models with logit link function and group, time point and group*timepoint interaction as fixed factors and participant as a random factor. In continuously scored secondary variables measured quarterly, general mixed linear models will be used to assess the group difference. Logistic regression models will be used to compare groups on dichotomous outcome measures.

Effects on falls, attrition and self-reported effects on balance and strength will be studied in subgroup analyses in relation to sex, different age-groups, reported health status, physical activity, and use of smartphones and tablets. Further subgroup analysis will examine exercise adherence in the exercise group as well as the dose-response relationships (min/week). Descriptive statistics will be provided to reveal the socio-demographic distribution in the sample in comparison to the population and geographical distribution in urban and rural areas.

Multiple imputation will be performed on variables containing data considered to be missing at random if proportions of missing data does not exceed 40%, as suggested in literature.(47) In addition, analysis on original unimputed data will be presented as supplement. All statistical tests will be two-sided with a significance level of 0.05.

## Economic evaluation

Results will be presented as incremental cost-effectiveness ratio (ICER) after 12 months, i.e., the ratio of the difference in costs and the difference in health effects between the intervention group versus the control group. Data on health effects will be based on the primary outcome falls averted and the secondary outcome health-related quality of life assessed by the EQ-5D-5L. The data on health-related quality of life will be used to calculate quality-adjusted life years (QALYs). Health and social care costs will be estimated by self-reported data on monthly falls and their consequences in combination with previously published data on the specific cost for the most frequent injuries. Resource use will include intervention costs, healthcare costs, and community service costs.



For peer review only - http://bmjopen.bmj.com/site/about/guidelines.xhtml

## Ethics and dissemination

Ethical approval was obtained by The Regional Ethical Review Board in Umeå (Dnr 2018/433-31). The results of the study will be made publicly available through the project web-site, reported at national and international conferences, as well as in peer-reviewed journals, and presented to senior citizen organisations through member journals and digital newsletters. Data from the Safe Step-RCT will be available upon reasonable request when the results have been published in peer-reviewed journals.

# DISCUSSION

To facilitate appropriate exercise to reduce age-related functional decline and prevent falls, attractive and evidence-based interventions are needed that can reach older people, including those who live rurally, or who merely want to self-manage their risk of falling. This study will evaluate the effectiveness of a completely self-managed digital exercise programme for fall prevention with additional educational videos compared to educational videos only.

The Safe Step-application is available for smartphones and tablets and provides evidence-based exercises for the home-environment. Besides conventional exercises, Safe Step inspires the integration of exercises in daily activities, both indoors and outdoors. The approach of integrating exercise into already existing daily routines has shown great potential to reduce the rate of falls among older adults.(17)

To support the older adults to sustain their exercise behaviour over time, Safe step includes a set of behaviour change strategies. The older users will create their individual exercise programme, plan their exercise and monitor their exercise routines in order to support exercise self-management. Self-management of health and exercise has been recommended to improve adherence to fall prevention programmes by strengthening the older adults' self-efficacy.(11) However, delivering exercise by mobile technology increases the complexity of providing individual support. Promisingly, in our previous feasibility study, participants expressed confidence in self-managing their exercise programmes, both in digital format and as a standard paper booklet. Worth noting is that use of the Safe Step-exercise programme seems to have supported the older adults to a greater extent than a paper booklet.(22)

Adherence to individual exercise based fall-prevention is often poor over time.(10) A contributing factor could be a lack of social components and peer support, which has been shown to positively affect attendance and adherence to exercise classes among older adults.(48) However, preferences are diverse among older adults, and some older adults have stated to prefer home-based exercises.(15) Due to the nature of this home-based intervention with online recruitment and an intervention period of one year, an attrition rate of 50% is anticipated. Still, this study will assess the effectiveness of a self-managed digital exercise programme to prevent falls in real-life settings where the older adults themselves will take the initiative to download and use an application. Hence, the attrition rates will be a valuable contribution in understanding more about older adult's exercise adherence when exercising with smartphones and tablets in a home-based setting.

Home-based exercise has been shown to be preferred among older adults of advancing age, those with recent falls, and those with lower socioeconomic status.(15) On one hand, fall prevention interventions delivered by digital technology might create a constraint for older adults who cannot afford digital technology or do not have digital competency. On the other hand, digital technology will present opportunities for older adults that might not have the opportunity or interest in attending community-based programmes or regularly visit health care centres. Additionally, in recent years, the use of digital technology such as smartphones and tablets has increased sharply among older adults. In 2019, 67% of the aged Swedish population used the internet on their smartphone,



For peer review only - http://bmjopen.bmj.com/site/about/guidelines.xhtml

and 40% on their tablet,(49) and the rapid increase in use is expected to continue. In light of these considerations, it is important to explore reach as suggested in the present study to describe which older adults that are interested in exercising with a digital application with no interaction with a health care professional.

Besides effect on falls, this research has the potential to increase knowledge regarding which older adults are interested in a digital self-management exercise intervention, exercise adherence, and effects on self-rated physical function. The design of this study with few inclusion and exclusion criteria and a national recruitment will strengthen external validity in developed countries similar to Sweden with an ageing population and large rural areas. If proven effective and cost-effective, the Safe Step digital exercise programme has the potential to be highly accessible and reduce risk of functional decline and falls.

## Authors' contributions

Original study concept was conceived by MS and LLO. MS, LLO, DS, BP and ER contributed to the design of the study. BP drafted the manuscript with contribution from all authors. MZ provided input on health economics and PL on statistics. All authors read and approved the final version of the manuscript.

## Funding statement

This work was financially supported by the Swedish Research Council (grant number 2015-03481); the Strategic Research Programme in Care Sciences, Umeå University and The Karolinska Institute, Sweden; the Swedish Research Council for Health, Working Life and Welfare (FORTE); and King Gustav V's and Queen Victoria's Freemasons' Foundation.

## Competing interests

The authors declare that they have no competing interest besides that DAS is a Director of Later Life Training, a not for profit training company in the UK that delivers group falls prevention exercise training (Otago and FaME) to health and fitness professionals. The App was developed collaboratively and it will not be sold for profit.

For peer review only

## REFERENCES

1. Tinetti ME, Speechley M, Ginter SF. Risk factors for falls among elderly persons living in the community. *N Engl J Med*. 1988;319(26):1701-7.

2. Campbell AJ, Borrie MJ, Spears GF. Risk factors for falls in a community-based prospective study of people 70 years and older. J *Gerontol.* 1989;44(4):M112-7.

3. Campbell AJ, Robertson MC. Rethinking individual and community fall prevention strategies: a meta-regression comparing single and multifactorial interventions. *Age Ageing.* 2007;36(6):656-62.

4. National Board of Health and Welfare. Statistical database: Yttre orsaker till skador och förgiftningar. Stockholm: Socialstyrelsen [20190926]. Available from: http://www.socialstyrelsen.se/Statistik/statistikdatabas/.

5. Pohl P, Nordin E, Lundquist A, Bergstrom U, Lundin-Olsson L. Community-dwelling older people with an injurious fall are likely to sustain new injurious falls within 5 years--a prospective long-term follow-up study. *BMC Geriatr.* 2014;14:120.

6. Davis JC, Robertson MC, Ashe MC, Liu-Ambrose T, Khan KM, Marra CA. International comparison of cost of falls in older adults living in the community: a systematic review. *Osteoporosis Int.* 2010;21(8):1295-306.



For peer review only - http://bmjopen.bmj.com/site/about/guidelines.xhtml

7. WHO, Department of Ageing and Life Course. WHO Global Report on Falls Prevention in Older Age. Geneva: World Health Organization; 2007.

8. Finnegan S, Seers K, Bruce J. Long-term follow-up of exercise interventions aimed at preventing falls in older people living in the community: a systematic review and meta-analysis. *Physiotherapy.* 2019;105(2):187-199.

9. Sherrington C, Fairhall NJ, Wallbank GK, Tiedemann A, Michaleff ZA, Howard K, et al. Exercise for preventing falls in older people living in the community. *Cochrane Database Syst Rev.* 2019;1:CD012424.

10. Nyman SR, Victor CR. Older people's participation in and engagement with falls prevention interventions in community settings: an augment to the Cochrane systematic review. *Age Ageing.* 2012;41(1):16-23.

11. Yardley L, Beyer N, Hauer K, McKee K, Ballinger C, Todd C. Recommendations for promoting the engagement of older people in activities to prevent falls. *Qual Saf Health Care.* 2007;16(3):230-4.

12. Finnegan S, Bruce J, Seers K. What enables older people to continue with their falls prevention exercises? A qualitative systematic review. *BMJ Open.* 2019;9(4):e026074.

13. Sandlund M, Pohl P, Ahlgren C, Skelton DA, Melander-Wikman A, Bergvall-Kareborn B, et al. Gender Perspective on Older People's Exercise Preferences and



For peer review only - http://bmjopen.bmj.com/site/about/guidelines.xhtml

Motivators in the Context of Falls Prevention: A Qualitative Study. *Biomed Res Int.* 2018;2018:6865156.

14. Cramm JM, Hartgerink JM, Steyerberg EW, Bakker TJ, Mackenbach JP, Nieboer AP. Understanding older patients' self-management abilities: functional loss, self-management, and well-being. *Qual Life Res.* 2013;22(1):85-92.

15. Yardley L, Kirby S, Ben-Shlomo Y, Gilbert R, Whitehead S, Todd C. How likely are older people to take up different falls prevention activities? *Prev Med.* 2008;47(5):554-8.

16. Robertson MC, Campbell AJ, Gardner MM, Devlin N. Preventing injuries in older people by preventing falls: a meta-analysis of individual-level data. *J Am Geriatr Soc.* 2002;50(5):905-11.

17. Weber M, Belala N, Clemson L, Boulton E, Hawley-Hague H, Becker C, et al. Feasibility and Effectiveness of Intervention Programmes Integrating Functional Exercise into Daily Life of Older Adults: A Systematic Review. *Gerontology.* 2018;64(2):172-87.

18. Robinson L, Newton JL, Jones D, Dawson P. Self-management and adherence with exercise-based falls prevention programmes: a qualitative study to explore the views and experiences of older people and physiotherapists. *Disabil Rehabil.* 2014;36(5):379-86.

19. Hamm J, Money AG, Atwal A, Paraskevopoulos I. Fall prevention intervention technologies: A conceptual framework

For peer review only

and survey of the state of the art. *J Biomed Inform.* 2016;59:319-45.

20. van Het Reve E, Silveira P, Daniel F, Casati F, de Bruin ED. Tablet-based strength-balance training to motivate and improve adherence to exercise in independently living older people: part 2 of a phase II preclinical exploratory trial. *J Med Internet Res.* 2014;16(6):e159.

21. Delbaere K, Valenzuela T, Woodbury A, Davies T, Yeong J, Steffens D, et al. Evaluating the effectiveness of a home-based exercise programme delivered through a tablet computer for preventing falls in older community-dwelling people over 2 years: study protocol for the Standing Tall randomised controlled trial. *BMJ Open.* 2015;5(10):e009173.

22. Pettersson B, Wiklund M, Janols R, Lindgren H, Lundin-Olsson L, Skelton DA, et al. 'Managing pieces of a personal puzzle' - Older people's experiences of self-management falls prevention exercise guided by a digital program or a booklet. *BMC Geriatr.* 2019;19(1):43.

23. Hawley-Hague H, Boulton E, Hall A, Pfeiffer K, Todd C. Older adults' perceptions of technologies aimed at falls prevention, detection or monitoring: a systematic review. *Int J Med Inform.* 2014;83(6):416-26.

24. Lorig K, Laurent DD, Plant K, Krishnan E, Ritter PL. The components of action planning and their associations with behavior and health outcomes. *Chronic Illn.* 2014;10(1):50-9.



For peer review only - http://bmjopen.bmj.com/site/about/guidelines.xhtml

25. Sandlund M, Lindgren H, Pohl P, Melander-Wikman A, Bergvall-Kåreborn B, L L-O. Towards a mobile exercise application to prevent falls: a participatory design process. *Int J Child Health Hum Dev*. 2016;9(3).

26. Gardner MM, Buchner DM, Robertson MC, Campbell AJ. Practical implementation of an exercise-based falls prevention programme. *Age Ageing.* 2001;30(1):77-83.

27. Skelton D, Dinan S, Campbell M, Rutherford O. Tailored group exercise (Falls Management Exercise -- FaME) reduces falls in community-dwelling older frequent fallers (an RCT). *Age Ageing*. 2005;34(6):636-9.

28. Skelton DA, Dinan SM. Exercise for falls management: Rationale for an exercise programme aimed at reducing postural instability. *Physiother Theory Pract.* 1999;15(2):105-20.

29. Mansson L, Lundin-Olsson L, Skelton A, Janols R, Lindgren H, Rosendahl E, et al. Older adults preferences for, adherence to and experiences of two self-management falls prevention home exercise programmes: A comparison between a digital programme and a paper booklet, 19 March 2020, PREPRINT (Version 2) available at Research Square [+https://doi.org/10.21203/rs.2.12118/v2+].

30. Lamb SE, Jorstad-Stein EC, Hauer K, Becker C, Prevention of Falls Network E, Outcomes Consensus G. Development of a common outcome data set for fall injury

For peer review only

prevention trials: the Prevention of Falls Network Europe consensus. *J Am Geriatr Soc.* 2005;53(9):1618-22.

31. Chan AW, Tetzlaff JM, Altman DG, Laupacis A, Gotzsche PC, Krleza-Jeric K, et al. SPIRIT 2013 Statement: Defining Standard Protocol Items for Clinical Trials. *Ann Intern Med.* 2013;158(3):200-+.

32. Chan AW, Tetzlaff JM, Gotzsche PC, Altman DG, Mann H, Berlin JA, et al. SPIRIT 2013 explanation and elaboration: guidance for protocols of clinical trials. *BMJ.* 2013;346.

33. Schulz KF, Altman DG, Moher D, Group C. CONSORT 2010 statement: updated guidelines for reporting parallel group randomised trials. *BMJ.* 2010;340:c332.

34. Eysenbach G, Group C-E. CONSORT-EHEALTH: improving and standardizing evaluation reports of Web-based and mobile health interventions. *J Med Internet Res.* 2011;13(4):e126.

35. Husereau D, Drummond M, Petrou S, Carswell C, Moher D, Greenberg D, et al. Consolidated Health Economic Evaluation Reporting Standards (CHEERS) statement. *BMJ.* 2013;346:f1049.

36. Uschner D, Hilgers RD, Schindler D, Heussen N. randomizeR: An R Package for the Assessment and Implementation of Randomization in Clinical Trials. *J Stat Softw.* 2018;85(8):1-22.

For peer review only

37. Lindgren H, Lundin-Olsson L, Pohl P, Sandlund M. End users transforming experiences into formal information and process models for personalised health interventions. *Stud Health Technol Inform.* 2014;205:378-82.

38. Hauer K, Lamb SE, Jorstad EC, Todd C, Becker C, Group P. Systematic review of definitions and methods of measuring falls in randomised controlled fall prevention trials. *Age Ageing.* 2006;35(1):5-10.

39. Jones CJ, Rikli RE, Beam WC. A 30-s chair-stand test as a measure of lower body strength in community-residing older adults. *Res Q Exerc Sport.* 1999;70(2):113-9.

40. Borg GA. Psychophysical bases of perceived exertion. *Med Sci Sports Exerc.* 1982;14(5):377-81.

41. Nordell E, Andreasson M, Gall K, Thorngren K. Evaluating the Swedish version of the Falls Efficacy Scale-International (FES-I). *Adv Physiother.* 2009;11(2):81-7.

42. Kempen GI, Todd CJ, Van Haastregt JC, Zijlstra GA, Beyer N, Freiberger E, et al. Cross-cultural validation of the Falls Efficacy Scale International (FES-I) in older people: results from Germany, the Netherlands and the UK were satisfactory. *Disabil Rehabil.* 2007;29(2):155-62.

43. Yardley L, Beyer N, Hauer K, Kempen G, Piot-Ziegler C, Todd C. Development and initial validation of the Falls Efficacy Scale-International (FES-I). *Age Ageing.* 2005;34(6):614-9.



For peer review only - http://bmjopen.bmj.com/site/about/guidelines.xhtml

44. Herdman M, Gudex C, Lloyd A, Janssen M, Kind P, Parkin D, et al. Development and preliminary testing of the new five-level version of EQ-5D (EQ-5D-5L). *Qual Life Res.* 2011;20(10):1727-36.

45. Sherrington C, Michaleff ZA, Fairhall N, Paul SS, Tiedemann A, Whitney J, et al. Exercise to prevent falls in older adults: an updated systematic review and meta-analysis. *Br J Sports Med*. 2017;51(24):1750-8.

46. Robertson MC, Campbell AJ, Herbison P. Statistical analysis of efficacy in falls prevention trials. *J Gerontol a-Biol.* 2005;60(4):530-4.

47. Jakobsen JC, Gluud C, Wetterslev J, Winkel P. When and how should multiple imputation be used for handling missing data in randomised clinical trials - a practical guide with flowcharts. *BMC Med Res Methodol.* 2017;17.

48. Hawley-Hague H, Horne M, Campbell M, Demack S, Skelton DA, Todd C. Multiple levels of influence on older adults' attendance and adherence to community exercise classes. *Gerontologist.* 2014;54(4):599-610.

49. Internetstiftelsen. Svenskarna och internet 2019. Available from: https://svenskarnaochinternet.se/app/uploads/2019/10/svenskarna-och-internet-2019-a4.pdf; [Accessed: Nov 2019].

For peer review only



For peer review only - http://bmjopen.bmj.com/site/about/guidelines.xhtml

**Figure 1.** Flow chart of study design.

**Figure 2.** Features of the Safe Step-application

For peer review only



For peer review only - http://bmjopen.bmj.com/site/about/guidelines.xhtml

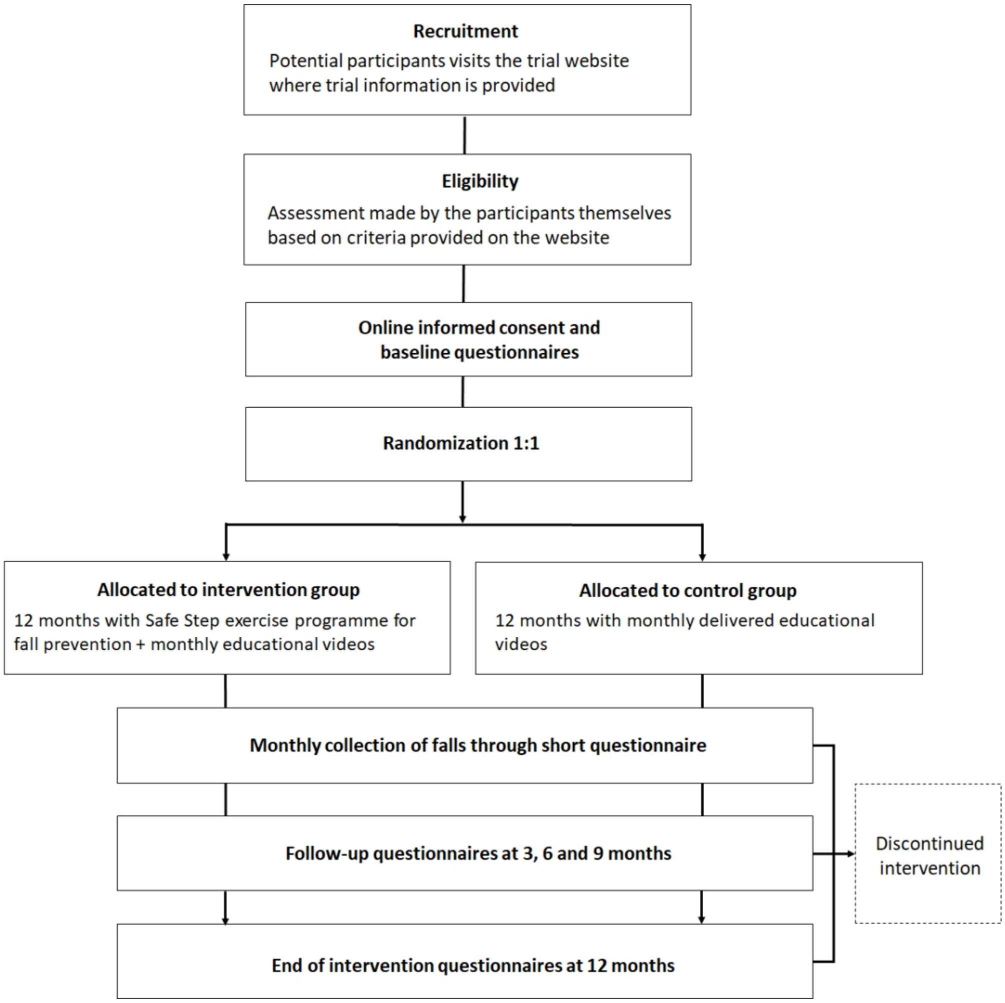


Figure 1. Flow chart of study design.

103x102mm (300 x 300 DPI)

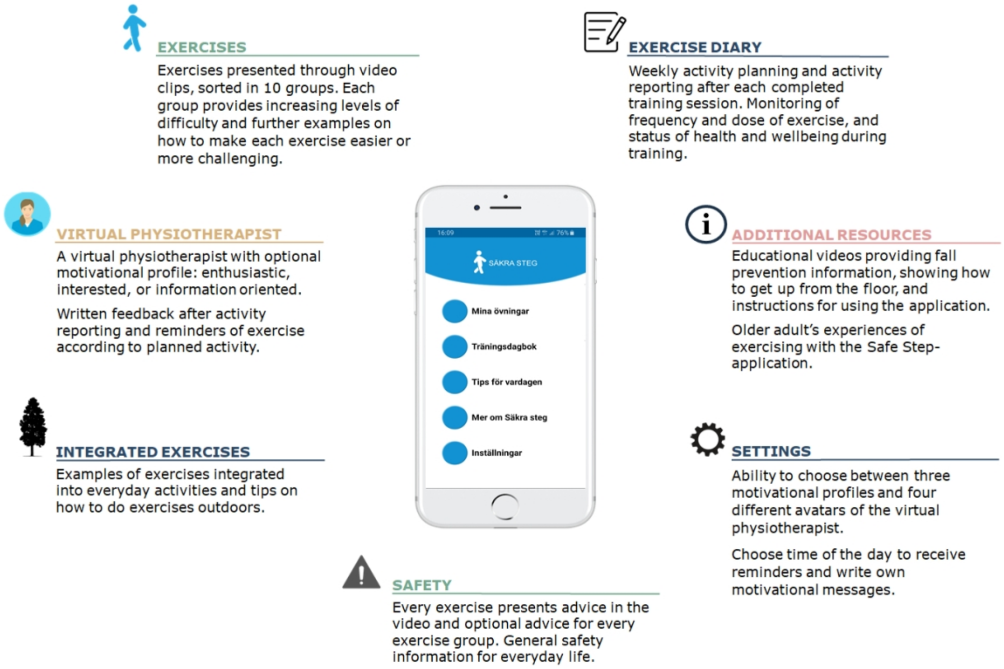


Figure 2. Features of the Safe Step-application.

115x82mm (300 x 300 DPI)

SPIRIT
STANDARD PROTOCOL ITEMS: RECOMMENDATIONS FOR INTERVENTIONAL TRIALS

SPIRIT 2013 Checklist: Recommended items to address in a clinical trial protocol and related documents*

| Section/item | Item No | Description | Addressed on page number |
|---|---|---|---|
| **Administrative information** | | | |
| Title | 1 | Descriptive title identifying the study design, population, interventions, and, if applicable, trial acronym | 1 |
| Trial registration | 2a | Trial identifier and registry name. If not yet registered, name of intended registry | Abstract page 2, and p. 5, line 152-153 |
| | 2b | All items from the World Health Organization Trial Registration Data Set | Yes |
| Protocol version | 3 | Date and version identifier | Re-submitted manuscript |
| Funding | 4 | Sources and types of financial, material, and other support | p. 14, line 441-445 |
| Roles and responsibilities | 5a | Names, affiliations, and roles of protocol contributors | p. 14, line 435-439 |
| | 5b | Name and contact information for the trial sponsor | p.1, Marlene Sandlund PI |
| | 5c | Role of study sponsor and funders, if any, in study design; collection, management, analysis, and interpretation of data; writing of the report; and the decision to submit the report for publication, including whether they will have ultimate authority over any of these activities | p. 14, line 441-445 |

For peer review only

| | | | |
|---|---|---|---|
| | 5d | Composition, roles, and responsibilities of the coordinating centre, steering committee, endpoint adjudication committee, data management team, and other individuals or groups overseeing the trial, if applicable (see Item 21a for data monitoring committee) | ______________ |
| **Introduction** | | | |
| Background and rationale | 6a | Description of research question and justification for undertaking the trial, including summary of relevant studies (published and unpublished) examining benefits and harms for each intervention | p. 3 |
| | 6b | Explanation for choice of comparators | p. 3 |
| Objectives | 7 | Specific objectives or hypotheses | p. 4, line 127-131 |
| Trial design | 8 | Description of trial design including type of trial (eg, parallel group, crossover, factorial, single group), allocation ratio, and framework (eg, superiority, equivalence, noninferiority, exploratory) | p. 4, line 124-126; p. 4, line 144-147 |
| **Methods: Participants, interventions, and outcomes** | | | |
| Study setting | 9 | Description of study settings (eg, community clinic, academic hospital) and list of countries where data will be collected. Reference to where list of study sites can be obtained | p. 5, line 155-157 |
| Eligibility criteria | 10 | Inclusion and exclusion criteria for participants. If applicable, eligibility criteria for study centres and individuals who will perform the interventions (eg, surgeons, psychotherapists) | Table 1 |
| Interventions | 11a | Interventions for each group with sufficient detail to allow replication, including how and when they will be administered | p. 7, line 193-229 |
| | 11b | Criteria for discontinuing or modifying allocated interventions for a given trial participant (eg, drug dose change in response to harms, participant request, or improving/worsening disease) | p. 7, line 233-236; p. 8, line 302-304 |
| | 11c | Strategies to improve adherence to intervention protocols, and any procedures for monitoring adherence (eg, drug tablet return, laboratory tests) | p. 6, line 218-221; p. 7, line 233-236; p. 8, line 291-296 |



For peer review only - http://bmjopen.bmj.com/site/about/guidelines.xhtml

| | | | |
|---|---|---|---|
| | 11d | Relevant concomitant care and interventions that are permitted or prohibited during the trial | ______________ |
| Outcomes | 12 | Primary, secondary, and other outcomes, including the specific measurement variable (eg, systolic blood pressure), analysis metric (eg, change from baseline, final value, time to event), method of aggregation (eg, median, proportion), and time point for each outcome. Explanation of the clinical relevance of chosen efficacy and harm outcomes is strongly recommended | p. 7, line 242-317 |
| Participant timeline | 13 | Time schedule of enrolment, interventions (including any run-ins and washouts), assessments, and visits for participants. A schematic diagram is highly recommended (see Figure) | Figure 1 |
| Sample size | 14 | Estimated number of participants needed to achieve study objectives and how it was determined, including clinical and statistical assumptions supporting any sample size calculations | p. 12, line 335-342 |
| Recruitment | 15 | Strategies for achieving adequate participant enrolment to reach target sample size | p. 5, line 161-176 |
| **Methods: Assignment of interventions (for controlled trials)** | | | |
| Allocation: | | | |
| Sequence generation | 16a | Method of generating the allocation sequence (eg, computer-generated random numbers), and list of any factors for stratification. To reduce predictability of a random sequence, details of any planned restriction (eg, blocking) should be provided in a separate document that is unavailable to those who enrol participants or assign interventions | p. 6, line 178-183 |
| Allocation concealment mechanism | 16b | Mechanism of implementing the allocation sequence (eg, central telephone; sequentially numbered, opaque, sealed envelopes), describing any steps to conceal the sequence until interventions are assigned | p. 6, line 178-183 |
| Implementation | 16c | Who will generate the allocation sequence, who will enrol participants, and who will assign participants to interventions | p. 6, line 178-185 |
| Blinding (masking) | 17a | Who will be blinded after assignment to interventions (eg, trial participants, care providers, outcome assessors, data analysts), and how | p. 6, line 185-191 |
| | 17b | If blinded, circumstances under which unblinding is permissible, and procedure for revealing a participant's allocated intervention during the trial | ______________ |

**Methods: Data collection, management, and analysis**

| | | | |
|---|---|---|---|
| Data collection methods | 18a | Plans for assessment and collection of outcome, baseline, and other trial data, including any related processes to promote data quality (eg, duplicate measurements, training of assessors) and a description of study instruments (eg, questionnaires, laboratory tests) along with their reliability and validity, if known. Reference to where data collection forms can be found, if not in the protocol | p.7, line 230-241; p.7. line 242; p. 9, line 322-223; table 2 |
| | 18b | Plans to promote participant retention and complete follow-up, including list of any outcome data to be collected for participants who discontinue or deviate from intervention protocols | p. 5, line 174-175; p. 7 line 230-241 |
| Data management | 19 | Plans for data entry, coding, security, and storage, including any related processes to promote data quality (eg, double data entry; range checks for data values). Reference to where details of data management procedures can be found, if not in the protocol | p. 9, line 318-329 |
| Statistical methods | 20a | Statistical methods for analysing primary and secondary outcomes. Reference to where other details of the statistical analysis plan can be found, if not in the protocol | p. 12, line 343-367; p. 12, line 368-376; table 2 |
| | 20b | Methods for any additional analyses (eg, subgroup and adjusted analyses) | p. 12, line 358-363 |
| | 20c | Definition of analysis population relating to protocol non-adherence (eg, as randomised analysis), and any statistical methods to handle missing data (eg, multiple imputation) | p. 12, line 344 and line 364-367 |

**Methods: Monitoring**

| | | | |
|---|---|---|---|
| Data monitoring | 21a | Composition of data monitoring committee (DMC); summary of its role and reporting structure; statement of whether it is independent from the sponsor and competing interests; and reference to where further details about its charter can be found, if not in the protocol. Alternatively, an explanation of why a DMC is not needed | ______________ |
| | 21b | Description of any interim analyses and stopping guidelines, including who will have access to these interim results and make the final decision to terminate the trial | p. 6, line 185-189; p. 8, line 300-308 |
| Harms | 22 | Plans for collecting, assessing, reporting, and managing solicited and spontaneously reported adverse events and other unintended effects of trial interventions or trial conduct | p. 8, line 300-308 |

| | | | |
|---|---|---|---|
| Auditing | 23 | Frequency and procedures for auditing trial conduct, if any, and whether the process will be independent from investigators and the sponsor | _______________ |
| **Ethics and dissemination** | | | |
| Research ethics approval | 24 | Plans for seeking research ethics committee/institutional review board (REC/IRB) approval | Abstract page 2 and p. 13, line 378 |
| Protocol amendments | 25 | Plans for communicating important protocol modifications (eg, changes to eligibility criteria, outcomes, analyses) to relevant parties (eg, investigators, REC/IRBs, trial participants, trial registries, journals, regulators) | _______________ |
| Consent or assent | 26a | Who will obtain informed consent or assent from potential trial participants or authorised surrogates, and how (see Item 32) | p. 5, line 161-176 |
| | 26b | Additional consent provisions for collection and use of participant data and biological specimens in ancillary studies, if applicable | _______________ |
| Confidentiality | 27 | How personal information about potential and enrolled participants will be collected, shared, and maintained in order to protect confidentiality before, during, and after the trial | p. 9, line 318-329 |
| Declaration of interests | 28 | Financial and other competing interests for principal investigators for the overall trial and each study site | p. 14, line 447-451 |
| Access to data | 29 | Statement of who will have access to the final trial dataset, and disclosure of contractual agreements that limit such access for investigators | p. 13, line 381-383 |
| Ancillary and post-trial care | 30 | Provisions, if any, for ancillary and post-trial care, and for compensation to those who suffer harm from trial participation | _______________ |
| Dissemination policy | 31a | Plans for investigators and sponsor to communicate trial results to participants, healthcare professionals, the public, and other relevant groups (eg, via publication, reporting in results databases, or other data sharing arrangements), including any publication restrictions | p. 13, line 378-381 |
| | 31b | Authorship eligibility guidelines and any intended use of professional writers | _______________ |
| | 31c | Plans, if any, for granting public access to the full protocol, participant-level dataset, and statistical code | p. 13, line 381-383 |

For peer review

**Appendices**

| | | | |
|---|---|---|---|
| Informed consent materials | 32 | Model consent form and other related documentation given to participants and authorised surrogates | ______________ |
| Biological specimens | 33 | Plans for collection, laboratory evaluation, and storage of biological specimens for genetic or molecular analysis in the current trial and for future use in ancillary studies, if applicable | ______________ |

*It is strongly recommended that this checklist be read in conjunction with the SPIRIT 2013 Explanation & Elaboration for important clarification on the items. Amendments to the protocol should be tracked and dated. The SPIRIT checklist is copyrighted by the SPIRIT Group under the Creative Commons "Attribution-NonCommercial-NoDerivs 3.0 Unported" license.

For peer review only
