# Supplementary material for: Effectiveness of a self-managed digital exercise programme to prevent falls in older community-dwelling adults: study protocol for the Safe Step randomised controlled trial
Source: BMJ Open. 2020 May 17;10(5):e036194. doi: 10.1136/bmjopen-2019-036194 (PMC7239551; doi:10.1136/bmjopen-2019-036194)
Supplement: Reviewer comments [file bmjopen-2019-036194.reviewer_comments.pdf]

PEER REVIEW HISTORY

BMJ Open publishes all reviews undertaken for accepted manuscripts. Reviewers are asked to complete a checklist review form (**http://bmjopen.bmj.com/site/about/resources/checklist.pdf**) and are provided with free text boxes to elaborate on their assessment. These free text comments are reproduced below.

ARTICLE DETAILS

**TITLE (PROVISIONAL)**	Effectiveness of a self-managed digital exercise programme to prevent falls in older community-dwelling adults –study protocol for the Safe Step randomized controlled trial
**AUTHORS**	Pettersson, Beatrice; Lundin-Olsson, Lillemor; Skelton, Dawn; Liv, Per; Zingmark, Magnus; Rosendahl, Erik; Sandlund, Marlene

VERSION 1 - REVIEW

**REVIEWER**	Chris Rissel<br>NSW Ministry of Health<br>Australia
**REVIEW RETURNED**	07-Jan-2020

**GENERAL COMMENTS**	The introduction did not talk much about the social components of group programs, and how this supports regular attendance and maintenance of new behaviours. This is relevant for digital programs as much as face to face programs.<br>Recruitment sections states that most of the recruitment will be through newsletters etc, and included "social media". Given this is a digital intervention, then more effort should go into on-line recruitment, and the idea of 'social media' needs to be expanded, with an on-line recruitment strategy developed.<br>How much information about the intervention will be on the project web-site? This could be a source of contamination for the control group. What will be explained to prospective participants about the randomisation process? How much information for intervention group participants will be hosted on the web-site relative to what is email to them?<br>Why is the recommendation for physical activity only 30 minutes a day on three days a week? My understanding was that an hour was recommended for falls prevention benefits. Some explanation is needed - Is this because this is considered achievable by most? Perhaps asking for 60 minutes a day for three days might actually achieve 30 minutes of exercises?<br>Presumably the app has been pilot-tested with the target group already? What compliance is there with the app (in terms of entering each session of exercise) or using all the features of the app? More information about the anticipated successful use of the app is needed, and potentially preliminary data on outcomes from a pilot study (if available).<br>The control group only gets emailed links to educational videos. This is quite a different experience to having to download and use an app. It's quite possible that a well designed series of emails with high quality exercise videos would achieve good falls prevention

	outcomes. A future design might be a 2X2 design to consider other ways of delivering a digital program (that didn't require use of an app). This could be considered a suggestion for future research.<br>The anticipated high dropout is a problem for this app/digital intervention. Those who complete are likely to be different to those who drop out – what are the predictors of completion? What other strategies could be used to increase retention?<br>Dissemination includes 'presented to senior citizens organizations'. In addition, lay versions should be distributed to the same newsletters the authors used for recruitment, and there should be an on-line strategy for the dissemination of the results.<br>The English is very good, but there are a few places where a native English speaker could improve the grammar.

**REVIEWER**	D Steffans<br>Royal Prince Alfred Hospital - Australia.
**REVIEW RETURNED**	06-Feb-2020

**GENERAL COMMENTS**	Interesting and well written manuscript. Followed guidelines and described the study methodology in details.

**REVIEWER**	Mittaz Hager Anne-Gabrielle<br>Haute Ecole de Santé HES-SO Valais-Wallis<br>Switzerland
**REVIEW RETURNED**	14-Feb-2020

**GENERAL COMMENTS**	Gratulations for your protocol. I suggest minor improvements:<br>Because you work with older people, you know that these people may encounter some adverse incidents / events at the age. It will be interesting to explain in your protocol (Method) how you will treat these during the study.<br>I'm sure you thought about confounding factors, that could influence the effectiveness of the Safe Step digital exercise programme. It will be nice to introduce these in your protocol, whether in the statistical analysis, whether in your discussion.

VERSION 1 – AUTHOR RESPONSE

Comment	Answer	Page number and lines
Reviewer 1: Chris Rissel		
The introduction did not talk much about the social components of group programs, and how this supports regular attendance and maintenance of new behaviours. This is relevant for digital programs as much as face to face programs.	We understand and agree that the social component is important for both adherence and maintenance of new exercise behaviour. In the discussion, we have acknowledged the potential consequences on adherence of lacking social support. However, since social support of group	Page 13, line 406-410

	programs is not included in our intervention and due to word limitations, we have chosen other focus areas for our introduction.<br>The virtual physiotherapist is present in the digital exercise program to confirm the participants to some extent.	
Recruitment sections states that most of the recruitment will be through newsletters etc, and included “social media”. Given this is a digital intervention, then more effort should go into on-line recruitment, and the idea of ‘social media’ needs to be expanded, with an on-line recruitment strategy developed.	We have clarified the recruitment strategies, especially regarding social media recruitment strategies.	Page 5, line 162-169
How much information for intervention group participants will be hosted on the web-site relative to what is email to them?	The necessary information to decide on participation in the study is presented on the project's website. The e-mail sent to participants after randomization serves as a reminder of what is specific to the intervention to which they were assigned. We have clarified this in the main text.	Page 5, line 169-171
Why is the recommendation for physical activity only 30 minutes a day on three days a week? My understanding was that an hour was recommended for falls prevention benefits. Some explanation is needed - Is this because this is considered achievable by most? Perhaps asking for 60 minutes a day for three days might actually achieve 30 minutes of exercises?	We believe that to exercise for 60 minutes per session, 3 times per week would be a too large commitment. Adherence rates to fall-prevention exercise interventions are poor, and we strive for a duration achievable for most older adults. The participants are presented with the information that these 30 minutes per session at least 3 times per week is a recommended minimum, and that a longer exercise duration have proven more effective, as stated on page 6, line 212-215.<br>30 min per session is also a timespan used within the Otago exercise program, which has been proven effective in preventing falls among older adults.	
Presumably the app has been pilot-tested with the target group already? What compliance is there with the app (in terms of entering each session of exercise) or using all the features of the app? More information about the anticipated successful use of the app is needed, and potentially preliminary data on outcomes from a pilot study (if available).	Yes, the Safe Step-program version 1, which was a web-application, has been pilot-tested in a feasibility study. Version 2, which is an application for smartphones and tablets only is used in the RCT. How exactly the features of the digital program were used is not quantitatively evaluated as log-ins and behaviour in the app is not logged. Results regarding the adherence in	Page 4, line 117-123

	the feasibility study is presented in an article currently under review. We have expanded the introduction with results regarding the adherence and the participants experiences of using the Safe Step-exercise programme.	
The control group only gets emailed links to educational videos. This is quite a different experience to having to download and use an app. It's quite possible that a well-designed series of emails with high quality exercise videos would achieve good falls prevention outcomes. A future design might be a 2X2 design to consider other ways of delivering a digital program (that didn't require use of an app). This could be considered a suggestion for future research.	We believe that e.g. delivering exercise videos by an e-mail programme would not give the same opportunity to create an individualized exercise program. A digital application provides more opportunities for self-management of exercise, and more possibilities for incorporation of methods for behaviour change.<br>A possibility for an alternative design could be to provide the educational videos by an application as well, but this is not investigated in the present study.	
The anticipated high dropout is a problem for this app/digital intervention. Those who complete are likely to be different to those who drop out – what are the predictors of completion? What other strategies could be used to increase retention?	We are aware that this is a potential problem. In this study, we have tried to mimic the reality of where the application will be used. The recruitment and dropout are two important aspects that we aim to investigate. We are very interested in which older adults that will complete this kind of study and we have tried to clarify that this will be studied though sociodemographic parameters in Outcome measures.<br>Parallel to this completely online study, we have an ongoing study were more support are offered to the participants. The support is offered in person through support groups, both regarding the technology and the exercise. This will enable us to make further evaluations regarding predictors of completion.	Page 8, line 263-265
Dissemination includes 'presented to senior citizens organizations'. In addition, lay versions should be distributed to the same newsletters the authors used for recruitment, and there should be an on-line strategy for the dissemination of the results.	Thank you for pointing this out. This is our intention, and the dissemination have been revised accordingly.	Page 2, line 46-47 and page 13, line 379-382
Reviewer 2: D Steffans		
Interesting and well written manuscript. Followed guidelines and described the	Thank you for your comments.	

study methodology in details.

Reviewer 3: Anne-Gabrielle Mittaz Hager

Because you work with older people, you know that these people may encounter some adverse incidents / events at the age. It will be interesting to explain in your protocol (Method) how you will treat these during the study.	Adverse events will be continuously monitored throughout the study as stated in the manuscript. But, we agree with you that how they will be treated should be more clearly explained and this have been clarified in the main text. In the case of a fall during exercise, we will make contact by e-mail and inquire how the event has occurred and, if necessary, suggest measures during exercise to prevent further falls. The study does not have an Independent Data Monitoring Group monitoring safety.	Page 8, line 302-304
I'm sure you thought about confounding factors, that could influence the effectiveness of the Safe Step digital exercise programme. It will be nice to introduce these in your protocol, whether in the statistical analysis, whether in your discussion.	Thank you for your thoughtful comment. As we have stated on page 12, line 348-350, the analysis will be adjusted for the baseline variables sex and age, that are variables known to predict the main outcome of falls.<br>We have consulted our statistician again regarding your issue. While the planned statistical analysis does allow for baseline group differences in covariates influencing the outcome, it is true that its performance in terms of power and precision will improve if adjusting for them (Senn S. Seven myths of randomisation in clinical trials. Stat Med. 2013;32(9):1439-50). In available baseline data, we considered sex and age to be the most predictive variables and therefore chose to pre-specify them in the protocol as planned covariates the statistical models.	
